# The Influence of the Alkylamino Group on the Solvatochromic Behavior of 5-(4-substituted-arylidene)-1,3-dimethylpyrimidine-2,4,6-triones: Synthesis, Spectroscopic and Computational Studies

**DOI:** 10.3390/ma17102447

**Published:** 2024-05-19

**Authors:** Ilona Pyszka, Przemysław Krawczyk, Beata Jędrzejewska

**Affiliations:** 1 Faculty of Chemical Technology and Engineering, Bydgoszcz University of Science and Technology, Seminaryjna 3, 85-326 Bydgoszcz, Poland; ilona.pyszka@pbs.edu.pl; 2 Faculty of Pharmacy, Collegium Medicum, Nicolaus Copernicus University, Kurpińskiego 5, 85-950 Bydgoszcz, Poland; przemekk@cm.umk.pl

**Keywords:** merocyanine dyes, spectroscopic properties, charge transfer, solvatochromism, catalán solvent polarity scale, time-dependent density functional theory calculations

## Abstract

Advances in electronics and medical diagnostics have made organic dyes extremely popular as key functional materials. From a practical viewpoint, it is necessary to assess the spectroscopic and physicochemical properties of newly designed dyes. In this context, the condensation of 1,3-dimethylbarbituric acid with electron-rich alkylaminobenzaldehyde derivatives has been described, resulting in a series of merocyanine-type dyes. These dyes exhibit intense blue-light absorption but weak fluorescence. An electron-donating alkylamino group at position C4 is responsible for the solvatochromic behavior of the dyes since the lone electron pair of the nitrogen atom is variably delocalized toward the barbituric ring, which exhibits electron-withdrawing properties. This was elucidated, taking into account the different geometry of the amino group. The intramolecular charge transfer in the molecules is responsible for the relatively high redshift in absorption and fluorescence spectra. Additionally, an increase in solvent polarity moves the absorption and fluorescence to lower energy regions. The observed solvatochromism is discussed in terms of the four-parameter Catalán solvent polarity scale. The differences in the behavior of the dyes were quantified with the aid of time-dependent density functional theory calculations. The obtained results made it possible to find regularities linking the basic spectroscopic properties of the compounds with their chemical structure. This is important in the targeted search for new, practically important dyes.

## 1. Introduction

Designing, obtaining, and understanding the spectroscopic behavior of new dyes in various environments (pure solvents and molecular assemblies) are necessary prerequisites for further research into technological applications using the interaction of light with matter.

Merocyanine dyes belong to push–pull compounds containing an electron donor end group and electron acceptor moiety separated by a conjugated polymethine chain. The nature of these groups, the number of methine units, as well as the polarity of the environment, influence the efficiency of the intramolecular charge transfer (ICT) process. Consequently, the electronic excitation of these compounds can cause either a sharp increase or decrease in their dipole moment. Therefore, the spectral and fluorescent properties of merocyanines are overly sensitive to changes in their chemical structure and solvent polarity. For this reason, these dyes are widely used in various fields of science and engineering, utilizing light absorption and emissions [1,2,3,4,5]. Different classes of dyes based on barbituric acid found applications as new materials for nonlinear optics (NLOs) and optoelectronics, photosensitizers in photodynamic therapy, radiosensitizers, diagnostic agents in medicine, potentiometric sensors [6,7], fluorescent probes, and photoinitiators [8]. For example, the derivatives of 5-(benzylidene)-1,3-dimethylpyrimidine-2,4,6(1*H*,3*H*,5*H*)-trione demonstrated potent antiproliferative activity against ovarian and breast cancer cell lines, indicating their potential for the development of clinical candidates to treat solid tumors [9]. In vitro and in silico studies also showed that these derivatives interacted with ctDNA, suggesting their potential for targeted therapy [10]. The 5-(arylmethylidene)-2,4,6-pyrimidine-2,4,6(1*H*,3*H*,5*H*)-triones tested for antimicrobial activity against Staphylococcus and Streptococcus bacteria strains may find pharmaceutical applications in antimicrobial treatments [11]. The 5-aryl-4-(arylethynyl)pyrimidines, which can be derived from 5-(arylmethylidene)-2,4,6-pyrimidine-2,4,6(1*H*,3*H*,5*H*)-triones, exhibited fluorescent properties, indicating potential applications in materials science [12].

By understanding the effect of substitution on spectral properties, a new class of dyes with desired properties can be designed for science and engineering applications involving light absorption and emissions. Thus, one goal of our work is to find regularities linking the absorption and emission properties of the tested barbituric acid derivatives with their chemical structure, which is important for the targeted search for new, practically important dyes. The second aspect of this research concerns the assessment of their solvatochromic properties. 

Solvatochromism refers to the reversible, distinct change in the position, intensity, and shape of the absorption or fluorescence band when a solute interacts with solvents of different polarities [13]. Positive and negative solvatochromism can be seen. In the case of the former, a bathochromic shift (red) is observed with increasing solvent polarity, while for the latter, the opposite effect occurs, i.e., a hypsochromic (blue) shift. 

The analysis of the environmental impact on the spectroscopic properties of compounds can be based, for example, on the solvent polarity scale as a function of *f*(ε,*n*). The *f*(ε,*n*) function, so-called orientational polarizability, allows for the estimation of changes in the dipole moments of molecules under the influence of excitation and is included, among others, in the Lippert–Mataga equation [14,15,16]. Other theories taking into account Onsager’s description of non-specific electrostatic solute–solvent interactions were proposed by McRae [17], Bakhshiev [18], and Kawski [19,20]. Despite different assumptions, the proposed methods come down to a similar equation in the form given below:

(1) $$\nu_{ab}-\nu_{fl}=m_{1}\cdot f(\varepsilon ,n)+const$$

where

(2) $$m_{1}=\frac{({\overset{\to }{\mu }}_{e}-{\overset{\to }{\mu }}_{g}{)}^{2}}{2\pi \varepsilon_{0}hca^{3}}$$

Here, *m*_1_ is the slope of the straight line (in cm^−1^), *μ_e_* and *μ_g_* are the dipole moments in the excited and ground states, respectively, *h* is Planck’s constant, *c* is the velocity of light in vacuum, and *a* is Onsager’s interaction radius of the solute (in Å). The function *f*(*ε,n*) is calculated based on the dielectric constant (*ε*) and refractive index (*n*) of solvents and is specific to individual theories [21].

Furthermore, a commonly used scale of the solvent polarity parameter is $E_{T}^{N}$ [22,23] proposed by Reichardt based on the polarity extremes of water and tetramethylsilane (TMS). According to this model, solvents can be classified as protic when $E_{T}^{N}$ ranges from 0.5 to 1, when they are dipolar non-hydrogen-donating ($E_{T}^{N}$ from 0.3 to 0.5), and apolar ($E_{T}^{N}$ from 0 to 0.3). Determining the value of the slope coefficient of the line for the relationship ${\Delta \nu }^{SS}=f\left(E_{T}^{N}\right)$ allows the change in the dipole moment between the excited and ground state to be estimated [24,25,26].

Solute–solvent interactions are generally divided into non-specific ones, caused by polarity and polarizability effects, and specific ones, which include hydrogen bonds [27]. Therefore, for a proper understanding and correct description of the dependence of the spectral and photophysical properties of the tested compounds on the properties of the solvent, its polarity/polarizability and the ability to form hydrogen bonds must be determined [28]. Solvents with the ability to form hydrogen bonds are classified as hydrogen bond acceptors or donors.

To analyze specific and non-specific solute–solvent interactions, the multi-parameter Catalán scale [29,30,31] and the Kamlet–Taft scale [32,33,34] can be used. In this case, the study of the interactions of the solute with solvents consists of multiple regression analysis based on four solvent parameters (polarizability (SP), dipolarity (SdP), acidity (SA) and basicity (SB)) given by Catalán in his work from 2009 [31] or the parameters (polarity/polarizability (π*), hydrogen bond donor (α) and hydrogen bond acceptor (β)) proposed by Taft and Kamlet [32,33,34].

The main aim of this work was to synthesize and determine the influence of the chemical structure of thirteen representatives of 5-(4-substituted-arylidene)-1,3-dimethylpyrimidine-2,4,6-trione on their photophysical properties. The designed dyes differ in the alkylamino group attached in the *para* position of the phenyl ring, which shows various electron-donating properties. We consider the influence of several factors, namely (a) the nature of the alkylamino group attached to the phenyl ring, (b) the geometry of this substituent, and (c) the polarity of the solvent on the photophysical properties of the tested compounds based on steady-state spectroscopic measurements and theoretical calculations.

## 2. Materials and Methods

All reagents and solvents were purchased from Merck Chemical Co. (Darmstadt, Germany). *N*-substituted aromatic aldehydes were synthesized in our laboratory according to the method described by Gawinecki et al. [35].

Melting points (uncorrected) were determined on a Boëthius apparatus (Vernon Hills, IL, USA).

The ^1^H (200 MHz) and ^13^C (50 MHz) NMR spectra were recorded on a Varian Gemini 200 NMR spectrometer (Billerica, MA, USA) in dimethylsulfoxide (DMSO-*d*_6_). The tetramethylsilane (TMS) was used as an internal standard. Chemical shifts are reported in ppm (δ). Coupling constants, *J*, are reported in Hz.

The IR spectra were collected on a Bruker Vector 22 FT-IR spectrophotometer (Karlsruhe, Germany). The spectra were recorded in the range 400–4500 cm^−1^ by applying the KBr pellet technique.

The Waters HPLC system (Framingham, MA, USA) equipped with a Waters 2489 UV-Vis detector (detection wavelength was 450 nm), Waters 1525 Binary HPLC Pump, and a Symmetry C18 column (3.5 μm, 4.6 × 75 mm) was used to perform HPLC analyses. Separation was conducted under isocratic conditions with a 0.8 mL/min flow rate, 25 °C, 10 μL injection volume, and HPLC-grade methanol as a mobile phase.

For thin-layer chromatography, aluminum oxide IB-F flexible sheets (thickness 0.2 mm) were purchased from J.T. Baker Chemical Co., Phillipsburg, NJ, USA. Chloroform was used as an eluent.

Absorption and emission spectra were recorded at room temperature on a Shimadzu UV-vis Multispec-1501 spectrophotometer (Kioto, Japan) and a Hitachi F-7100 spectrofluorimeter (Tokio, Japan), respectively, in the following solvents: toluene, 1,4-dioxane (1,4-Dx), toluene, diethyl ether (Et_2_O), ethyl acetate (EtOAc), tetrahydrofuran (THF), acetone (MeAc), acetonitrile (MeCN), *N*,*N*-dimethylformamide (DMF) and dimethylsulfoxide (DMSO). The final concentration of the dye in the solution was ca. 1.0 × 10^−5^ M for absorption and 1.0 × 10^−6^ M for fluorescence measurements, respectively. All solvents were of spectroscopic grade and were used without any additional purification. They were characterized by their static dielectric constant (*ε*) and refractive index (*n*) at 20°C. The fluorescence quantum yield of the dyes was determined by comparison with the emitted light to the fluorescence intensity of the standard, as described previously [36]. Coumarin 153 in ethanol (A ≈ 0.1 at 450 nm; *ϕ_ref_* = 0.38) was used as a reference [37].

The solvent effect on the spectral properties of tested dyes was analyzed based on the four-parameter solvent scale proposed by Catalán [31]. According to this model, the change in the properties (*y*), including the shift of the absorption (*ν*_Ab_), emission band (*ν*_Fl_) maxima, or Stokes shifts (Δ*ν*^SS^), is correlated with the solvent parameters (SP, SdP, SA, and SB), respectively, as shown in Equation (3).

(3) $$y=y_{0}+a_{\mathrm{SP}}\mathrm{SP}+b_{\mathrm{SdP}}\mathrm{SdP}+c_{\mathrm{SA}}\mathrm{SA}+d_{\mathrm{SB}}\mathrm{SB}$$

where *y*_0_ is the property of the substance of interest in the absence of a solvent, e.g., in the gas phase and *a*_SP_, *b*_SdP_, *c*_SA_, and *d*_SB_ are the corresponding coefficients of the solvent.

The estimate based on multilinear regression analysis Catalán coefficients for the spectra properties and the regression coefficient values (R-Square) are summarized in Table S2 in the ESI file.

### 2.1. Computational Details

To optimize the structures of the tested derivatives in the ground (S_g_), and excited (S_CT_) states, the density functional theory (DFT/PBE0) approach available in the Gaussian 09 program package [38] was used with the SCF = QC/XQC keyword in calculation settings. Hessian analysis confirmed that the obtained geometries are located at a minimum on the potential energy surface. The vertical absorption and emission maxima were estimated based on the time-dependent density functional theory (TDDFT) [39] by including the state-specific (SS) corrected linear response (cLR) approach [40]. Following previous research results [41,42,43,44,45,46], in this case, the PBE0 functional was used as it describes the electrical properties with the smallest error in relation to the experimental values. The dipole moment and polarity of the charge-transfer state (CT, *μ*_CT_) were evaluated by the numerical differentiation of the excitation energies (*E*) as follows:

(4) $$∆\mu_{g-CT}^{i}=\frac{E_{CT}\left(+F^{i}\right)-E_{CT}\left(-F^{i}\right)}{-2F^{i}}-\frac{E_{g}\left(+F^{i}\right)-E_{g}\left(-F^{i}\right)}{-2F^{i}}$$

where *i* stands for the Cartesian component of the dipole moment difference and *F* is an electric field of 0.001 a.u. strength.

The density differences were obtained at the PBE0/6-311++G(d,p) level and are represented by a contour threshold of 0.02 a.u. The charge transfer parameters, namely the charge-transfer distance (*D*_CT_) and the amount of transferred charge (*q*_CT_), were determined following Le Bahers’ procedure [47]. Solvent influences on the presented parameters were determined based on the Integral Equation Formalism for the Polarizable Continuum Model (IEF–PCM) [48,49]. All calculations were performed at the 6-311++G(d,p) base level.

### 2.2. Synthesis Route and Basic Characterization

For the synthesis of merocyanine-type dyes, namely 5-(4-substituted-arylidene)-1,3-dimethylpyrimidine-2,4,6-triones, the procedure described by F. Würthner and S. Yao [50] and Hong et al. [51] based on Knoevenagel condensation was adapted. Alkylaminobenzaldehyde (0.01 mol), 1,3-dimethylbarbituric acid (0.01 mol), and acetic anhydride (Ac_2_O; 5 mL) were mixed and heated at 90 °C for 3 h. After cooling to RT, the crude dye was filtered off, washed with acetic anhydride, 2-propanol, and hexane, and dried. 

#### 2.2.1. 5-[4-(*N*,*N*-Dimethylamino)benzylidene]-1,3-dimethylpyrimidine-2,4,6(1*H*,3*H*,5*H*)-trion (**1**)

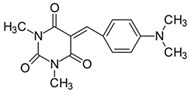

The dye was prepared using 1,3-dimethylbarbituric acid (1.56 g, 0.01 mol), 4-(*N*,*N*-dimethylamino)benzaldehyde (1.48 g, 0.01 mol), and acetic anhydride (5 mL). Red and pink needles were obtained; yield 75.8%, mp 241.5 °C (lit. 224–226 °C [52], 240–242 °C [53]), R_f_ 0.46.

^1^H NMR (200 MHz, DMSO-*d*_6_) σ (ppm): 3.126 (s, 6H, NCH_3_), 3.214 (s, 6H, N(CH_3_)_2_), 6.776–6.823 (d, *J* = 9.4 Hz, 2H, Ar), 8.222 (s, 1H, -CH=), 8.387–8.433 (d, *J* = 9.2 Hz, 2H, Ar).

^13^C NMR (50 MHz, DMSO-*d*_6_) δ (ppm): 27.903, 28.531 (NCH_3_ in barbituric acid) 39.691 (CH_3_, NCH_3_), 111.128, 139.000 (CH, Ar), 156.195 (-CH=), 109.253, 119.939, 145.900, 154.129, 161.065, 163.113 (C).

IR (KBr): 2922 (-CH), 1713, 1660, 1608 (C=O), 1534, 1506 (C=C), 1196, 1141, 1085 (C-N), 831, 786, 752 (=CH).

#### 2.2.2. 5-[4-(*N*,*N*-Diethylamino)benzylidene]-1,3-dimethylpyrimidine-2,4,6(1*H*,3*H*,5*H*)-trion (**2**)

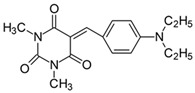

The dye was prepared using 1,3-dimethylbarbituric acid (1.56 g, 0.01 mol), 4-(*N*,*N*-diethylamino)enzaldehyde (1.77 g, 0.01 mol), and acetic anhydride (5 mL). Dark-brown crystals were obtained; yield 81.2%, mp 190 °C, R_f_ 0.64.

^1^H NMR (200 MHz, DMSO-*d*_6_) σ (ppm): 1.112–1.181 (t, 6H, -CH_3_), 3.200 (s, 6H, NCH_3_), 3.450–3.555 (m, 4H, NCH_2_-), 6.758–6.803 (d, *J* = 9 Hz, 2H, Ar), 8.184 (s, 1H, -CH=), 8.367–8.413 (d, *J* = 9.2 Hz, 2H, Ar).

^13^C NMR (50 MHz, DMSO-*d*_6_) δ (ppm): 12.529 (CH_3_), 27.867, 28.504 (NCH_3_ in barbituric acid), 44.270 (NCH_2_-), 110.846, 139.473 (CH, Ar), 155.986 (-CH=), 108.652, 119.612, 151.125, 152.217, 161.056, 163.140 (C).

IR (KBr): 2970 (-CH), 1711, 1655, 1607 (C=O), 1536, 1501 (C=C), 1207, 1159, 1078 (C-N), 820, 787, 756 (=CH).

#### 2.2.3. 5-[4-(Ethylmethylamino)benzylidene]-1,3-dimethylpyrimidine-2,4,6(1*H*,3*H*,5*H*)-trion (**3**)

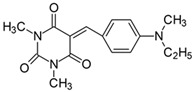

The dye was prepared using 1,3-dimethylbarbituric acid (1.56 g, 0.01 mol), 4-[*N*,*N*-ethyl(methyl)amino]benzaldehyde (1.63 g, 0.01 mol), and acetic anhydride (5 mL). Bright orange needles were obtained; yield 91.6%, mp 168 °C, R_f_ 0.45.

^1^H NMR (200 MHz, DMSO-*d*_6_) σ (ppm): 1.099–1.169 (t, 3H, -CH_3_), 3.092 (s, 3H, NCH_3_), 3.350 (s, 6H, NCH_3_) 3.516–3.621 (m, 2H, NCH_2_-), 6.793–6.838 (d, *J* = 9 Hz, 2H, Ar), 8.215 (s, 1H, -CH=) 8.389–8.435 (d, *J* = 9.2 Hz, 2H, Ar).

^13^C NMR (50 MHz, DMSO-*d*_6_) δ (ppm): 11.664 (CH_3_), 27.894, 28.522 (NCH_3_ in barbituric acid), 37.397 (NCH_3_) 46.145 (NCH_2_-) 111.028, 139.264 (CH, Ar), 156.104 (-CH=), 108.962, 119.839, 151.125, 153.164, 161.047, 163.122 (C).

IR (KBr): 2977 (-CH), 1712, 1654, 1608 (C=O), 1536, 1506 (C=C), 1198, 1162, 1083 (C-N), 818, 793, 786, 756 (=CH).

#### 2.2.4. 5-[4-(*N*,*N*-Dimethylamino)-2-methylbenzylidene]-1,3-dimethylpyrimidine-2,4,6(1*H*,3*H*,5*H*)-trion (**4**)

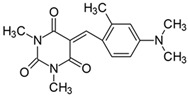

The dye was prepared using 1,3-dimethylbarbituric acid (1.56 g, 0.01 mol), 4-(*N*,*N*-dimethylamino-2-methyl)benzaldehyde (1.63 g, 0.01 mol), and acetic anhydride (5 mL). Orange-red petals were obtained; yield 93.1%, mp 210 °C, R_f_ 0.59.

^1^H NMR (200 MHz, DMSO-*d*_6_) σ (ppm): 2.425 (s, 3H, -CH_3_), 3.104 (s, 6H, NCH_3_), 3.192 (s, 3H, NCH_3_), 3.215 (s, 3H, NCH_3_), 6.594–6.667 (m, 2H, Ar), 8.577 (s, 1H, -CH=), 8.677–8.722 (d, *J* = 9 Hz, 1H, Ar).

^13^C NMR (50 MHz, DMSO-*d*_6_) δ (ppm): 20.867 (CH_3_), 27.912, 28.467 (NCH_3_ in barbituric acid), 39.636 (NCH_3_), 108.707, 112.612, 136.206 (CH, Ar), 152.936 (-CH=), 109.289, 119.065, 145.772, 151.161, 154.101, 160.783, 163.204 (C).

IR (KBr): 2947 (-CH), 1707, 1652, 1611 (C=O), 1533, 1507 (C=C), 1303, 1212, 1077 (C-N), 837, 783, 755 (=CH).

#### 2.2.5. 5-[4-(*N*,*N*-Dibutylamino)benzylidene]-1,3-dimethylpyrimidine-2,4,6(1*H*,3*H*,5*H*)-trion (**5**)

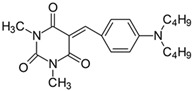

The dye was prepared using 1,3-dimethylbarbituric acid (1.56 g, 0.01 mol), 4-(*N*,*N*-dibutylamino)benzaldehyde (2.32 g, 0.01 mol), and acetic anhydride (5 mL). Long, thin light-orange needles were obtained; yield 88.2%, mp 142.8 °C, R_f_ 0.76.

^1^H NMR (200 MHz, DMSO-*d*_6_) σ (ppm): 0.892–0.964 (t, 6H, -CH_3_), 1.293–1.366 (m, 4H, -CH_2_-), 1.526–1.561 (m, 4H, -CH_2_-), 3.218 (s, 6H, NCH_3_), 3.449–3.483 (t, 4H, NCH_2_-), 6.754–6.800 (d, *J* = 9.2 Hz, 2H, Ar), 8.197 (s, 1H, -CH=), 8.371–8.415 (d, *J* = 8.8 Hz, 2H, Ar).

^13^C NMR (50 MHz, DMSO-*d*_6_) δ (ppm): 13.849 (-CH_3_), 27.885, 28.522 (NCH_3_ in barbituric acid), 19.583, 29.132 (-CH_2_-), 50.050 (NCH_2_-), 111.010, 139.355, (CH, Ar) 155.958 (-CH=), 108.698, 119.630, 151.152, 152.645, 161.092, 163.167 (C).

IR (KBr): 2955 (-CH), 1717, 1660, 1607 (C=O), 1540, 1505 (C=C), 1202, 1163, 1086 (C-N), 817, 787, 757 (=CH).

#### 2.2.6. 5-[4-(**N**-Pyrrolidino)benzylidene]-1,3-dimethylpyrimidine-2,4,6(1*H*,3*H*,5*H*)-trion (**6**)

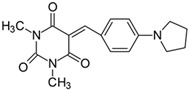

The dye was prepared using 1,3-dimethylbarbituric acid (1.56 g, 0.01 mol), 4-(1-pyrrolidino)benzaldehyde (1.75 g, 0.01 mol), and acetic anhydride (5 mL). Dark-red flakes were obtained; yield 66.8%, mp 230 °C, R_f_ 0.56.

^1^H NMR (200 MHz, DMSO-*d*_6_) σ (ppm): 1.998–2.029 (m, 4H, -CH_2_-), 3.222 (s, 6H, NCH_3_), 3.419–3.450 (m, 4H, NCH_2_-), 6.651–6.695 (d, *J* = 8.8 Hz, 2H, Ar), 8.226 (s, 1H, -CH=), 8.410–8.445 (d, *J* = 7 Hz, 2H, Ar).

^13^C NMR (50 MHz, DMSO-*d*_6_) δ (ppm): 27.912, 28.531 (CH_3_ in barbituric acid), 24.872 (-CH_2_-), 47.692 (NCH_2_-), 111.711, 139.319 (CH, Ar), 156.277 (-CH=), 108.597, 119.894, 150.900, 151.689, 161.110, 163.177 (C).

IR (KBr): 2958 (-CH), 1714, 1654, 1608 (C=O), 1533, 1506 (C=C), 1192, 1156, 1078 (C-N), 799, 784, 754 (=CH).

#### 2.2.7. 5-[4-(*N*-Piperidinyl)benzylidene]-1,3-dimethylpyrimidine-2,4,6(1*H*,3*H*,5*H*)-trion (**7**)

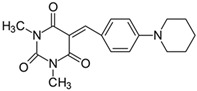

The dye was prepared using 1,3-dimethylbarbituric acid (1.56 g, 0.01 mol), 4-(1-piperidinyl)benzaldehyde (1.85 g, 0.01 mol), and acetic anhydride (5 mL). Long, irregular, brown needles were obtained; yield 35.1%, mp 197.4 °C, R_f_ 0.8.

^1^H NMR (200 MHz, DMSO-*d*_6_) σ (ppm): 1.616 (m, 6H, -CH_2_-), 3.211 (s, 6H, NCH_3_), 3.553 (m, 4H, NCH_2_-), 6.966–7.011 (d, *J* = 9 Hz, 2H, Ar), 8.169 (s, 1H, -CH=), 8.350–8.394 (d, *J* = 8.8 Hz, 2H, Ar).

^13^C NMR (50 MHz, DMSO-*d*_6_) δ (ppm): 27.921, 28.549 (CH_3_ in barbituric acid), 24.007, 25.236 (-CH_2_-), 47.374 (NCH_2_-), 112.157, 139.173 (CH, Ar), 155.813 (-CH=), 109.617, 120.376, 151.125, 153.865, 161.019, 163.076 (C).

IR (KBr): 2942 (-CH), 1710, 1653, 1607 (C=O), 1540, 1506 (C=C), 1202, 1165, 1084 (C-N), 814, 788, 755 (=CH).

#### 2.2.8. 5-[4-(4-Morpholinyl)benzylidene]-1,3-dimethylpyrimidine-2,4,6(1*H*,3*H*,5*H*)-trion (**8**)

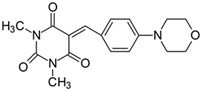

The dye was prepared using 1,3-dimethylbarbituric acid (1.56 g, 0.01 mol), 4-(4-morpholinyl)benzaldehyde (1.91 g, 0.01 mol), and acetic anhydride (5 mL). Dark-red needles were obtained; yield 75.2%, mp 265 °C, R_f_ 0.58.

^1^H NMR (200 MHz, DMSO-*d*_6_) σ (ppm): 3.223 (s, 6H, NCH_3_), 3.449–3.498 (t, 4H, -CH_2_-), 3.713–3.759 (t, 4H, NCH_2_-), 7.005–7.049 (d, *J* = 8.8 Hz, 2H, Ar), 8.248 (s, 1H, -CH=), 8.366–8.411 (d, *J* = 9 Hz, 2H, Ar).

^13^C NMR (100 MHz, DMSO-*d*_6_) δ (ppm): 27.971, 28.593 (CH_3_ in barbituric acid), 46.207 (NCH_2_-), 65.807 (OCH_2_-), 112.426, 138.600 (CH, Ar), 156.070 (-CH=), 111.305, 121.686, 151.238, 154.292, 161.130, 163.098 (C).

IR (KBr): 2970 (-CH), 1711, 1653, 1609 (C=O), 1545, 1514 (C=C), 1242, 1208, 1123, 1084 (C-N), 836, 787, 754 (=CH).

#### 2.2.9. 1,3-Dimethyl-5-[(1-methyl-2,3-dihydro-1*H*-indolyl)methylidene]pyrimidine-2,4,6(1*H*,3*H*,5*H*)-trion (**9**)

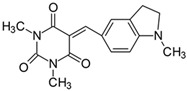

The dye was prepared using 1,3-dimethylbarbituric acid (1.56 g, 0.01 mol), 4-(1-methyl-2,3-dihydro-1*H*-indolyl)benzaldehyde (1.61 g, 0.01 mol), and acetic anhydride (5 mL). Dark-red crystals were obtained; yield 80.1%, mp 226 °C, R_f_ 0.56.

^1^H NMR (200 MHz, DMSO-*d*_6_) σ (ppm): 2.991 (s, 3H, NCH_3_), 3.045–3.086 (t, 2H, -CH_2_-), 3.215 (s, 6H, NCH_3_), 3.662–3.745 (t, 2H, NCH_2_-), 6.551–6.594 (d, *J* = 8.6 Hz, 1H, Ar), 8.079–8.124 (d, *J* = 9 Hz, 1H, Ar), 8.167 (s, 1H, -CH=), 8.480 (s, 1H, Ar).

^13^C NMR (50 MHz, DMSO-d_6_) δ (ppm): 27.894, 28.504 (CH_3_ in barbituric acid), 33.064 (NCH_3_), 26.392 (-CH_2_-), 53.991 (NCH_2_), 104.838, 131.090, 142.486 (CH, Ar) 156.031 (-CH=), 121.177, 127.595, 130.007, 158.015, 163.277, 165.534, 177.204 (C).

IR (KBr): 2952 (-CH), 1708, 1652, 1616 (C=O), 1506, 1462 (C=C), 1294, 1160, 1071 (C-N), 802, 787, 757 (=CH).

#### 2.2.10. 1,3-Dimethyl-5-[(1-methyl-1,2,3,4-tetrahydroquinolinyl)methylidene]pyrimidine-2,4,6(1*H*,3*H*,5*H*)-trion (**10**)

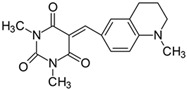

The dye was prepared using 1,3-dimethylbarbituric acid (1.56 g, 0.01 mol), 4-(1-methyl-1,2,3,4-tetrahydroquinoline)benzaldehyde (1.75 g, 0.01 mol), and acetic anhydride (5 mL). Dark-red crystals were obtained; yield 73.5%, mp 228.4 °C, R_f_ 0.41.

^1^H NMR (200 MHz, DMSO-*d*_6_) σ (ppm): 1.894 (m, 2H, -CH_2_-), 2.717 (t, 2H, -CH_2_-), 3.459 (t, 2H, NCH_2_-), 3.079 (s, 3H, NCH_3_), 3.216 (s, 6H, NCH_3_), 6.677–6.721 (d, *J* = 8.8 Hz, 1H, Ar), 8.165 (s, 1H, -CH=), 8.205 (s, 1H, Ar), 8.259–8.304 (d, *J* = 9 Hz, 1H, Ar).

^13^C NMR (50 MHz, DMSO-*d*_6_) δ (ppm): 27.906, 28.515 (CH_3_ in barbituric acid), 38.597 (NCH_3_), 20.893, 27.043 (-CH_2_-), 50.880 (NCH_2_), 110.034, 137.091, 151.722 (CH, Ar) 156.196 (-CH=), 108.179, 119.996, 121.438, 138.814, 151.302, 161.221, 163.363 (C).

IR (KBr): 2942 (-CH), 1709, 1652, 1609 (C=O), 1503, 1477 (C=C), 1205, 1160, 1076 (C-N), 803, 786, 756 (=CH).

#### 2.2.11. 1,3-Dimethyl-5-[(juloidine)methylidene]pyrimidine-2,4,6(1*H*,3*H*,5*H*)-trion (**11**)

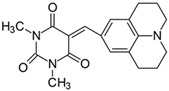

The dye was prepared using 1,3-dimethylbarbituric acid (1.56 g, 0.01 mol), 2,3,6,7-tetrahydro-1*H*,5*H*-pyrido [3,2,1-ij]quinoline-9-carbaldehyde (2.01 g, 0.01 mol), and acetic anhydride (5 mL). Bright maroon needles were obtained; yield 76.1%, mp 223.6 °C (lit. 197–198 °C [54]), R_f_ 0.32.

^1^H NMR (200 MHz, DMSO-*d*_6_) σ (ppm): 1.883 (m, 4H, -CH_2_-), 2.700 (t, 4H, -CH_2_-), 3.394 (t, 4H, NCH_2_-), 3.210 (s, 6H, NCH_3_), 8.079 (s, 3H, Ar, -CH=).

^13^C NMR (50 MHz, DMSO-*d*_6_) δ (ppm): 27.888, 28.480 (CH_3_ in barbituric acid), 20.550, 26.976 (-CH_2_-), 49.829 (NCH_2_), 120.223, 136.8464 (CH, Ar) 155.866 (-CH=), 106.965, 119.3338, 149.066, 151.333, 161.221, 163.457 (C).

IR (KBr): 2950 (-CH), 1708, 1653 (C=O), 1499 (C=C), 1215, 1185, 1161, 1099 (C-N), 785, 756 (=CH).

#### 2.2.12. 1,3-Dimethyl-5-[4-(*N*,*N*-diphenylamino)benzylidene]pyrimidine-2,4,6(1*H*,3*H*,5*H*)-trion (**12**)

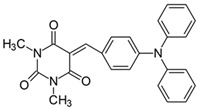

The dye was prepared using 1,3-dimethylbarbituric acid (1.56 g, 0.01 mol), 4-(*N*,*N-*diphenylamino)benzaldehyde (2.73 g, 0.01 mol), and acetic anhydride (5 mL). Spherical, dark brown grains were obtained; yield 46.1%, mp 193 °C (lit. 190 °C [55]), R_f_ 0.55.

^1^H NMR (200 MHz, DMSO-*d*_6_) σ (ppm): 3.192 (s, 3H, NCH_3_) 3.218 (s, 3H, NCH_3_), 6.758–6.802 (d, *J* = 8.8 Hz, 2H, Ar), 7.270 (m, 5H, Ar), 7.448 (m, 5H, Ar), 8.230 (s, 1H, -CH=), 8.238–8.285 (d, *J* = 9.4 Hz, 2H, Ar).

^13^C NMR (50 MHz, DMSO-*d*_6_) δ (ppm): 28.012, 28.640 (CH_3_ in barbituric acid), 113.276, 116.744, 123.853, 137.471, 144.899 (CH, Ar) 155.394 (-CH=), 125.938, 126.348, 126.630, 130.025, 151.034, 152.117, 160.764, 162.703 (C).

IR (KBr): 2953 (-CH), 1729, 1662, (C=O), 1589 (C=C), 1193, 1156, 1086 (C-N), 792, 755, 700 (=CH).

#### 2.2.13. 5-[4-(*N*,*N*-Dimethylamino)-2,6-dimethylbenzylidene]-1,3-dimethylpyrimidine-2,4,6(1*H*,3*H*,5*H*)-trion (**13**)

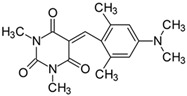

The dye was prepared using 1,3-dimethylbarbituric acid (1.56 g, 0.01 mol), 4-(*N*,*N*-dimethylamino-2,6-dimethyl)benzaldehyde (1.77 g, 0.01 mol), and acetic anhydride (5 mL). Orange-red petals were obtained; yield 64.7%, mp 192 °C, R_f_ 0.59.

^1^H NMR (200 MHz, DMSO-*d*_6_) σ (ppm): 2.134 (s, 6H, CH_3_), 2.972 (s, 6H, NCH_3_), 3.128 (s, 3H, NCH_3_), 3.231 (s, 3H, NCH_3_), 6.446 (s, 2H, Ar), 8.431 (s, 1H, -CH=).

^13^C NMR (50 MHz, DMSO-*d*_6_) δ (ppm): 20.940 (CH_3_), 27.858, 28.367 (CH_3_ in barbituric acid), 39.691 (NCH_3_), 110.755 (CH, Ar), 155.330 (-CH=), 117.727, 122.042, 139.410, 151.107, 151.207, 159.663, 161.911 (C).

IR (KBr): 2923, (-CH), 1724, 1667, 1611 (C=O), 1545, 1512 (C=C), 1156, 1090 (C-N), 784, 756 (=CH).

## 3. Results

### 3.1. Synthesis and Design Strategy

**Scheme 1:**
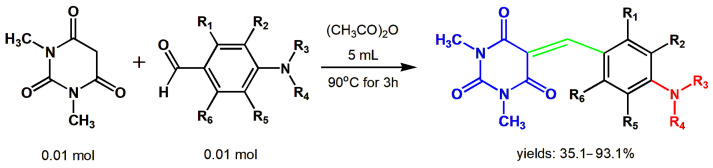
Schematic presentation of the synthesis of merocyanine-type dyes tested.

Merocyanine-type dyes were prepared in a one-step synthesis, as outlined in Scheme 1. The 1,3-dimethylbarbituric acid was condensed with an appropriate aldehyde to afford the corresponding dyes with good yields (35.1–93.1%). Their chemical structure was verified by IR, ^1^H, and ^13^C NMR spectroscopy. The chemical shifts, multiplicity, and integration of the relevant groups of protons in the ^1^H NMR, as well as the signals in ^13^C NMR and IR spectra, are consistent with the dye structures. For example, the disappearance of the peak at 10 ppm (190 ppm in ^13^C NMR) assigned to the CHO group and the appearance of a new peak at 8.2 ppm (156 ppm in ^13^C NMR), attributed to the formation of the methine bond, confirmed the formation of the desired product. The purity of the compounds was checked by HPLC and thin-layer chromatography. The HPLC chromatograms, ^1^H and ^13^C NMR, and IR spectra are shown as images before Table S1 in the Supplementary Materials.

The tested merocyanine-type dyes represent a typical push–pull chemical structure with electron-withdrawing and electron-releasing groups on opposite sides of the methine bridge. The third-order amine group in the *para* position of the phenyl ring creates terminal moieties rich with free electrons that add an electron density into a conjugated π system. The opposite effect reveals the barbituric acid ring, which, especially with its oxygen atoms, serves as the strong electron-withdrawing moiety. The methine linker, consisting of a carbon atom bound by one single bond to the phenyl ring and one double bond to the barbituric acid unit, is responsible for the flexibility of the dyes. Thus, any kind of conformational transformation, e.g., *trans-cis* isomerization with a double bond or the molecular rotation of a single bond, may occur. This may be prevented by appropriate substituents. However, negligible photobleaching occurred upon irradiation with the 457 nm laser light of the dyes in ethyl acetate during periods of time longer than half an hour (see Figure S1 in ESI), indicating the good photostability of the dyes in solution and a lack of trans-cis photoisomerization.

### 3.2. Structure Effect on Spectroscopic Properties

**Figure 1:**
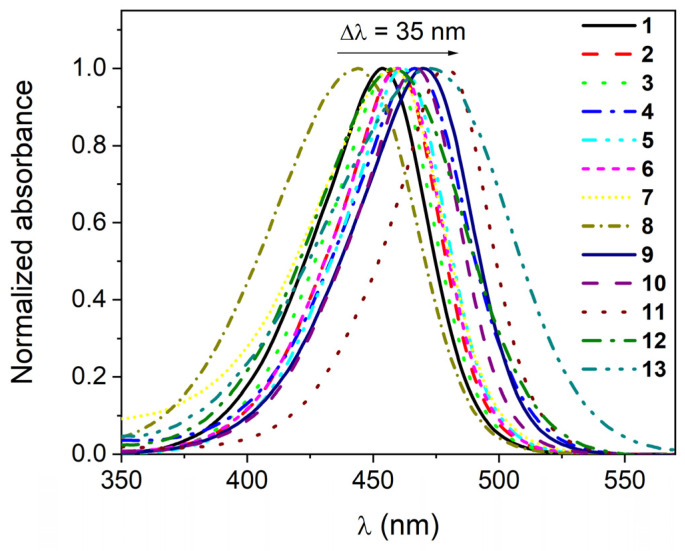
Normalized electronic absorption spectra of dyes **1**–**13** in THF.

The electronic absorption spectra, illustrated in Figure 1, are characterized by the presence of one main band. Its wavelength at maximum ranges from 450 nm to 490 nm, depending on the molecular structure of the dye. This band has a high molar absorption coefficient and corresponds to the π→π* transition with a charge transfer (CT) character. The most significant linear properties of the tested compounds in three selected solvents of different polarities are listed in Table 1, and the data in all the solvents used are collected in Table S1 in the ESI file.

To evaluate the effect of the substituent at 4 position in phenyl moiety on the linear optical properties, -NMe_2_ was used as a benchmark tertiary amine group. The dyes under study were selected in a way that allowed changes in both the mobility and the electron-releasing properties of the dialkylamino group. The variation in the structure of the rotating part of the molecules was aimed at forcing a pre-twist or preventing the rotation of the dialkylamino group. These can be achieved by the control of the freedom of all possible rotations around the bonds.

From the data listed in Table 1 and Table S1 in the ESI file and displayed in Figure 1, one notices that the nature of the dialkylamino group induces shifts in the absorption maxima of ca. 35 nm. This allowed us to state that the variation in the band position is quite large, especially as the amino groups differ only by their exact substitutions and geometries. For example, an increase in the number of methylene units attached to the nitrogen atom gradually shifts the absorption to the red (compound **1** vs. **2**, **3,** and **5**). The introduction of the NBu_2_ group (dye **5**) in place of the NMe_2_ substituent (dye **1**) shifts the absorption band to the red by approximately 7–9 nm. A similar effect is observed after introducing methyl groups in the meta position in relation to the *N,N*-dimethylamino group (compounds **4** and **13**). Simultaneously, a strong hypochromic effect is observed, e.g., molar absorption coefficients of the considered dyes decrease from 7.31 × 10^4^ M^−1^cm^−1^ for the parent compound (**1**) to 5.34 × 10^4^ M^−1^cm^−1^ and 1.79 × 10^4^ M^−1^cm^−1^ for **4** and **13** in DMSO, respectively. This effect is connected with a steric interaction separating the NMe_2_ group from the electron-accepting part of the molecule [56], which leads to the pre-twisting of the dye even in the ground state. This is a result of the interaction of the methyl group with hydrogen derived from the methine group. Similar results were described by Gawinecki et al. for the group of styrylpyridinium dyes [57] and *para*-substituted benzaldoximes [58]. 

Absorption at *λ_max_* was also found to progressively shift to a longer wavelength upon the stiffening of the dialkylamino group by bridging with a phenyl ring. The symmetrical stiffening of the amino group, like in compound **11,** shifts the absorption to the red by more than 20 nm. However, based on our previous observations on difluoroboranes containing a dialkylamino group of different structure and geometry, it should not reach more than 7–9 nm [59]. Therefore, it can be assumed that the elimination of the free rotation of the amino group results in more efficient charge transfer (CT), producing the most bathochromically shifted absorption since the planar conformation of the substituent in **11** increases the probability of radiative transitions with respect to other compounds. Partial stiffening of the amino group by bridging on one side, as in compounds 9 and 10, also redshifts the absorption but with less variability. Thus, increasing the length of methylene bridges from two to three carbon atoms changes the maximum absorption wavelength of 16 nm and 13 nm for **9** and **10**, respectively, compared to **1**, of which ~3 nm comes from the methyl group at the *meta* position of the aromatic ring [59]. The discrepancy in the shifts arises presumably from the fact that the six membered rings can be inverted much more easily due to thermal movements [59].

A blue shift in the CT absorption band was observed for the pyrrolidine, piperidine, and morpholine derivatives. However, for compounds **6** and **7,** the absorption maximum was red-shifted relative to **1**. All these heterocycles may exist as two ground state conformers, i.e., chair and boat, taking both the axial and equatorial forms. The conformational transformations occurring by pseudorotation or by the interconversion through the planar form (depending on the energy barrier) may lead to the coplanar conformation of the alkylamino group with respect to the benzene ring, which decreases the probability of radiative transitions. Thus, the flexibility of the six-membered ring, including boat-to-chair or axial-to-equatorial conformational changes, forces the amino moiety to deviate from its relaxed geometry, which may shift absorption to the blue [60,61,62].

Similar conclusions can be drawn with respect to the fluorescence band position. Firstly, the dyes have broad structureless emission spectra with a maximum of about 511–667 nm. The changes in the fluorescence maxima caused by an alkyl group are ∼25 nm in THF. For example, the range of change in the fluorescence band reaches 22 nm between dyes **1** and **11** in THF. The strongest shifts of +121 nm were induced by transitioning from an NMe_2_ substituent to an NPh_2_ auxochrome (**1** vs. **12**). A similar value of Δ*λ_max_* for fluorescence and absorption indicates that the geometry of the different members of the dialkylamino derivatives of merocyanine dyes do not change significantly upon excitation. However, the substitution at the *para* position with NPh_2_ instead of NMe_2_ induces a stronger bathochromic displacement of the fluorescence than absorption, indicating that the dye **12** geometry changes more in the excited state. These results are also reflected in the values of the full width at half-maximum (FWHM) and Stokes shift. The stiffening of the amino group by aliphatic bridges leads to a decrease in FWHM and Stokes shift from 2204 cm^−1^ and 2930 cm^−1^ to 1772 cm^−1^ and 2494 cm^−1^, respectively, for **1** and **11**. Since the flexibility of the amino group is blocked in **11**, it can be stated that rotations around the C−N bond in **1** result in the more coplanar arrangement of the NMe_2_ group, and the aromatic ring in the excited state leads to the twisted intramolecular charge transfer (TICT) or more likely to conformational relaxed ICT (CRICT) geometries [63,64,65]. Meanwhile, the fluorescence is more effective for compounds with a flattened structure, as shown by the comparison of the fluorescence quantum yields; thus, the observed emissions all come rather from planar intramolecular charge transfer (PICT) [66] geometries in this series. It is noted that the fluorescence quantum yield for dyes **1**–**11** and **13** is very small and oscillates around 0.05%, whereas it is one order of magnitude higher for compound **12** bearing the *N*,*N*-diphenylamino group (Table 2). The low fluorescence quantum yields can be attributed, at least in part, to the photoinduced intramolecular charge transfer (ICT) interaction, which contributes effectively to fluorescence quenching [67].

In general, the shift of the absorption and fluorescence bands towards longer wavelengths resulting from the change in substituent in the benzene ring (electron donor group) is consistent with the intramolecular charge transfer (ICT) nature of the corresponding electronic transition. This indicates an electronic interaction between the alkylamino group with variable electron-donating properties and the barbiturate ring, which is an electron acceptor, through the methine bridge. The stiffening and flattening of the amino group in relation to the benzene ring promotes the delocalization of the electron lone pair located at amino nitrogen towards the rest of the molecule [56,57,58]. 

### 3.3. Solvent Effect on Spectroscopic Properties

As mentioned above, the spectral properties of the merocyanine-type dyes are related to the nature of the substituent attached to the phenyl ring. The change in both exact substitutions and geometries of the tertiary amino group can tune the absorption and fluorescence maximum to the red by more than 20 nm. It is also well known that such compounds should be sensitive to the polarity of the environment due to the push–pull structure, which ensures that the charge is transferred from the donor group to the acceptor upon light absorption [67]. Thus, the solvent effects on the spectral shifts of the merocyanine dyes containing the dialkylamino group in phenyl moiety were analyzed as well. 

The absorption spectra of tested dyes were examined in nine solvents of different polarities. For dyes with a dimethylamino substituent (**1**), the spectral shift of the absorption maxima between the highest and lowest value (Δ*λ_max_*), due to change in solvent, was within 20 nm, whereas dye **11**, possessing a stiffened alkylamino group, showed a shift of about 25 nm. In general, upon increasing the solvent polarity, the CT absorption band was red-shifted, which allowed us to conclude that the dipole moments of the excited states reached directly after excitation were rather large [68,69,70].

**Figure 2:**
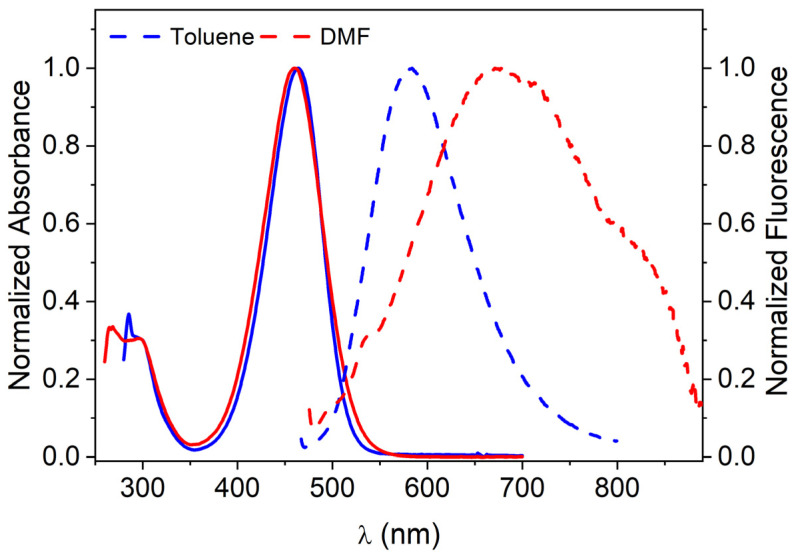
Normalized electronic absorption (solid line) and fluorescence (dash line) spectra of **12** in toluene and DMF. Excitation wavelengths 450 nm and 460 nm, respectively.

Fluorescence spectra were recorded in the same solvents as the electronic absorption spectra. Figure 2 displays an example of the solvent effect on the absorption and emission spectra of the **12** dyes.

As for absorption spectra, the increase in solvent polarity is responsible for shifting the emission band to the red. For example, the change in the solvent from Et_2_O to DMSO in the case of **1** changed the maximum fluorescence wavelength from 511 nm to 543 nm or from 588 nm to 651 for compound **12**. The large redshifts of the emission bands indicate the greater stabilization of the excited singlet state in polar solvents. 

The Stokes’ shifts in a series slightly vary from solvent to solvent and with the change in substituent in phenyl moiety (Table 1). The values of Δ*ν*^SS^ lie in the range of 2400–3300 cm^−1^. Only in the case of **12** are higher values of Stokes’ shifts achieved. From low to more polar solvents, this parameter increases from 4130 cm^−1^ to ca. 6400 cm^−1^. The relatively large Stokes’ shifts exhibited are common to π-conjugated donor–acceptor compounds and are attributed to the charge-transfer characteristics [63,68,71,72,73,74,75]. 

The solvatochromism of the tested dyes was studied on the basis of the SP, SdP, SA, and SB solvent scales developed by Catalán[31]. Fitting the absorption and emission data as well as the Stokes shift values of dyes **1–13** to Equation (3), satisfactory resultant fits were obtained. The exemplary results of the multiple linear regression analysis performed for dye **1** are presented below, whereas the estimated coefficients (*y*_0_, *a*_SP_, *b*_SdP_, *c*_SA_, and *d*_SB_), their standard errors, and correlation coefficients (*R^2^*) for all tested dyes are collected in Table S2 in the ESI file.

$$\nu_{Ab}=\left(24,068\pm 155\right)-\left(2181\pm 200\right)SP-\left(798\pm 69\right)SdP-\left(1491\pm 779\right)SA-\left(9\pm 78\right)SB\;with\;n=9\;and\;R^{2}=0.989$$

$$\nu_{Fl}=\left(21,269\pm 155\right)-\left(1943\pm 200\right)SP-\left(1295\pm 89\right)SdP-\left(1497\pm 1006\right)SA-\left(2\pm 100\right)SBwith\;n=9\;and\;R^{2}=0.991$$

$${\Delta \nu }^{SS}=\left(2801\pm 250\right)-\left(238\pm 250\right)SP+\left(497\pm 86\right)SdP+\left(6\pm 974\right)SA-\left(7\pm 97\right)SBwith\;n=9\;and\;R^{2}=0.920$$

Based on the solvent coefficients, one can state that the merocyanine dyes show a positive solvatochromism dominated by non-specific interactions. The absorption band position is redshifted, especially due to the susceptibility to solvent polarization, while the solvent dipolarity, with a significant share of the solvent polarizability, largely affects the fluorescence maximum. The positive solvatochromism is confirmed for both the absorption and fluorescence spectra of the tested dyes by negative coefficient values of the SP, SdP, and SA terms in Equation (3). A weak blue shift (positive *c*_SA_) is observed with increasing solvent acidity (SA) for compound **6**; however, this coefficient is subjected to a large error. A similar conclusion can be drawn for compound **7** with respect to fluorescence maxima. A more complex situation is observed for **12**. A blue shift with increasing solvent dipolarity (SdP) for absorption and polarizability (SP) and acidity (SA) for fluorescence occurs, as indicated by the positive signs of *a*_SP_, *b*_SdP_, and *c*_SA_, but the values are subjected to large errors. The shift between absorption and fluorescence *λ_max_* is also controlled by the overall solvent effect. As the obtained data show, the SdP scale provides an adequate description of the Stokes shift with some SP involvement. The *b*_SdP_ and *a*_SP_ coefficients have higher values with a relatively low error.

**Figure 3:**
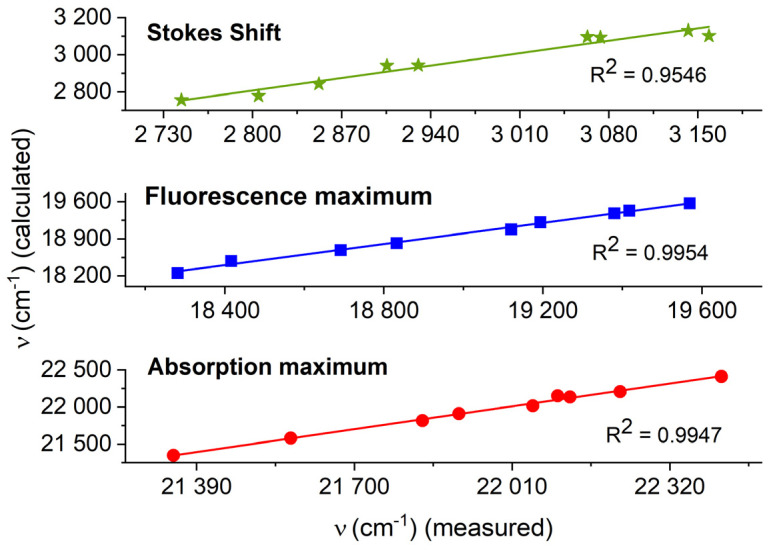
Correlation between calculated and measured wavenumber values for **one** in nine solvents of variable polarity according to Equation (3).

Figure 3 compares the experimental wavenumber for the absorption and fluorescence maximum as well as the Stokes shift in the tested solvents and those calculated with the regression values collected in Table S2. Perfect linear correlations obtained for all cases indicate that the applied model is valid.

The determined *d*_SB_ coefficients and their standard errors showed that the HBA basicity of the solvent is negligible for all the dyes tested. Regressions without SB yielded slightly higher correlation coefficients *R^2^* than with this parameter.

$$\nu_{Ab}=\left(24,068\pm 139\right)-\left(2183\pm 178\right)SP-\left(800\pm 57\right)SdP-\left(1475\pm 687\right)SA\;with\;n=9\;and\;R^{2}=0.992$$

$$\nu_{Fl}=\left(21,268\pm 179\right)-\left(1944\pm 230\right)SP-\left(1296\pm 74\right)SdP-\left(1494\pm 885\right)SA\;with\;n=9\;and\;R^{2}=0.993$$

$${\Delta \nu }^{SS}=\left(2800\pm 174\right)-\left(240\pm 223\right)SP+\left(495\pm 71\right)SdP+\left(18\pm 858\right)SA\;with\;n=9\;and\;R^{2}=0.936$$

In the case of the merocyanine dyes with the unblocked *N*,*N*-dialkylamino group (compounds **1**, **2**, **3,** and **5**), the general solvent effect on the absorption band was due to polarizability with some contribution by solvent dipolatity and acidity. The weight of the SP term was the greatest for **1** and decreased with the number of methyl groups. An analogous conclusion can be made for merocyanins with a stiffened amino group. The SP term has the lowest value for the derivative with an amino group symmetrically bridged with the aromatic ring (compound **11**). In the group of dyes containing pyrrolidine, piperidine, and morpholine as electron donors, the weight of the SP term is the highest for the latter derivative. 

The analysis shows that all the spectral data are well fitted to the Catalán scale, displaying the correlation coefficients between 0.905 and 0.998. The statistics were only good for compound **7** in terms of the fluorescence maximum and Stokes shift, as well as for dye **13** in terms of absorption. This is probably related to the greater impact of change in the geometry of the amino group on the spectral properties after excitation.

**Scheme 2:**
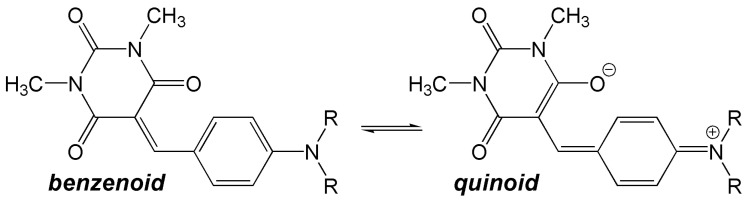
Change in the electronic structure from benzenoid to quinoid for tested dyes.

In general, positive solvatochromism was observed for the dyes tested, which indicated that the excited state is more dipolar than the ground state, so the dipole moment increases upon excitation [51,52,76,77,78]. The intermolecular charge transfer from the dimethylamine group (electron donor) to the barbituric acid moiety (electron acceptor) via the methine group is responsible for their solvatochromic behavior. The solvent polarity stimulates the distribution of electrons in the conjugated bridge between the electron donor and the electron acceptor of the molecules, leading to a change in the electronic structure from benzenoid to quinoid (Scheme 2). The former dominates in the ground state, while the contribution of the latter increases upon excitation. 

**Figure 4:**
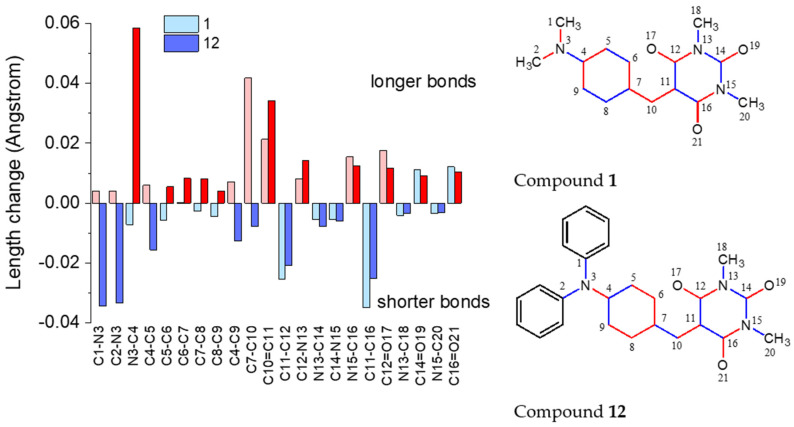
The topology of the studied structures and trend of bond length alternation in dye **1** (light color) and **12** (dark color), where the red color means bond length elongation and the blue color means bond length shortening upon excitation.

To confirm this, the bond length alternation (BLA) was determined based on theoretical calculations, which provide evidence of the geometrical perturbation. Upon excitation, a change in bond length from shorter to longer and vice versa was observed, but shortenings or elongations varied significantly depending on the structure. Figure 4 shows the changes in bond lengths for compounds **1** and **12,** along with the topology of the molecular core. Blue means bond shortening, and red means bond elongation upon excitation. The lengths of particular bonds for selected dyes in the ground and excited states, are summarized in Table S3. The largest change in geometry upon excitation occurs within the methine group, which is the bridge between the aminophenyl group and barbituric acid, i.e., in the central C7-C10-C11 part of the molecule. Changes were also visible in the electron-donating and electron-accepting moieties, which indicated that in the excited state the compounds had an intermediate structure between *benzenoid* and *quinoid*, showing zwitterionic characteristics. Further explanations of the spectroscopic behavior of merocyanine dyes with respect to theoretical calculations are discussed in detail in the next section.

### 3.4. Theoretical Results

**Figure 5:**
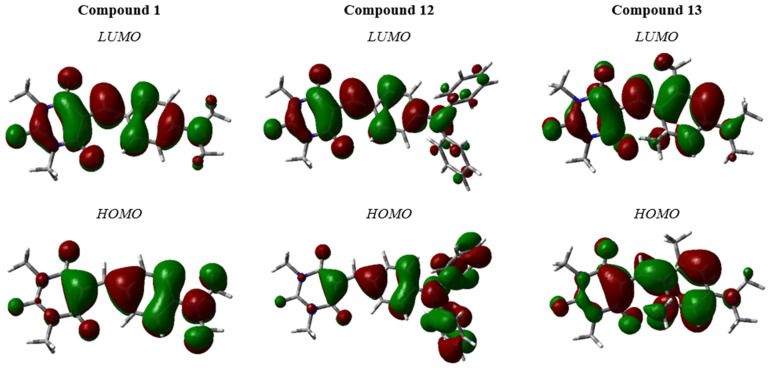
The HOMO—LUMO orbitals of selected merocyanine-type dyes.

To better understand the structural changes and the solvent effect on absorption and fluorescence spectra, computational calculations were performed. According to them, the charge-transfer (CT) excitation corresponds to the HOMO→LUMO transition (Figure 5 and Figure S2). 

For all molecules, the HOMO electrons are mainly delocalized on the benzylidene moiety with changing substituents, while LUMO moves in the direction of pyrimidine-2,4,6(1*H*,3*H*,5*H*)-trione. The electron transfer from the electron donor part towards the electron acceptor, i.e., the barbituric acid ring, suggests that the π-π* transition in combination with the intramolecular charge transfer (ICT) process corresponds to the lowest-lying excited state. The value of energy separation between HOMO → LUMO orbitals (*E*_GAP_) also does not change significantly. The effect of the solvent is slight (${∆E}_{GAP}^{GP-DMF}≅0.2$ eV) (Table S4). Only for **12** does this difference drop to 0.1 eV. The chemical hardness (*η*) of all compounds is low, so they should be treated as soft molecules with high reactivity. Moreover, these dyes should easily form covalent bonds during various chemical processes, as indicated by electronegativity (χ) values greater than 4.1.

**Figure 6:**
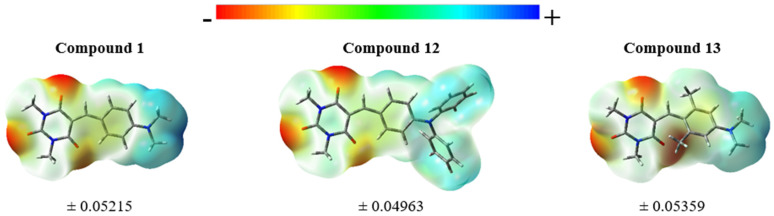
The MEP surfaces.

Molecular electrostatic potential (MEP) surface analysis was also performed to identify sites susceptible to nucleophilic (positive, blue areas) and electrophilic (negative, red, and yellow areas) substitution (Figure 6 and Figure S3). Changing the substituents in the benzylidene part does not change the position of the blue and red zones. The oxygen atoms in the barbituric acid moiety are the most negative site of the molecules, and the value of the charge is slightly different (~−0.05 au). The maximum positive region is localized on substituents attached to the styrene part, indicating a possible site for a nucleophilic attack. 

**Figure 7:**
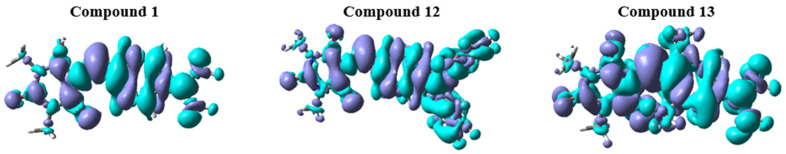
Density difference plot. The blue (purple) zones indicate density decrease (increase) upon electronic transition.

To determine the exact effect of the molecular orbital other than the HOMO→LUMO and the description of the nature of electronic states, the density variation upon photoexcitation (Δ*ρ*(r)) was computed for the first electronic transitions (Figure 7 and Figure S4). 

For all molecules, the plots of Δ*ρ*(r) show that the density depletion zones (blue) occur within substituents that change the type of compound. These regions of density increment (purple) are mainly visible on the carbon π-electron bridge and phenyl ring. At the same time, both the first and second zones are slightly dependent on the solvent polarity. However, the values of *D*_CT_ and *q*_CT_ depend on the environment (Table S5). In each case, the transition from the gas phase to toluene is accompanied by an increase in the *q*_CT_ value, and next, a decrease is observed in more polar solvents. The compounds **1**–**11** have a similar *q*_CT_ value, ranging from 0.425 e (for **4** in GP) to 0.481 e (for **7** in DMF). However, for dyes **12** and **13,** the amount of charge transferred is significantly higher and exceeds 0.510 e. The charge-transfer distance exceeds 2.1 Å and increases as a function of the environment polarity. For all molecules, the value of *D*_CT_ does not exceed 2.636 Å, except dye **12**, for which it ranges from 3.142 Å (in GP) to 3.844 Å (in DMF). The *D*_CT_ and *q*_CT_ value indicates the charge-transfer character of compounds. At the same time, it confirms the pure contributions from the HOMO→LUMO transition (99%).

Considering the free energy of solvation (Δ*G_solv_*, Table S6), all molecules are characterized by Δ*G_solv_* < −17 kcal/mol. Although the value of Δ*G_solv_* decreases with the increase in the medium polarity, the obtained values indicate the lack of solvent influence on solubility.

In Table S7, theoretical one-photon absorption spectra are presented ($\lambda_{max}^{Ab}$). The use of the PBE0 function leads to a slight shift in the position of the maximum absorption band; however, the average error with respect to experimental values does not exceed 2.26 nm. The exception is for the **8** molecule, for which $\Delta \lambda_{max}^{Ab}$ is greater than 10 nm. Employing the cLR model causes a hypsochromic shift, and the error increases to 11.34 nm. Moreover, the graphical representation of the theoretically determined absorption bands (Figure S5) is in close agreement with the experimental ones. Similarly to the measured values, for derivative **12,** there is also an additional peak of low intensity at approximately 310 nm. However, for the theoretical band, it is one peak, and for the experimental band, it is two, with the more intense peak occurring at approximately 290 nm. Based on the density variation upon photoexcitation analysis, for all derivatives, the intense absorption band in the range of 450 nm–490 nm is associated with the HOMO→LUMO transition. However, the additional peaks for compound **12** are related to influences from other orbitals. In this case, they come from the HOMO-4→LUMO (89%) and HOMO→LUMO+1 (9%) transitions.

Considering the impact of the dialkylamino group on the spectral characteristics of the tested dyes, one can draw conclusions fitting the experimental data. Firstly, the replacement of the NMe_2_ group with NEt_2_, N(Me)Et, and N(*n*-Bu)_2_ shifts the absorption maximum by a few nanometers (2–5 nm). Secondly, the introduction of a methyl substituent on the phenyl ring (**4**) induces a bathochromic effect with respect to **1** by ~8 nm. The presence of two methylene groups (**13**) increases the shift to ~15 nm with respect to model dye **1**. In turn, the N(Ph)_2_ substitution shifts $\lambda_{max}^{Ab}$ by ca. 7 nm in toluene, wherein this difference decreases with increasing solvent polarity and amounts to −4 nm in DMF. Thus, replacing group NMe_2_ with N(Ph)_2_ produces a hypsochromic effect. Like the experimental results, a bathochromic shift of about 16 nm is caused by the stiffening of the amino group by two methylene bridges with the phenyl ring (dye **11**). In contrast, the stiffening of the amino group only on one side by two (dye **9**) or three (dye **10**) methylene units, respectively, shifts the absorption band by 13 nm and 10 nm in relation to compound **1**. A significant shift in $\lambda_{max}^{Ab}$ is also not observed for molecules **6** and **7**. An interesting observation comes from the comparison of **11** and **13**. The presence of julolidine in the molecule causes the absorption band to lie almost in the same range as **13**. Accordingly, it might be concluded that the presence of methylene substituents attached to the phenyl ring is a factor that significantly affects the location of $\lambda_{max}^{Ab}$. More importantly, as for the experimental values, the vertical and cLR values indicate that $\lambda_{max}^{Ab}$ varies monotonically with the solvent polarity (Table S8). It follows from the above that in polar solvents, there is greater polarization and better stabilization of the excited state of merocyanine dyes, which results in a decrease in the excitation energy (Δ*E*) regardless of the substituent. This tendency corresponds to the polarity of the CT state (${\Delta \mu }_{g-CT}$). As shown in Table S9, independently of the solvent, a $\mu_{CT}>\mu_{g}$ relation is always obtained, which is characteristic of positive solvatochromism. The average value of ${\Delta \mu }_{g-CT}$ is 5–6 D. However, the factor that minimizes CT polarity in solutions is the presence of julolidine and one methyl group attached to the phenyl ring (${\Delta \mu }_{g-CT}<$ 5 D). In turn, the factor maximizing this value is the presence of the −N(Ph)_2_ substituent (${\Delta \mu }_{g-CT}>$ 9 D). Based on these observations, there should be no pure electrostatic contributions to the solvent–solute interactions. Moreover, taking into account the MEP analysis, regardless of the structure of the substituent, we found that H-bonds and self-aggregation should also not occur.

Moreover, the density variation upon photoexcitation suggests that in the case of intramolecular CT transition, the influence of the dispersion contribution on the position of the electronic absorption band is less important than the impact of the electrostatic effects.

Analysing the TDDFT emission spectra (Table S10), the use of the cLR model leads to a bathochromic shift in the fluorescence maximum in relation to the experimental data. The average relative error is 10.65 nm. A significant improvement is obtained for vertical values that oscillate around experimental data, and the error falls to 1.98 nm. Analogously to $\lambda_{max}^{Ab}$, the position of the fluorescence band maximum is influenced by the type of solvent, and this behavior is monotonic. However, in experimental values, one can notice the disturbances of this behavior in the case of acetone and the non-monotonic behavior for molecule **7**. Nevertheless, it should be thought that the tested compounds have a charge-separated excited state and a neutral ground state because of the solute–solvent electrostatic interaction. The influence of the substituents on the location of the $\lambda_{max}^{Fl}$ maximum does not remain in accordance with the conclusions drawn from the position of $\lambda_{max}^{Ab}$. In this case, the presence of N(Ph)_2_ induces a significant bathochromic shift, while the presence of pyrrolidine and piperidine induces a hypsochromic shift. This relation is analogous to the value of ${\Delta \nu }^{St}$. Both for experimental and theoretical values, the highest value of ${\Delta \nu }^{SS}$ is characterized by **12**, while the lowest is **9**.

## 4. Conclusions

The group of 13 merocyanine dyes was synthesized, and their structures were confirmed by spectroscopic analysis. The tested compounds had a push–pull structure with 1,3-dimethylbarbituric acid moiety as an electron acceptor and an alkylamino substituent in the *para* position of the benzene ring as an electron donor. Steady-state absorption and fluorescence measurements were used to investigate their linear optical properties. The observed redshift of their absorption and fluorescence bands was due to both the chemical structure and the influence of aprotic solvents. Stiffening the alkylamino group with an aliphatic bridge and an aromatic ring promotes charge transfer in the ground state. The linear solvation energy (LSE) analysis shows that dipolarity/polarizability interactions mainly contribute to solvent-induced spectral shifts. Moreover, with the help of theoretical calculations, it was found that the electronic structure of the merocyanine dyes goes from a nonpolar *benzenoid* structure to a dipolar *quinoid* structure with a change in the donor ability of the end group and the solvent polarity. The delocalization of the lone electron pair on nitrogen in the dialkylamino group towards barbituric acid moiety leads to the presence of zwitterionic resonance forms and the enhancement of the CT character of the dyes. This is especially important for molecules that are twisted in the ground state and which are more greatly flattened in the excited state.

In summary, although the specific applications of the tested 5-(4-substituted-arylidene)-1,3-dimethylpyrimidine-2,4,6-triones are not directly addressed, their structural and chemical properties, as well as evidence for similar compounds, suggests their potential in the medicinal chemistry, drug development and therapy, and materials science. Their diverse structural modifications and biological activities may be involved in the development of antimicrobials, antidiabetic and anticancer treatments, and the synthesis of complex heterocyclic structures. Nevertheless, further studies are required to explore structure–activity relationships, pharmacological profiles, and synthetic methodologies.

## Figures and Tables

**Table 1:** Photophysical data for compounds **1**–**13**; absorption $\left(\lambda_{max}^{ab};nm\right)$ and fluorescence maxima $\left(\lambda_{max}^{fl};nm\right)$, shift (Δν^SS^; cm^−1^), molar absorption coefficient (*ε_max_*; 10^4^ M^−1^cm^−1^) and full width at half maximum of the absorption band (FWHM^ab^; cm^−1^).

No	$\lambda_{max}^{ab}$	*ε_max_*	FWHM*^ab^*	$\lambda_{max}^{fl}$	Δν^SS^	$\lambda_{max}^{ab}$	*ε_max_*	FWHM*^ab^*	$\lambda_{max}^{fl}$	Δν^SS^	$\lambda_{max}^{ab}$	*ε_max_*	FWHM*^ab^*	$\lambda_{max}^{fl}$	Δν^SS^
	Toluene					THF					DMF				
**1**	452	7.03	2091	516	2744	453.5	7.15	2204	523	2930	463.5	7.17	2271	543	3159
**2**	457	9.03	1916	517	2539	459	8.95	2384	526	2775	469	7.93	2102	542	2872
**3**	454.5	7.89	2011	522	2845	455	7.58	2142	529	3074	465	7.03	2187	545	3157
**4**	464	5.99	2214	539	2999	466.5	5.87	2295	541	2952	477.5	5.69	2326	554	2892
**5**	459	8.10	1909	524	2703	461.5	8.43	2005	534	2942	471	7.44	2082	548	2983
**6**	459	7.83	1936	528	2847	460	7.68	2039	531	2907	468.5	7.36	2126	544	2962
**7**	456.5	5.87	2301	532	3109	458	6.42	2896	545	3485	468	5.66	2434	548	3119
**8**	441.5	5.15	2682	497	2529	443.5	4.82	2822	520	3317	453.5	4.96	2928	543	3635
**9**	468	6.93	2123	535	2676	469.5	6.42	2190	535	2608	482	6.82	2169	550	2565
**10**	464.5	7.81	1900	525	2481	466.5	7.72	2023	533	2675	477	7.65	2064	552	2848
**11**	476	9.33	1730	537	2386	479	8.88	1772	544	2494	489.5	8.60	1859	558	2508
**12**	463.5	3.67	2644	583	4422	458	2.59	2818	644	6306	460	3.05	2977	680	7033
**13**	476	2.27	2877	531	2176	473	1.94	3744	544	2759	481	1.73	3450	560	2933

**Table 2:** Fluorescence quantum yield (FQY) data in toluene, THF and DMF.

Compound	FQY (%)		
	Toluene	THF	DMF
**1**	0.031	0.035	0.040
**2**	0.029	0.045	0.046
**3**	0.034	0.052	0.041
**4**	0.024	0.025	0.024
**5**	0.043	0.060	0.063
**6**	0.039	0.046	0.044
**7**	0.043	0.049	0.046
**8**	0.033	0.035	0.045
**9**	0.049	0.035	0.039
**10**	0.046	0.036	0.048
**11**	0.067	0.054	0.055
**12**	0.257	0.534	0.109
**13**	0.048	0.001	0.005

## Data Availability

Data are contained within the article and Supplementary Materials.

## Supplementary Materials

The following supporting information can be downloaded at: https://www.mdpi.com/article/10.3390/ma17102447/s1, HPLC chromatograms; ^1^H and ^13^C NMR spectra; IR spectra; Figure S1: Electronic absorption spectra of **1** upon irradiation; Figure S2–S5: Graphical representation of theoretical calculations; Table S1: Photophysical parameters; Table S2: Data for the multiple linear regression analysis; Table S3: Calculated geometries in THF; Table S4: Frontier orbital energies; Table S5: CT parameters; Table S6: Values of free energies of solvation; Table S7: Values of vertical excitation energies; Table S8: Values of cLR-corrected excitation energies; Table S9: Calculated values of dipole moments; Table S10: Values of vertical and cLR-corrected de-excitation energies.
