# Supplementary material for: The Influence of the Alkylamino Group on the Solvatochromic Behavior of 5-(4-substituted-arylidene)-1,3-dimethylpyrimidine-2,4,6-triones: Synthesis, Spectroscopic and Computational Studies
Source: Materials (Basel). 2024 May 19;17(10):2447. doi: 10.3390/ma17102447 (PMC11123250; doi:10.3390/ma17102447)
Supplement: Supplementary file 1 [file materials-17-02447-s001.zip › materials-3011430-supplementary.pdf]

SUPPORTING INFORMATION

The Influence of the Alkylamino Group on the Solvatochromic Behavior of 5-(4-substituted-arylidene)-1,3-dimethylpyrimidine-2,4,6-triones. Synthesis, Spectroscopic and Computational Studies

Ilona Pyszka [1], Przemysław Krawczyk [2] and Beata Jędrzejewska [1,*]

[1] Faculty of Chemical Technology and Engineering, Bydgoszcz University of Science and Technology, Seminaryjna 3, 85-326 Bydgoszcz, Poland; ilona.pyszka@pbs.edu.pl
[2] Faculty of Pharmacy, Collegium Medicum, Nicolaus Copernicus University, Kurpińskiego 5, 85-950 Bydgoszcz, Poland; przemekk@cm.umk.pl
* Correspondence: beata@pbs.edu.pl; Tel.: +48-52-374-90-46

Table of contents

	page
HPLC chromatograms	2-6
^{1}H and ^{13}C NMR spectra	7-19
IR spectra	20-32
Photophysical Parameters of Compounds **1-13** – Table S1	33-35
Data for the multiple linear regression analysis of ν_{Ab}, ν_{Fl} and $\Delta\nu^{SS}$ of **1-13** in 9 solvents as a function of the Catalán four-parameter solvent scale – Table S2	36-37
Electronic absorption spectra of **1** upon irradiation – Figure S1	37
The HOMO – LUMO orbitals of tested compounds – Figure S2	38
The MEP surfaces. – Figure S3	39
Density difference plot. The blue (purple) zones indicate density decrease (increase) upon electronic transition – Figure S4	39
Graphical representation of the calculated absorption spectra – Figure S5	40
Calculated geometries of the merocyanine dyes in THF – Table S3	41-43
Frontier orbital energies in selected solvents. All values are given in eV – Table S4	44-45
CT parameters for the bright low-lying excited state – Table S5	45
Values of free energies (ΔG_{solv}) of solvation in kcal/mol obtained using SMD solvation model – Table S6	46
Values of vertical excitation energies – Table S7	46
Values of cLR corrected excitation energies – Table S8	47
Calculated values of dipole moments (in D) for the ground and CT excited state – Table S9	47
Values of vertical and cLR corrected de-excitation energies – Table S10	48

HPLC chromatogram of **1**:

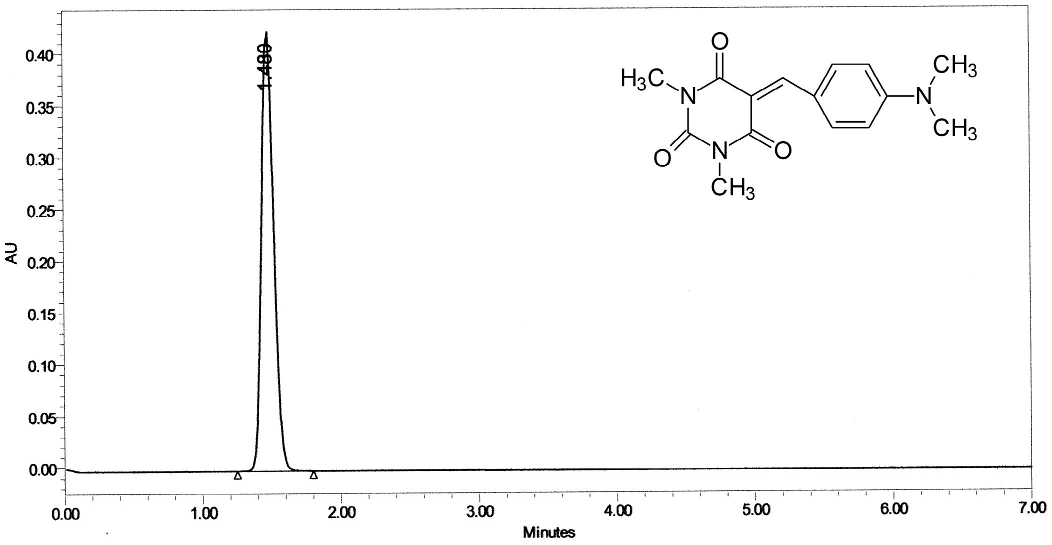

HPLC chromatogram of **2**:

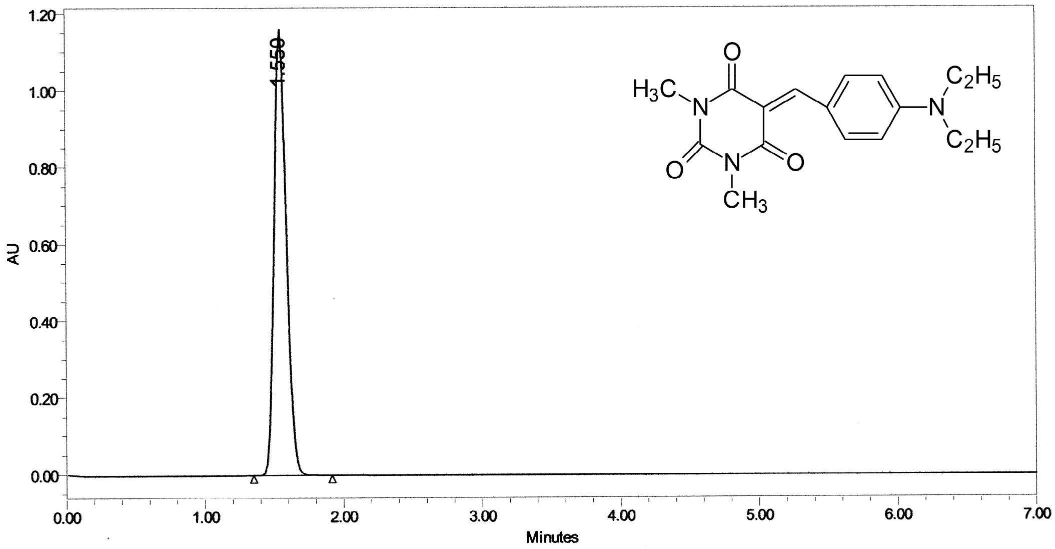

HPLC chromatogram of **3**:

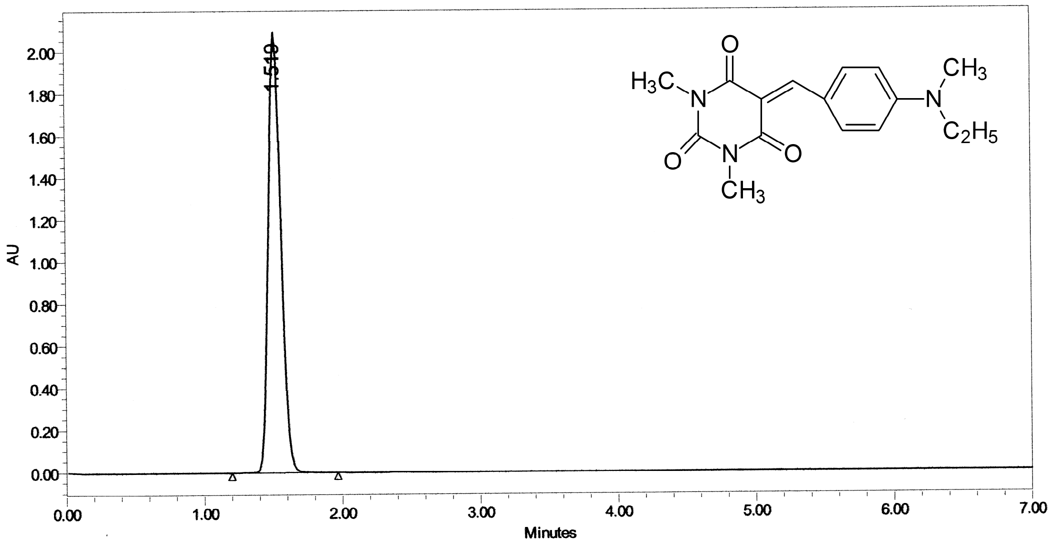

HPLC chromatogram of **4**:

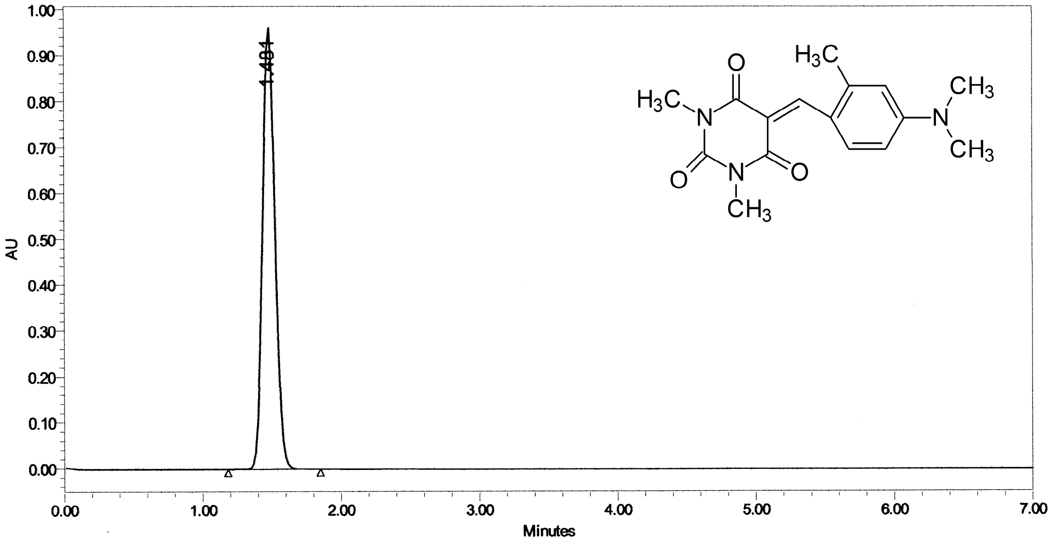

HPLC chromatogram of **5**:

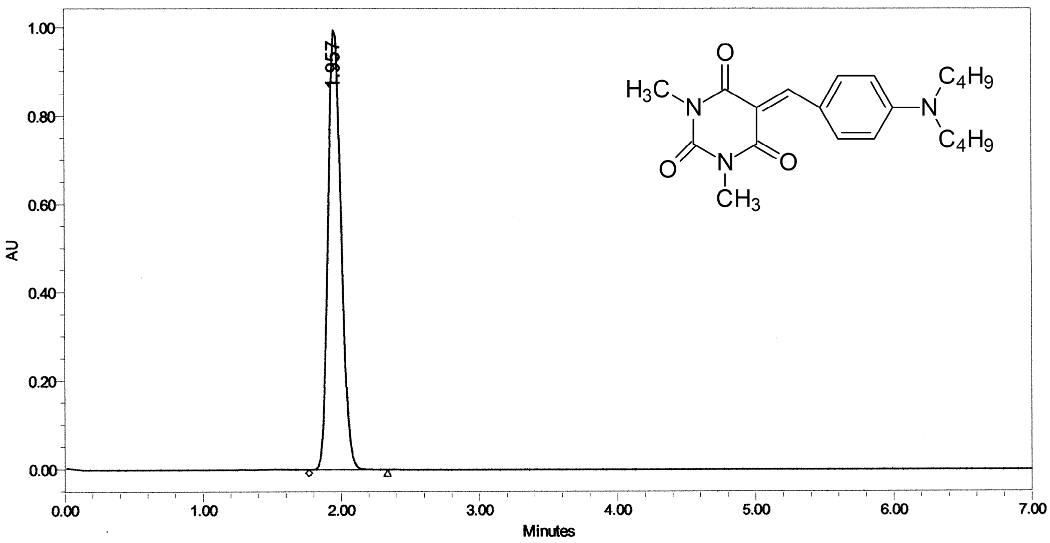

HPLC chromatogram of **6**:

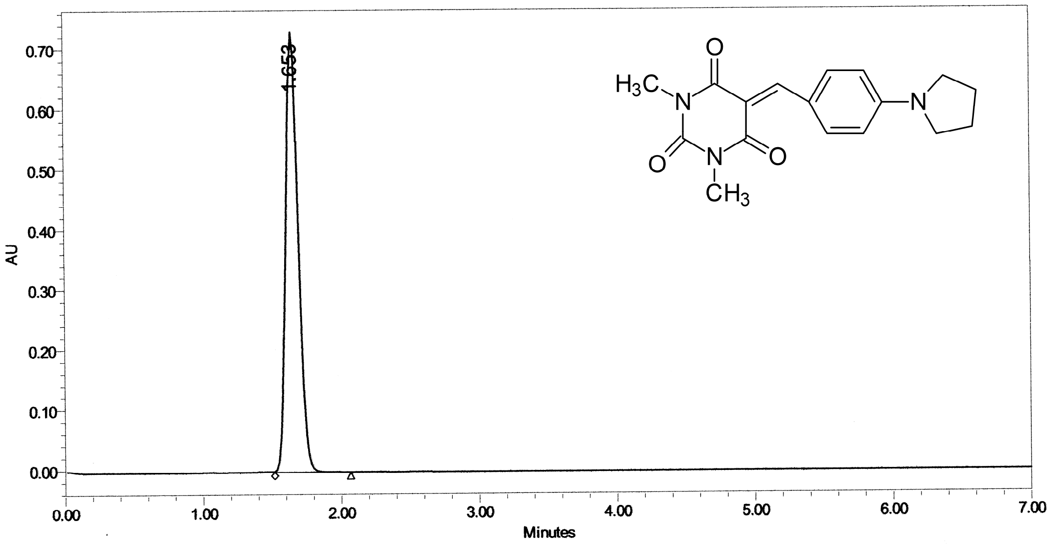

HPLC chromatogram of **7**:

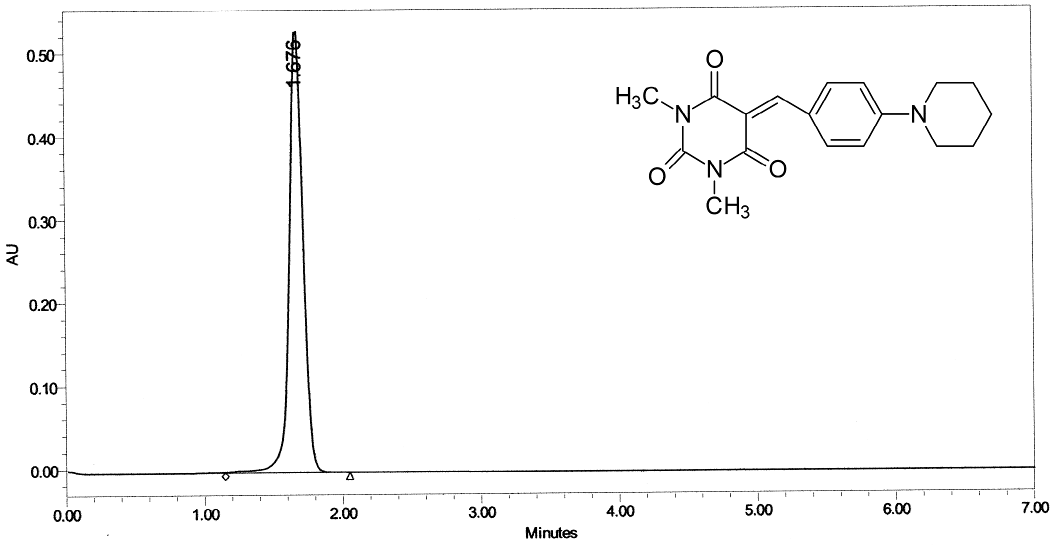

HPLC chromatogram of **8**:

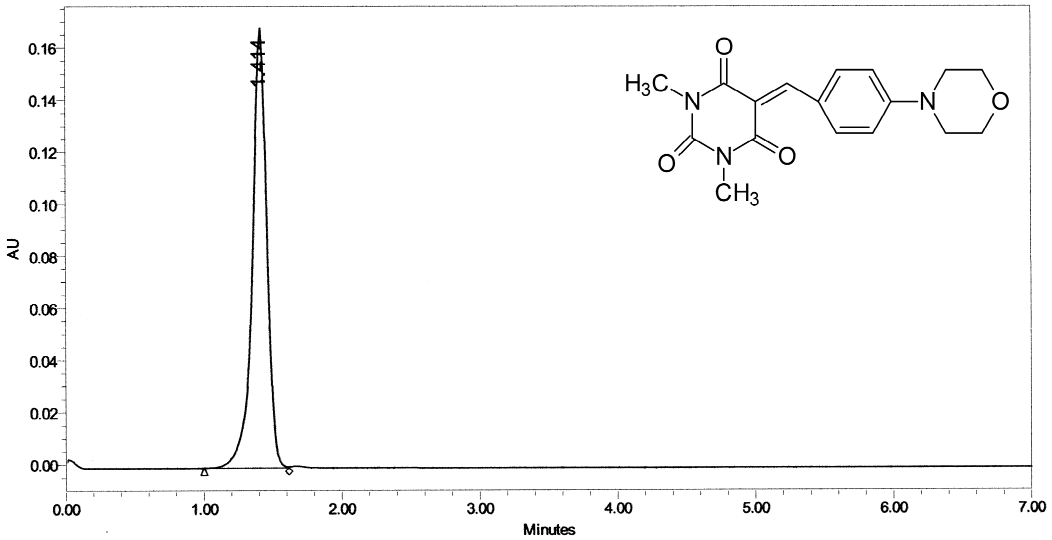

HPLC chromatogram of **9**:

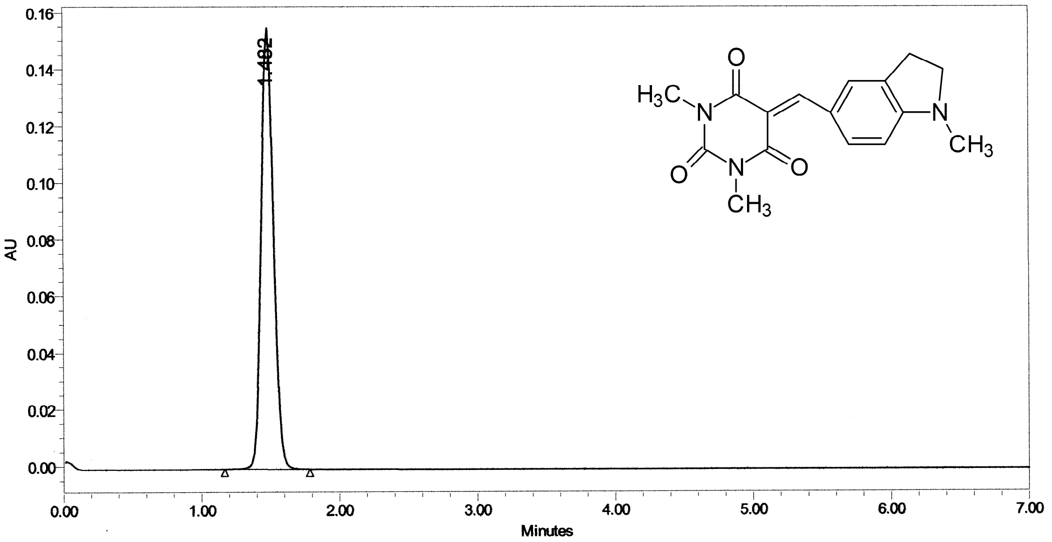

HPLC chromatogram of **10**:

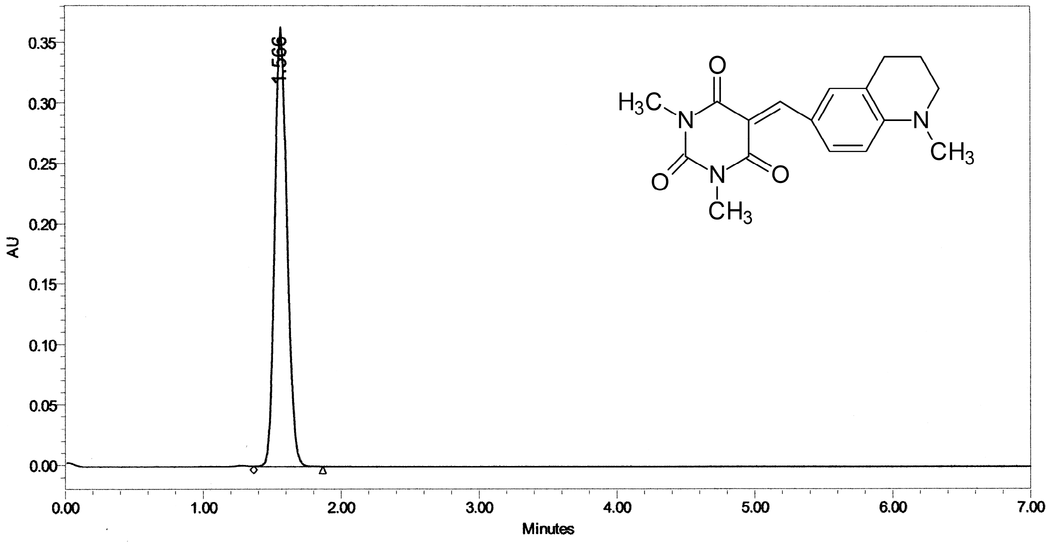

HPLC chromatogram of **11**:

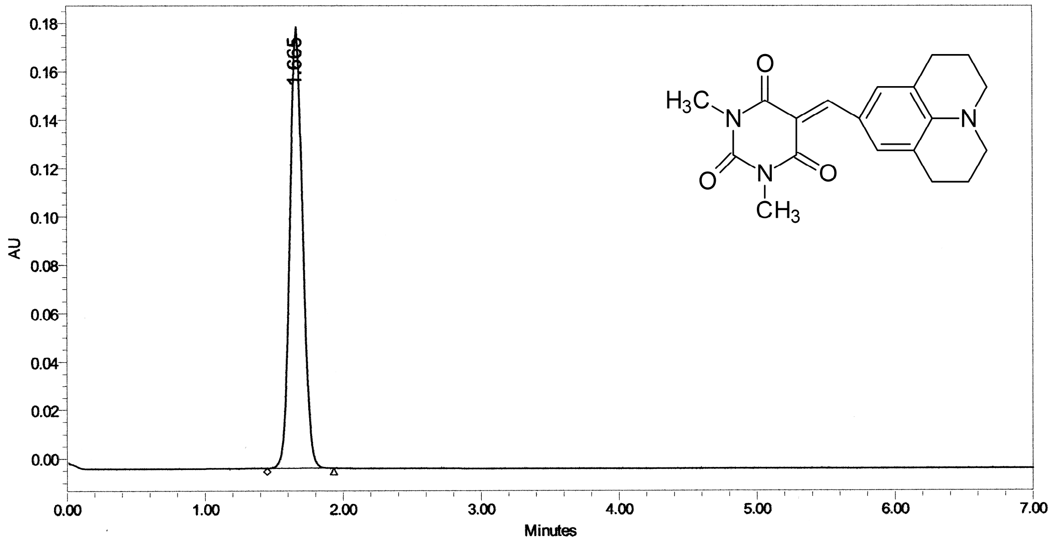

HPLC chromatogram of **12**:

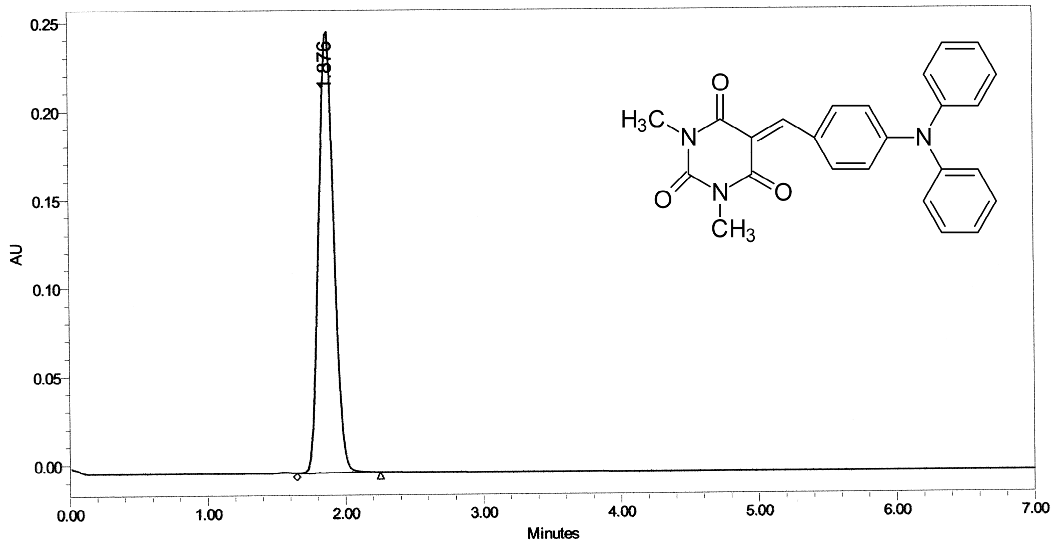

HPLC chromatogram of **13**:

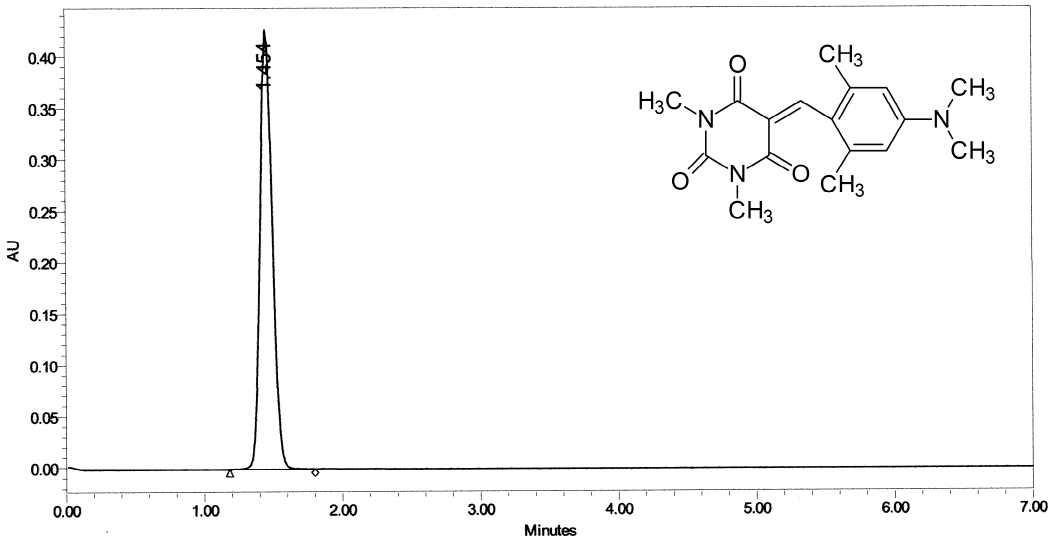

^{1}H NMR spectrum of **1**

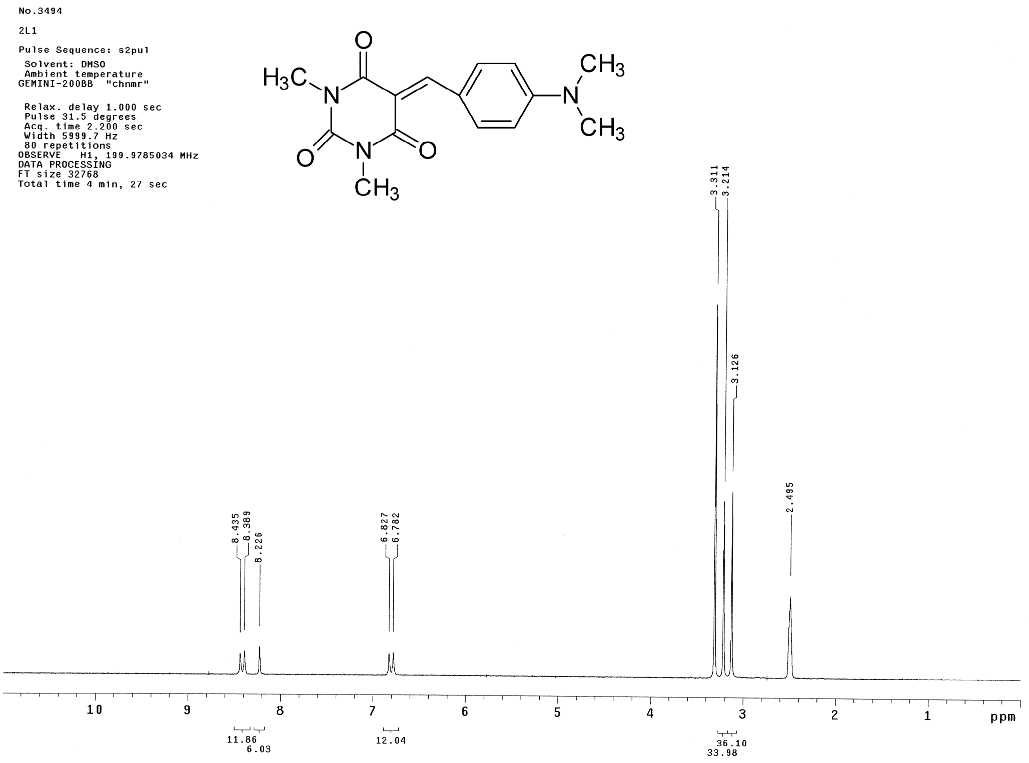

^{13}C NMR spectrum of **1**

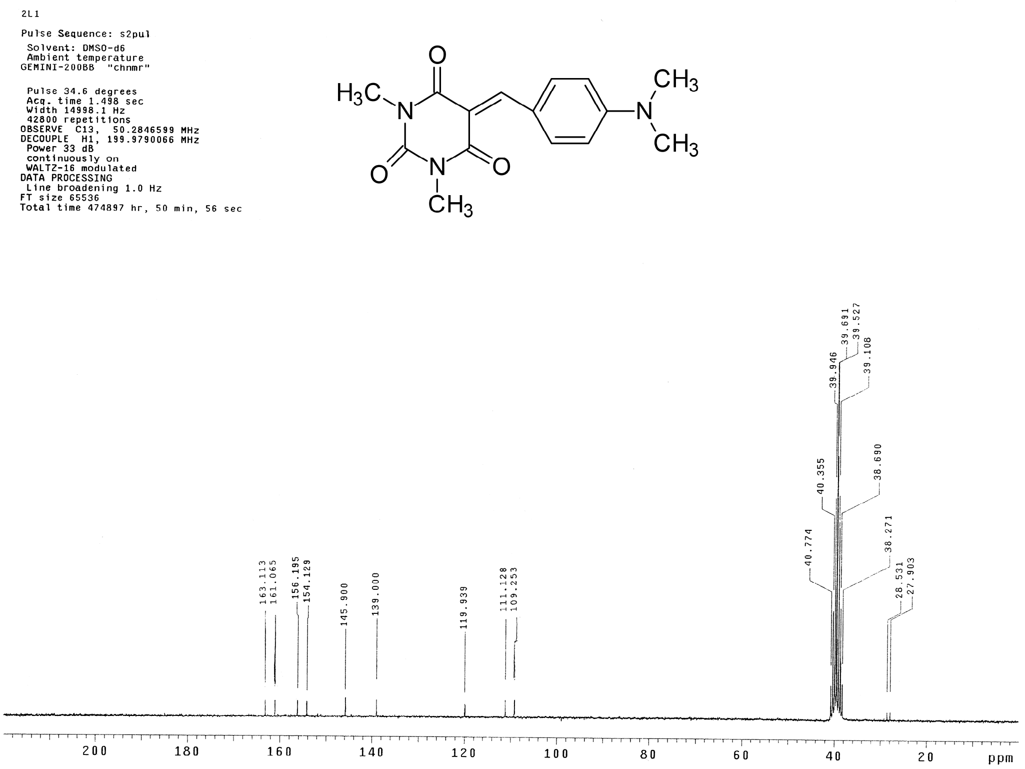

^{1}H NMR spectrum of **2**

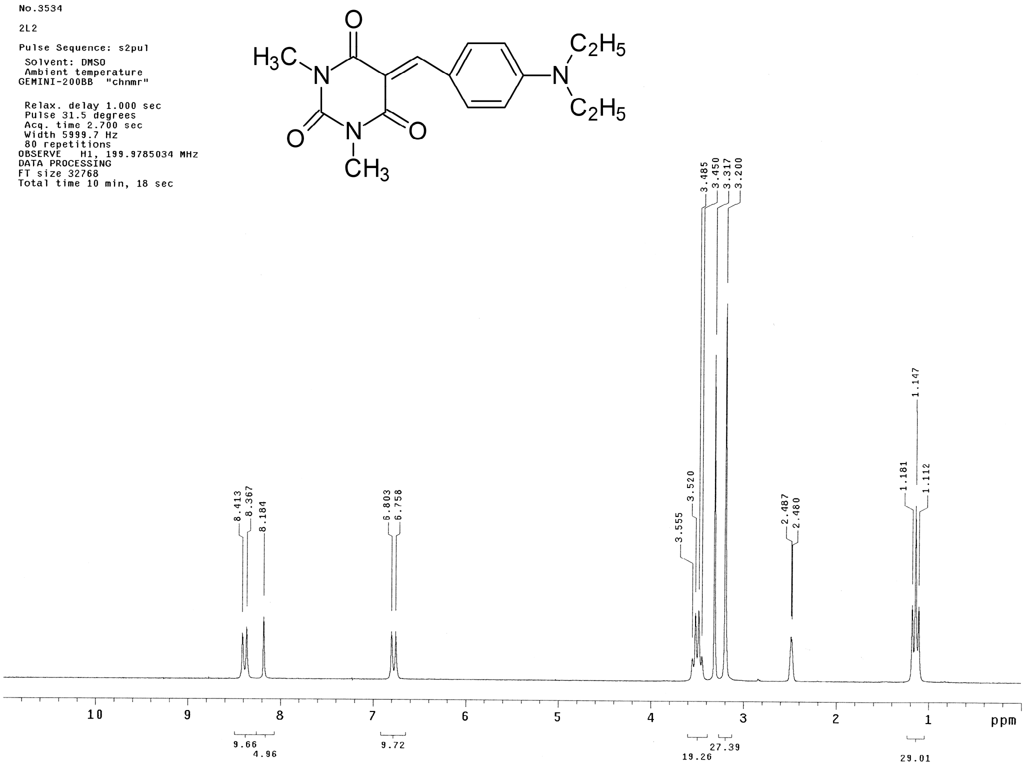

^{13}C NMR spectrum of **2**

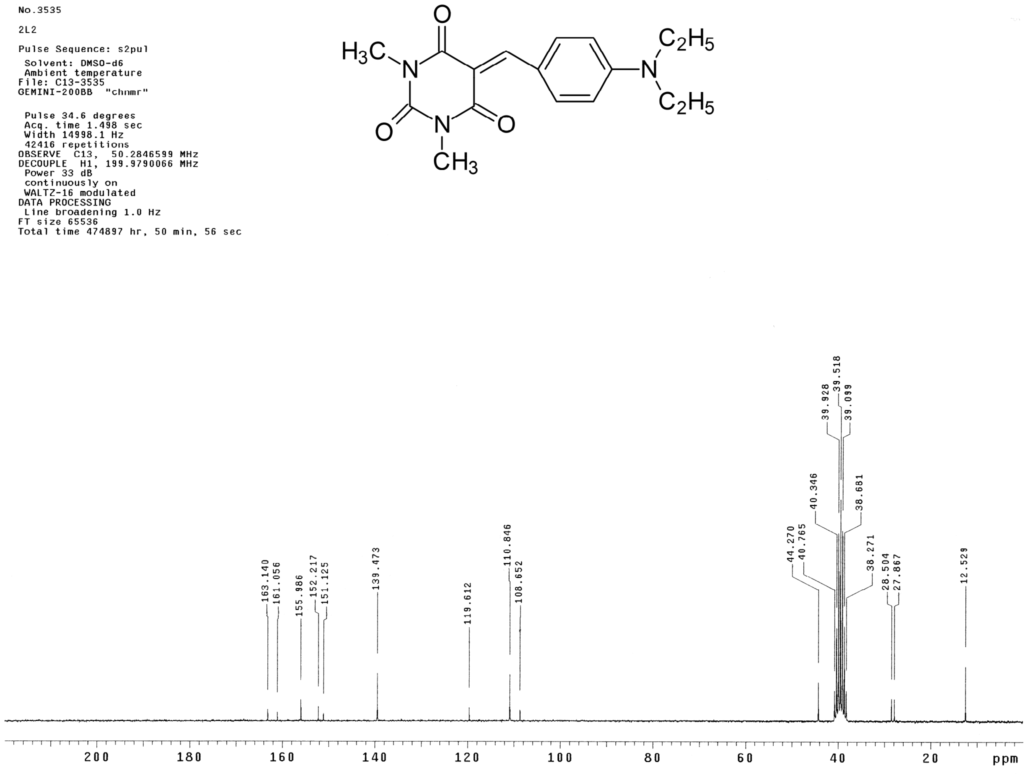

^{1}H NMR spectrum of **3**

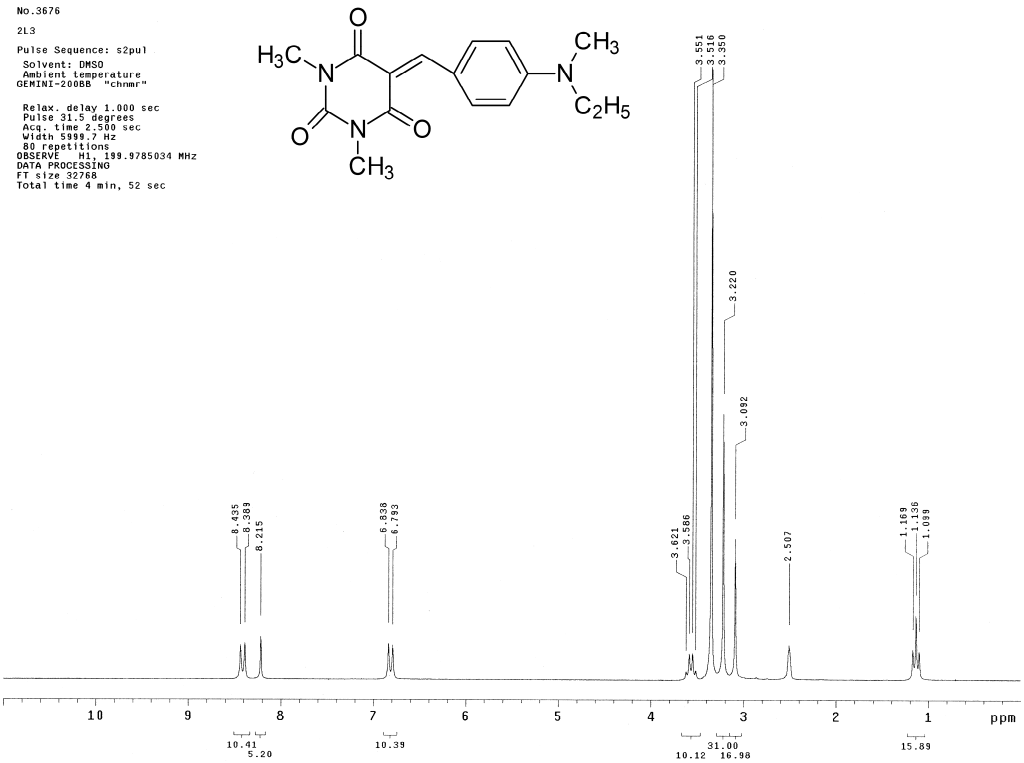

^{13}C NMR spectrum of **3**

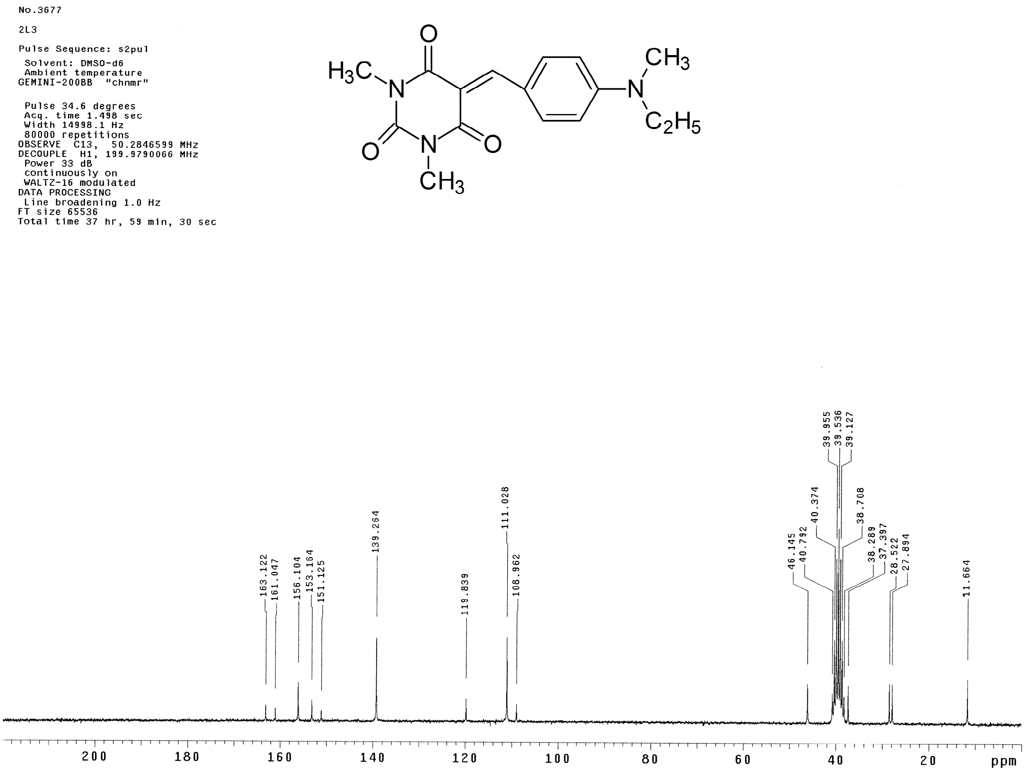

^{1}H NMR spectrum of **4**

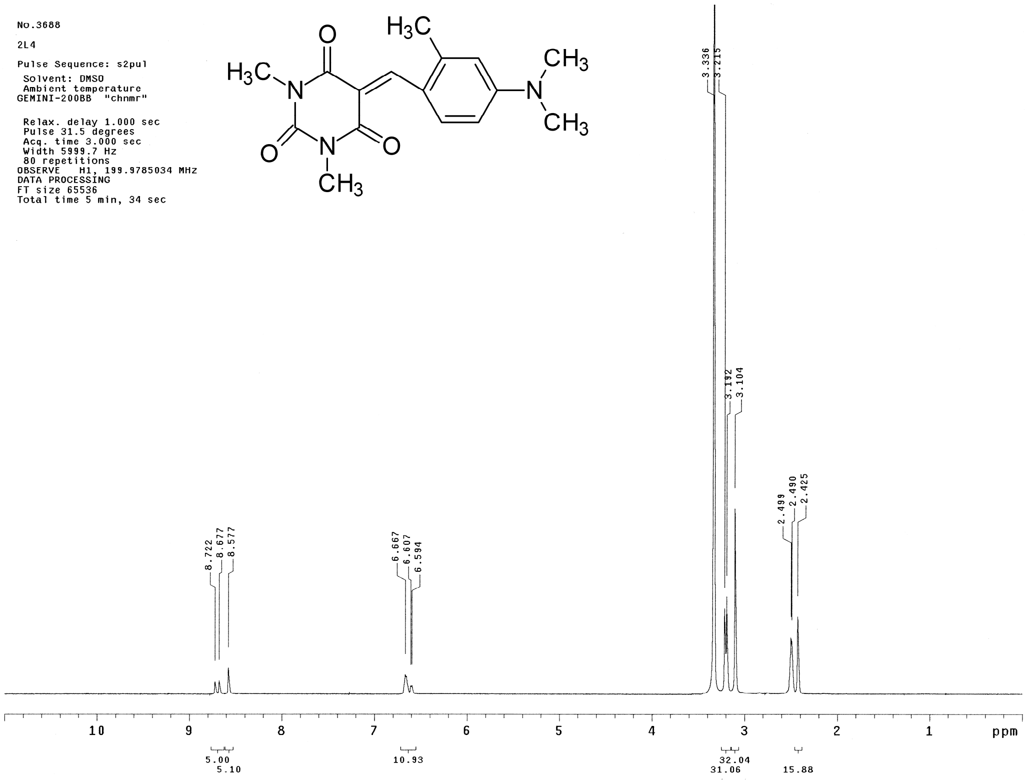

^{13}C NMR spectrum of **4**

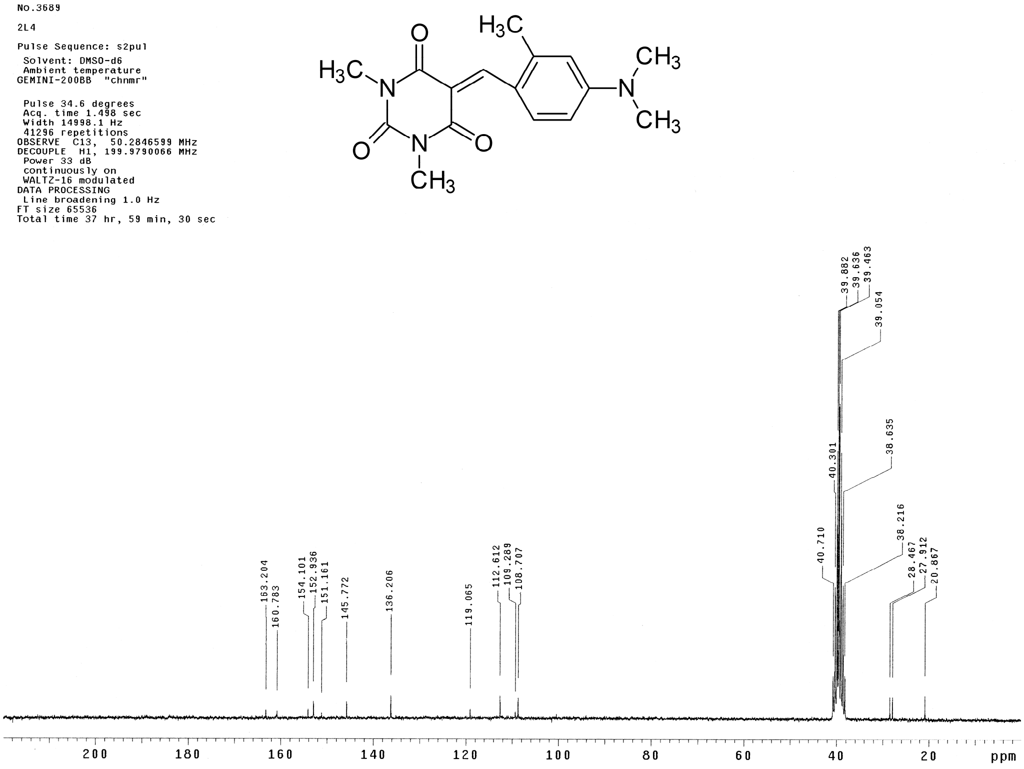

^{1}H NMR spectrum of **5**

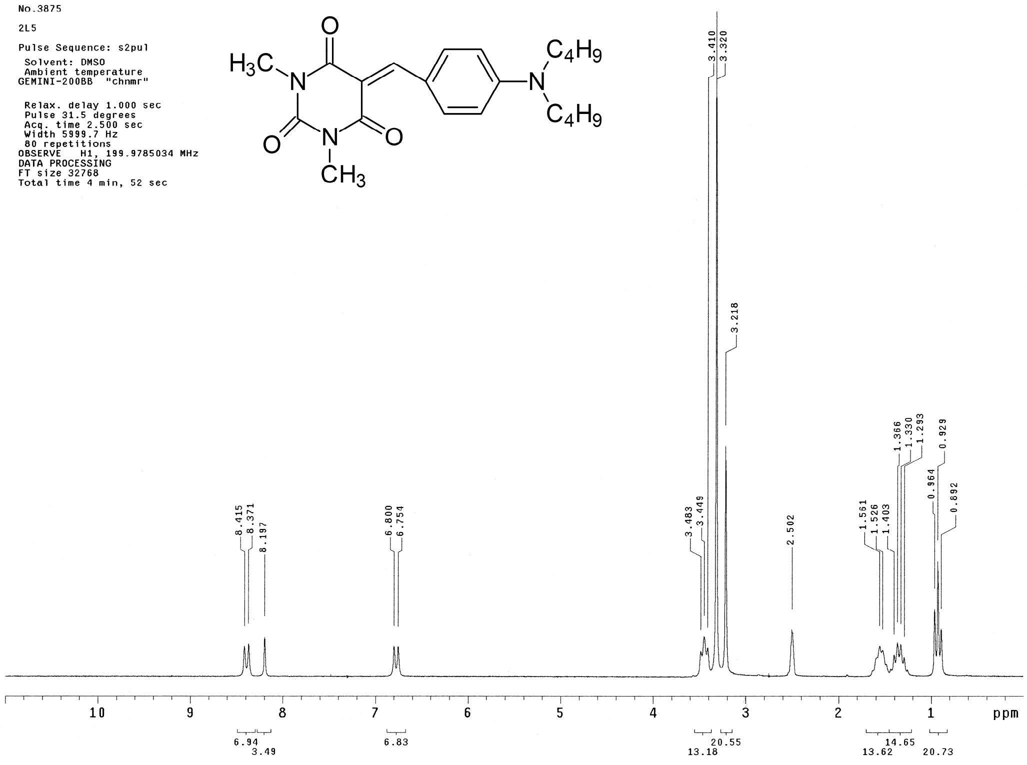

^{13}C NMR spectrum of **5**

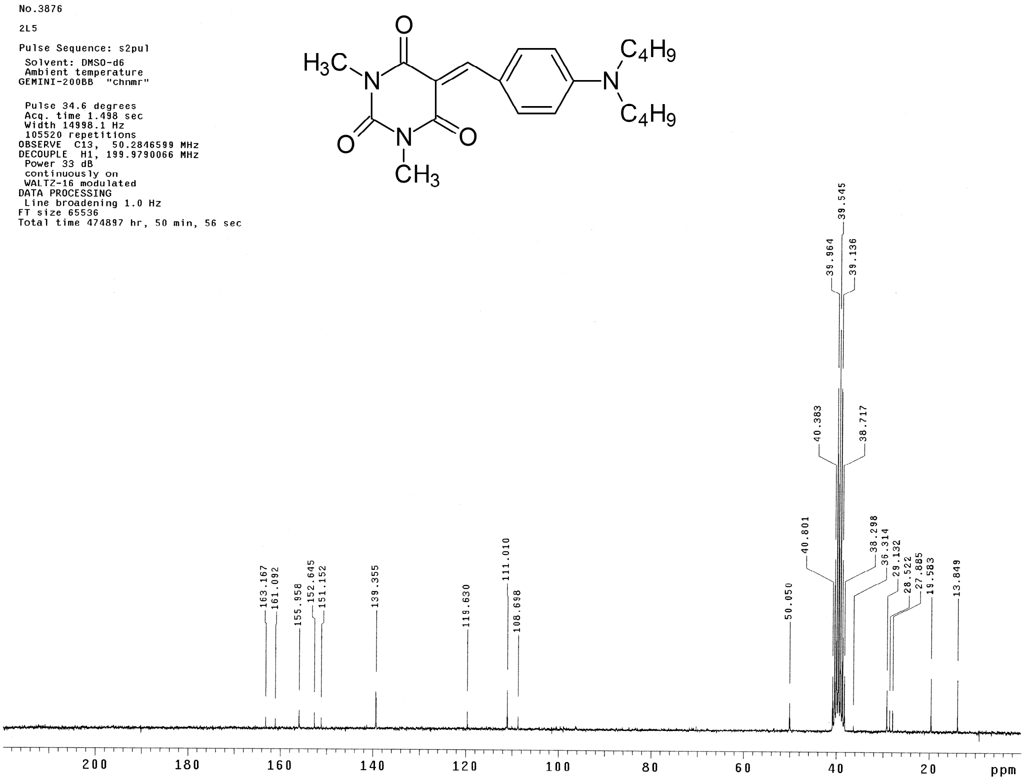

^{1}H NMR spectrum of **6**

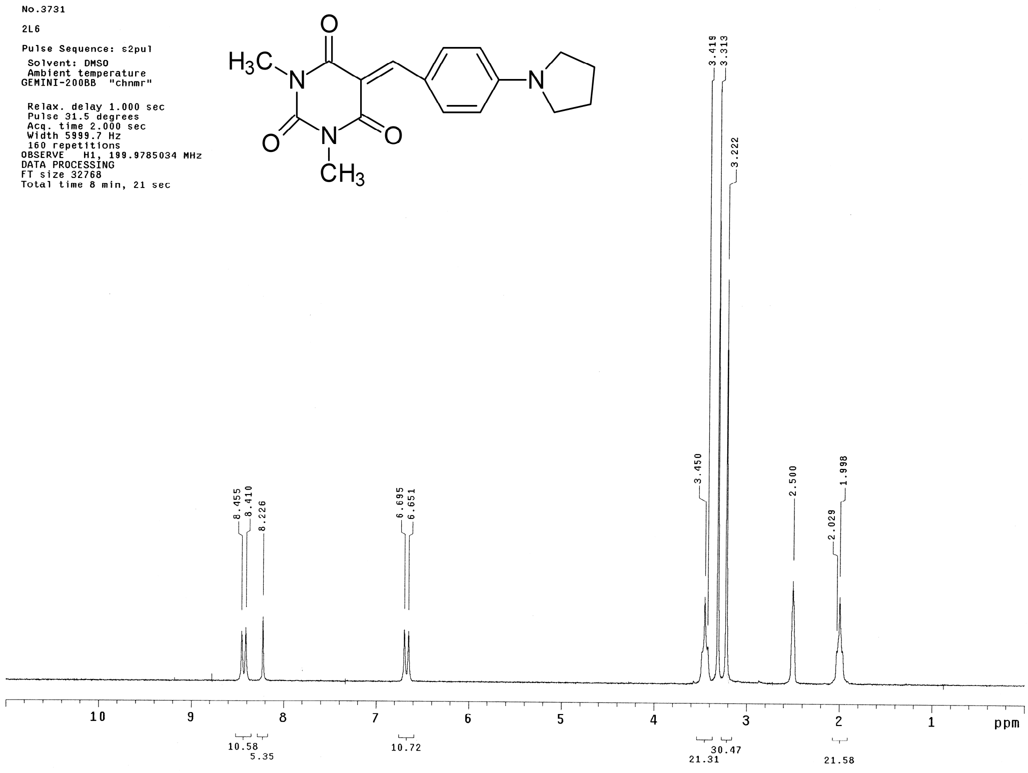

^{13}C NMR spectrum of **6**

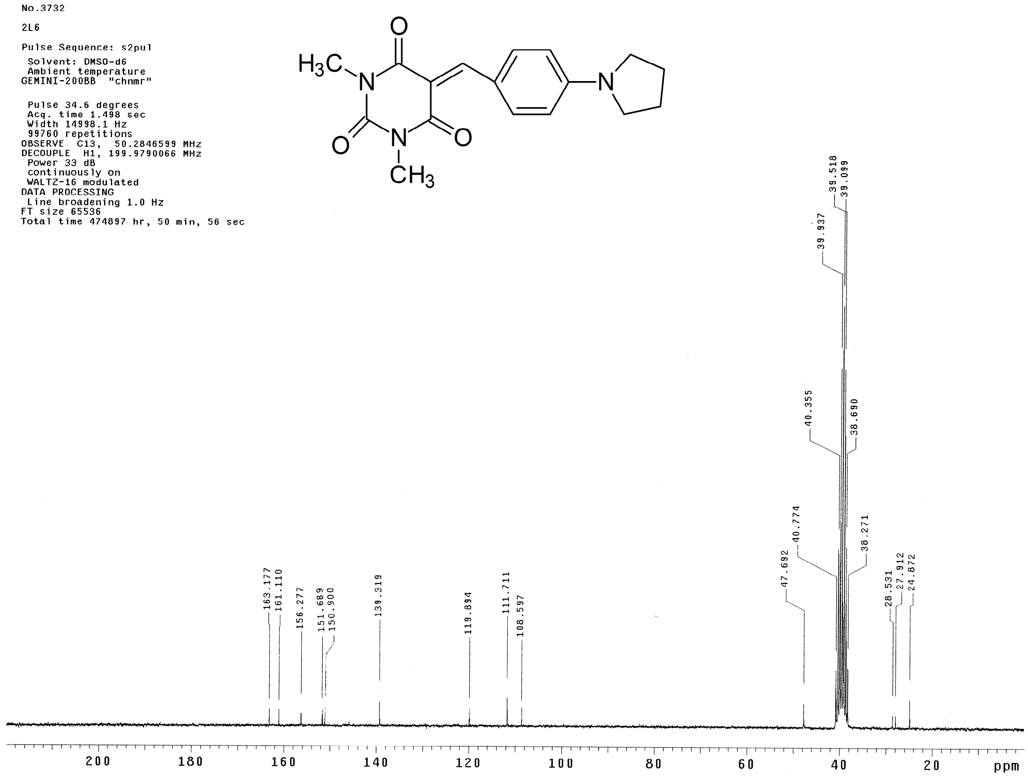

^{1}H NMR spectrum of **7**

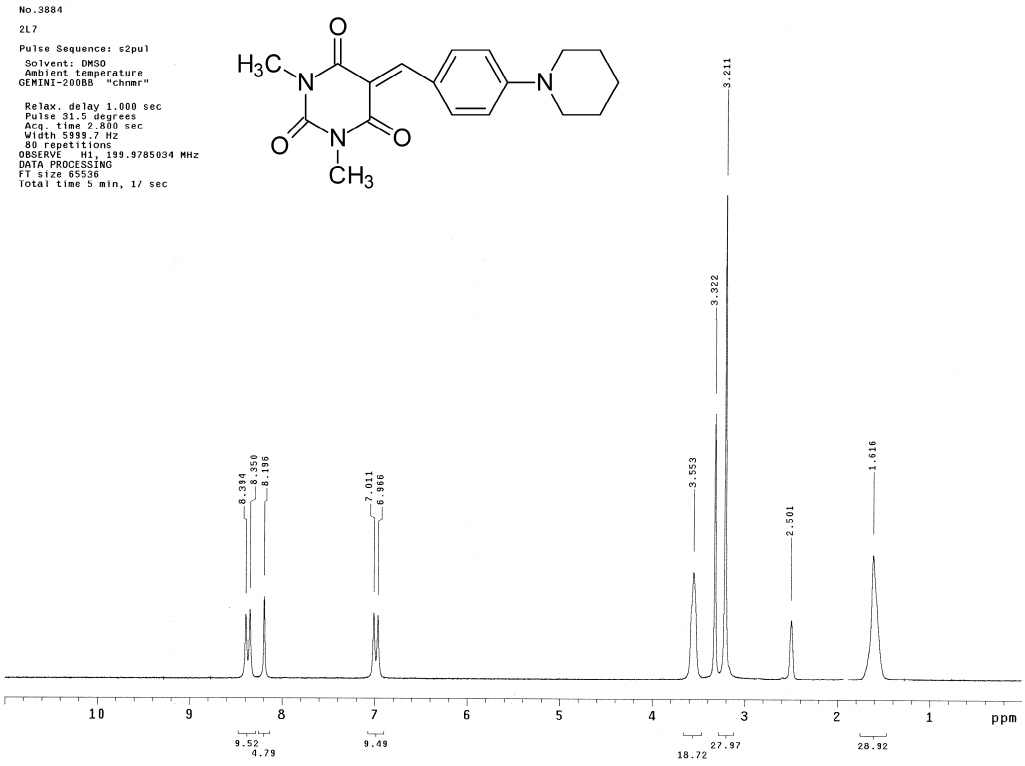

^{13}C NMR spectrum of **7**

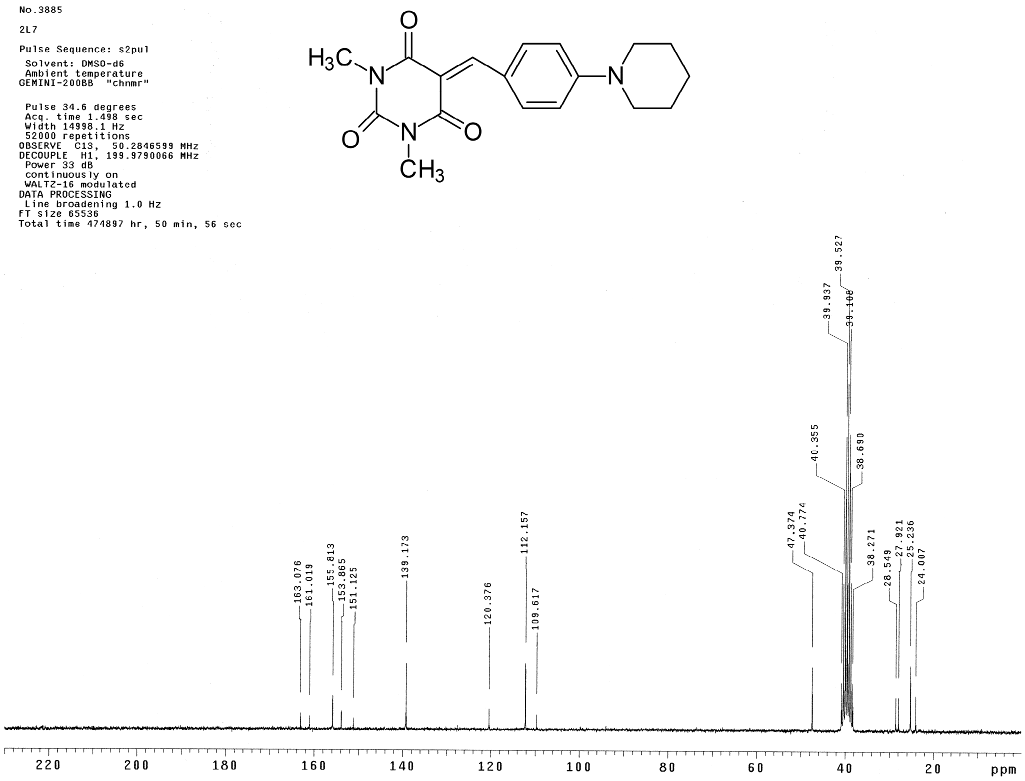

^1H NMR spectrum of **8**

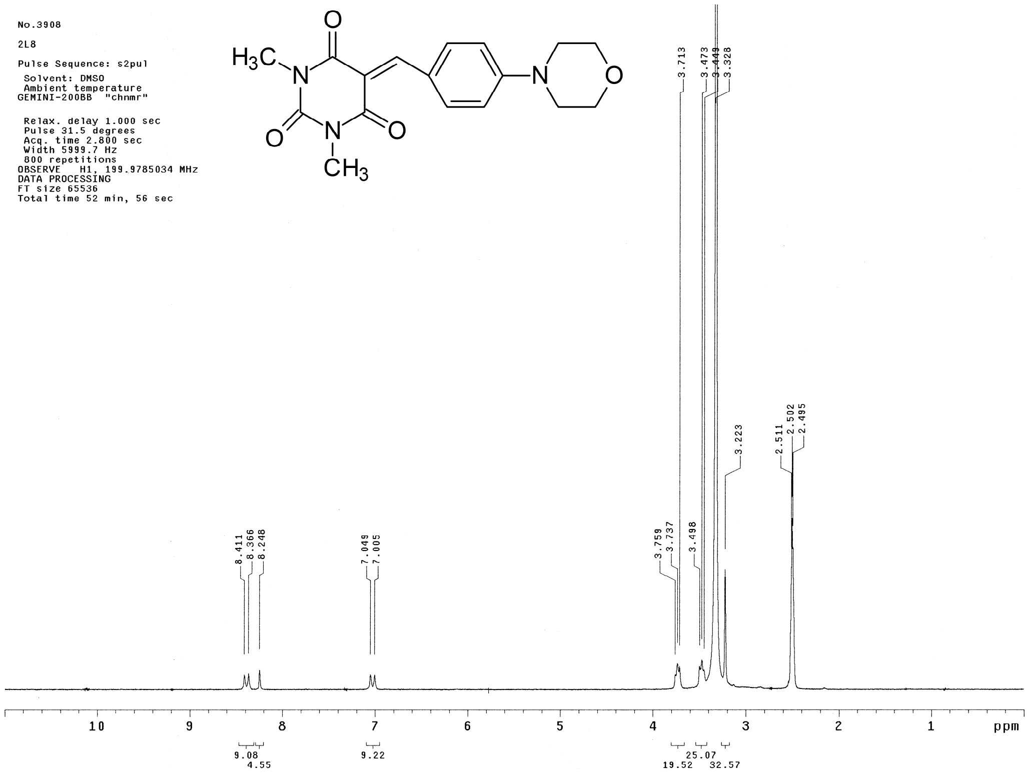

^{13}C NMR spectrum of **8**

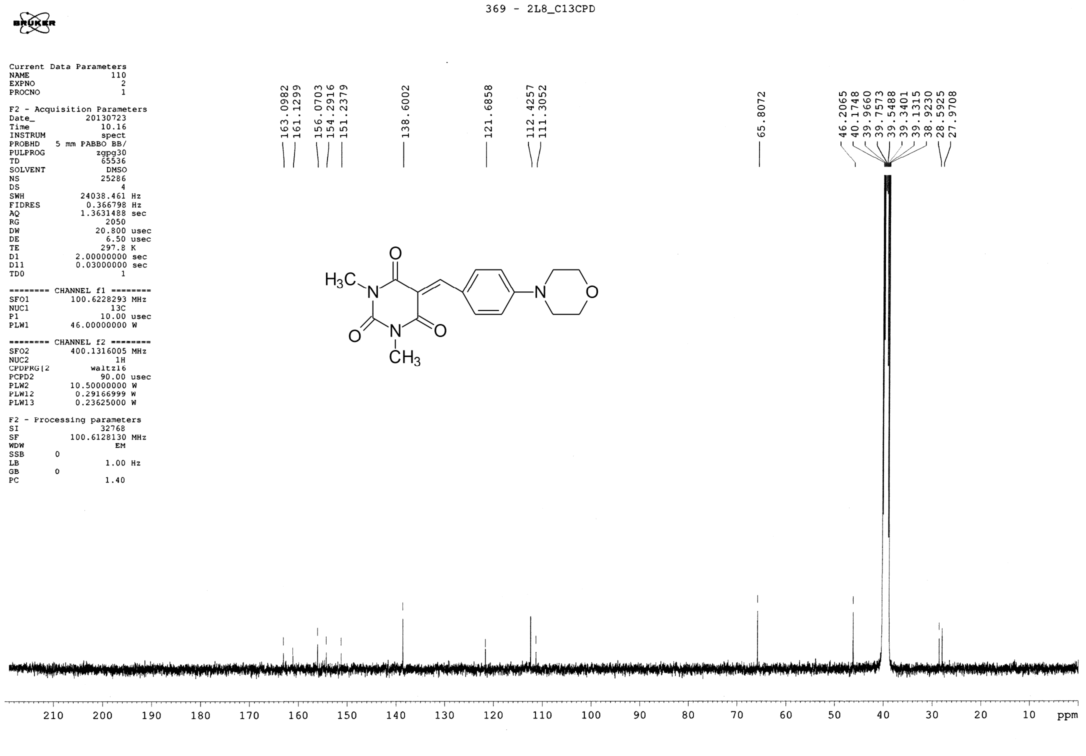

^{1}H NMR spectrum of **9**

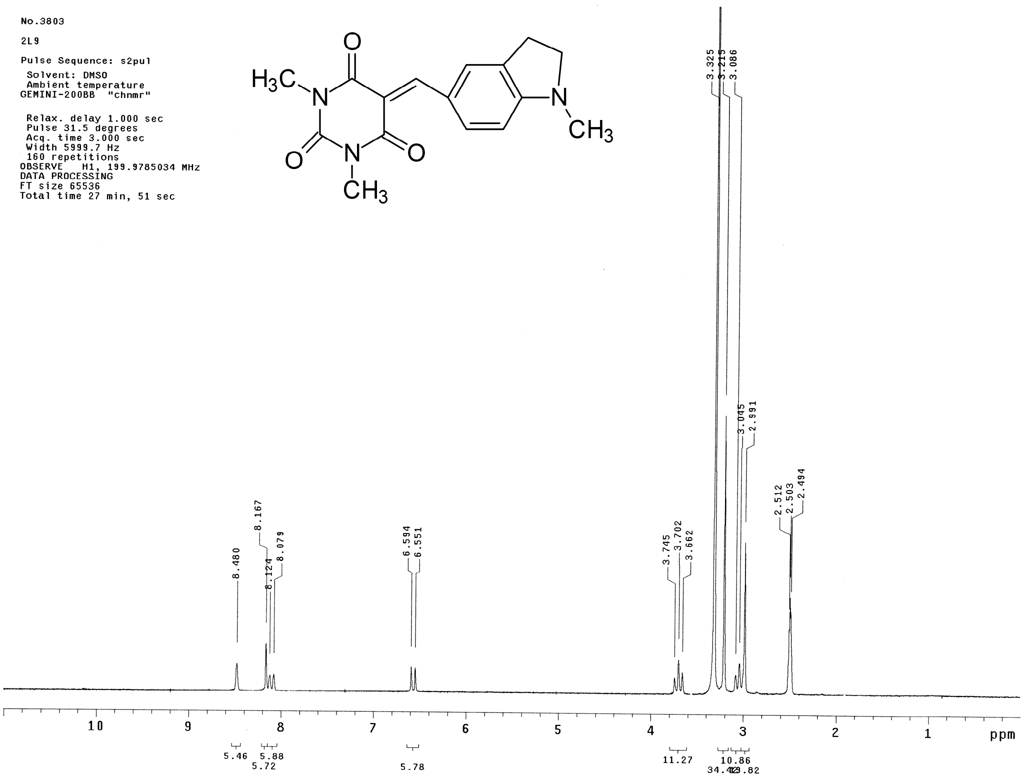

^{13}C NMR spectrum of **9**

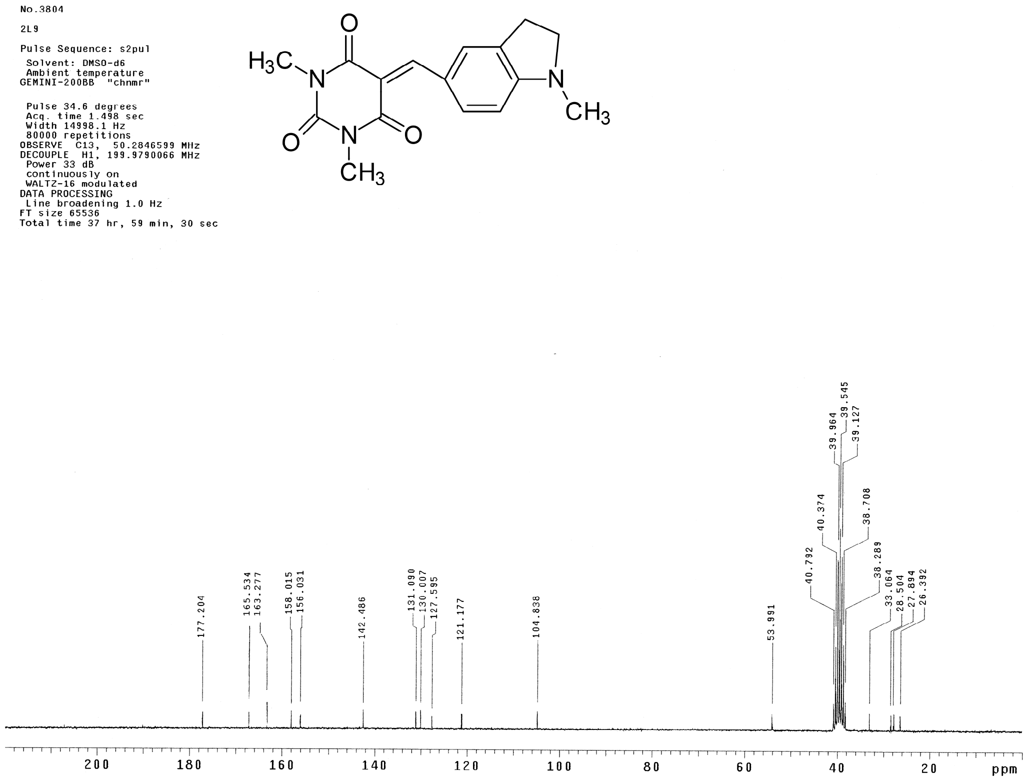

^{1}H NMR spectrum of **10**

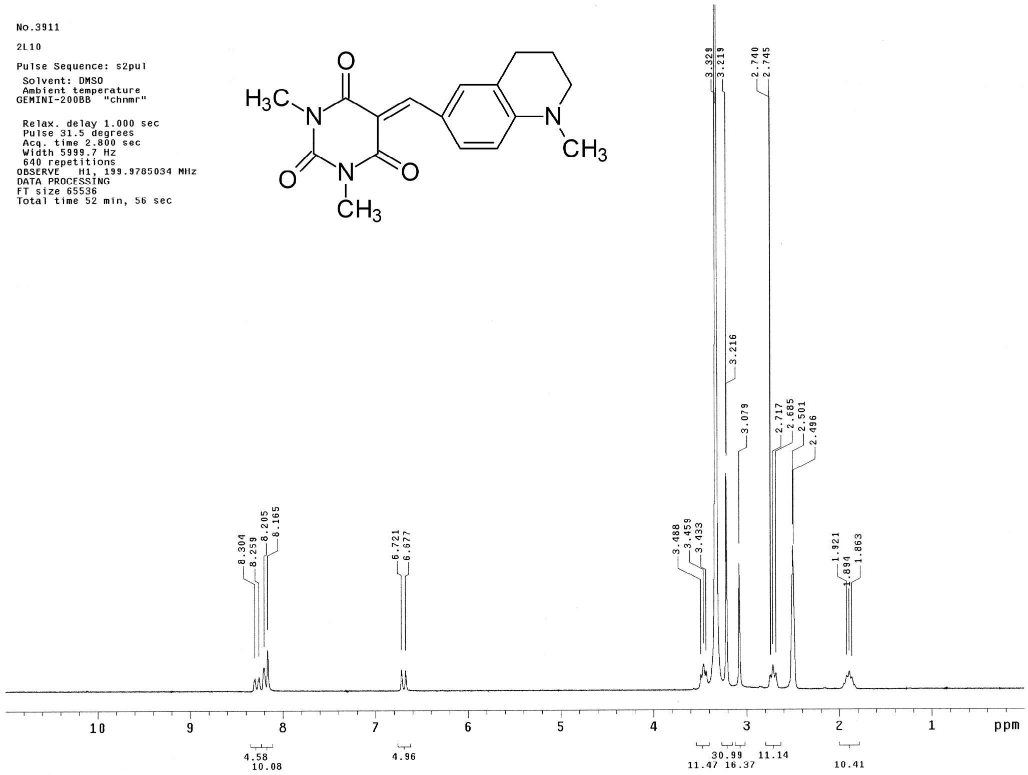

^{13}C NMR spectrum of **10**

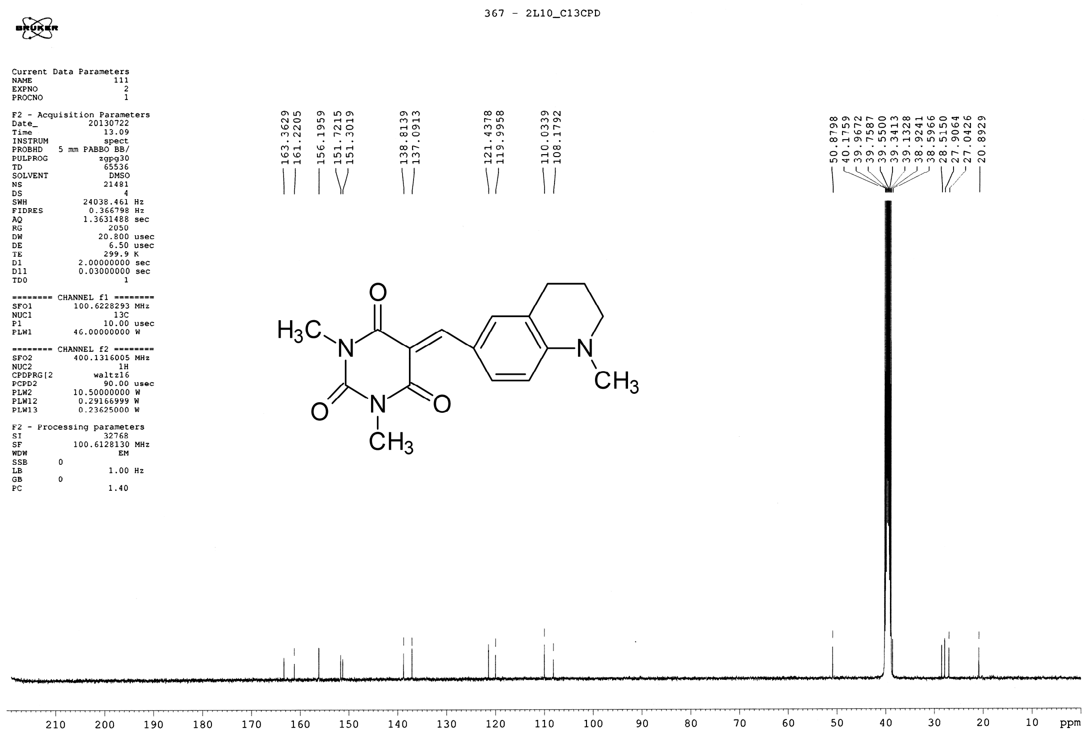

^{1}H NMR spectrum of **11**

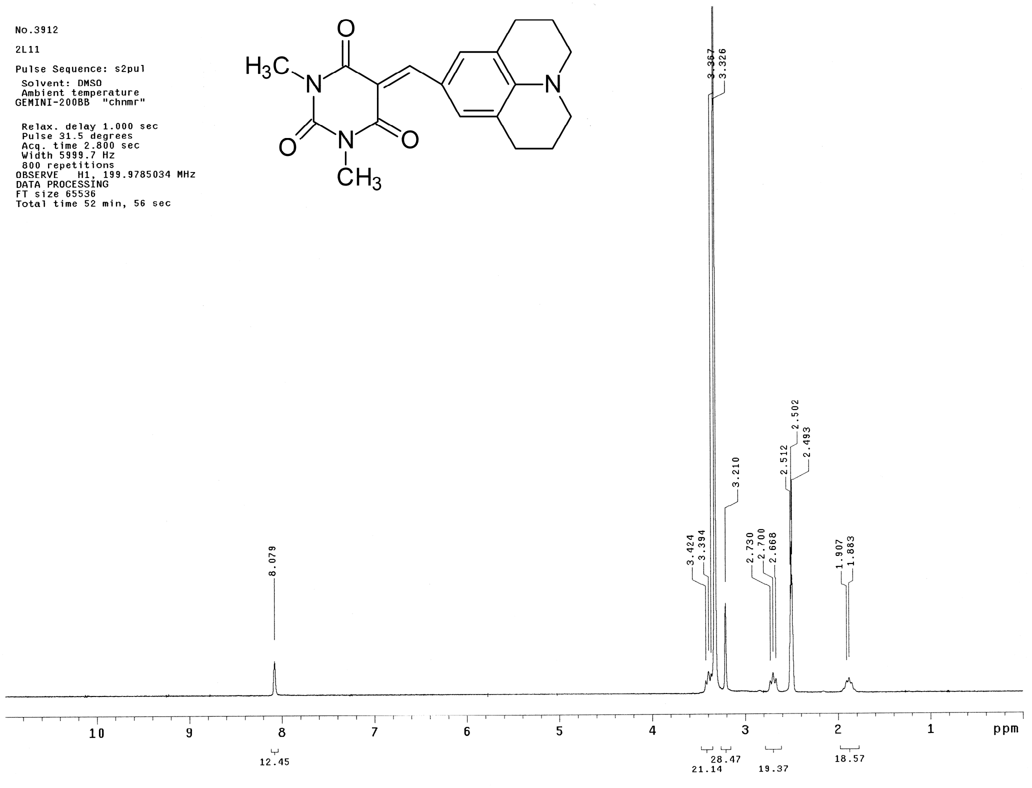

^{13}C NMR spectrum of **11**

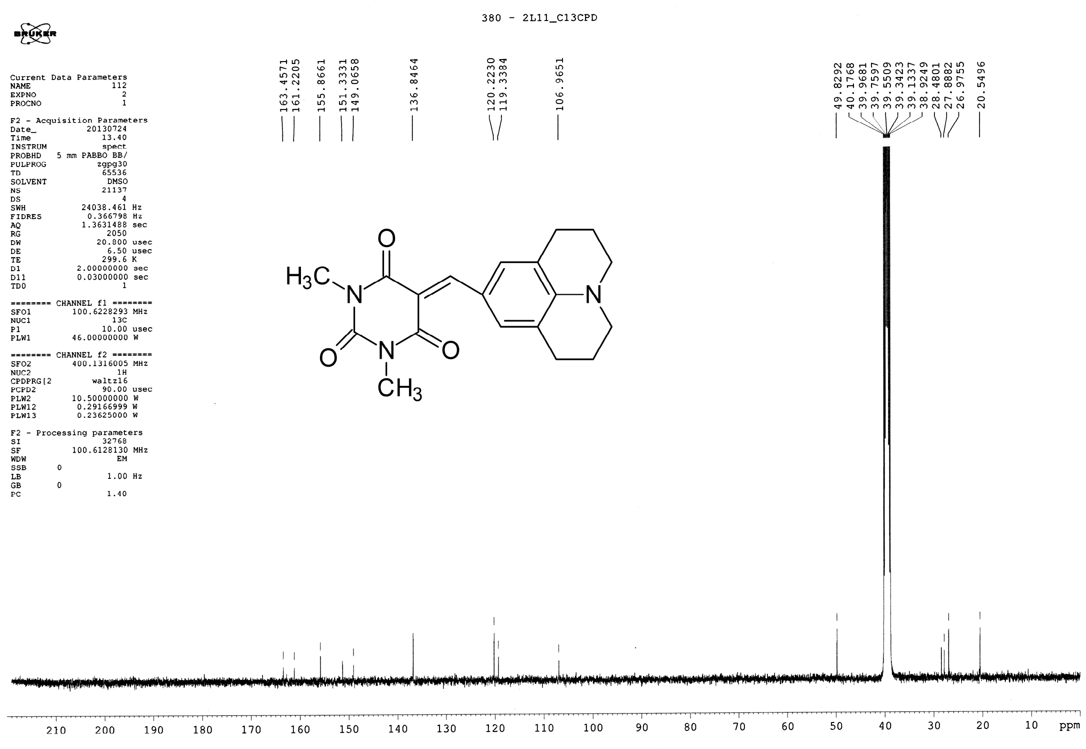

^{1}H NMR spectrum of **12**

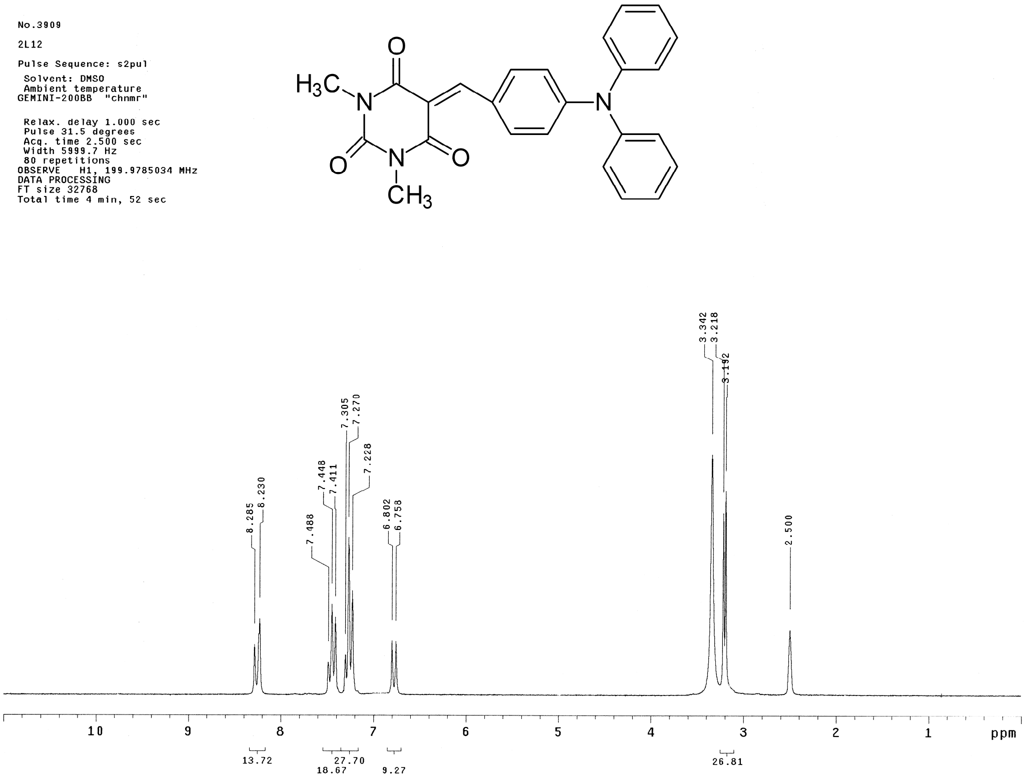

^{13}C NMR spectrum of **12**

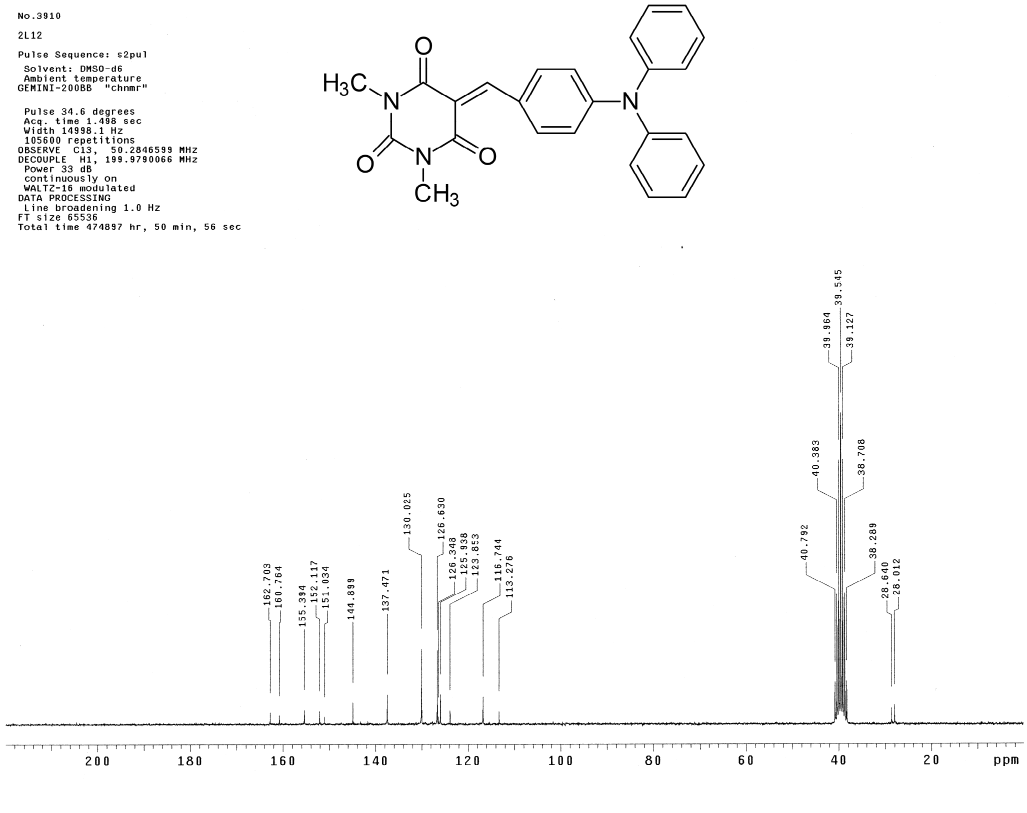

^{1}H NMR spectrum of **13**

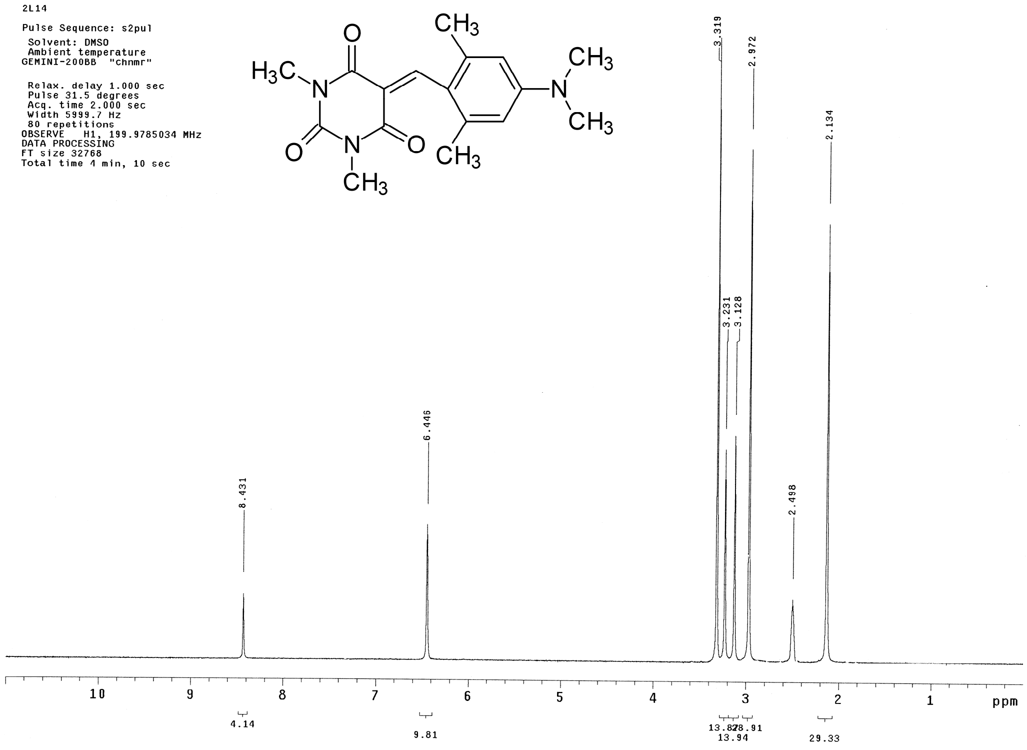

^{13}C NMR spectrum of **13**

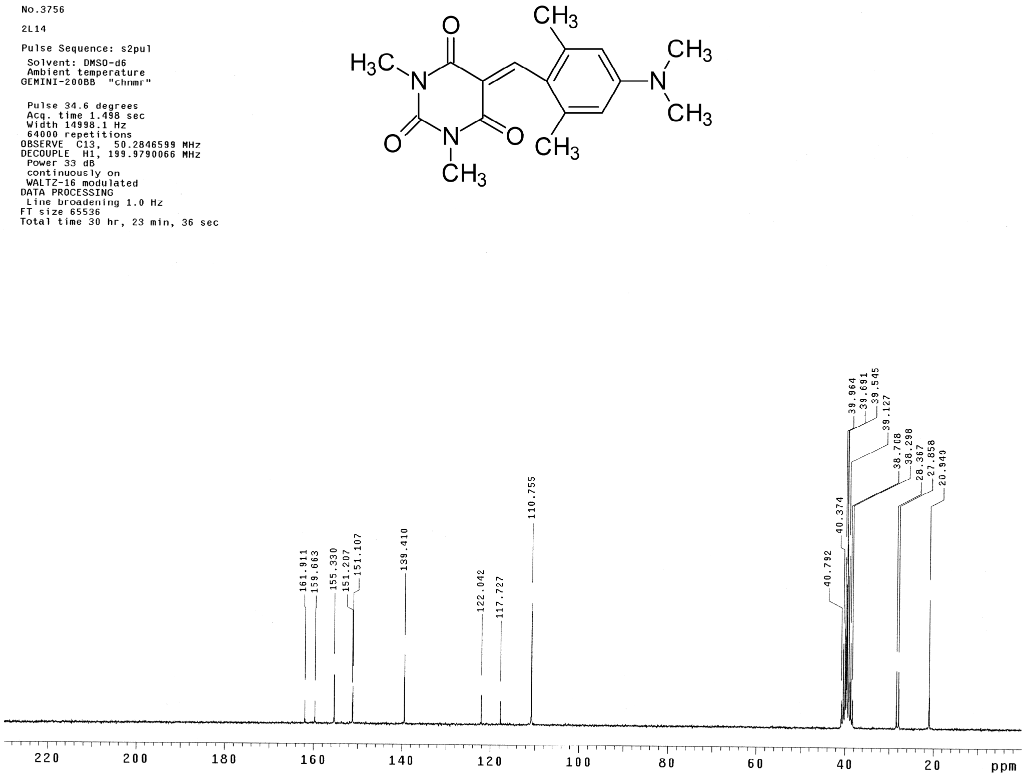

IR spectrum of **1**

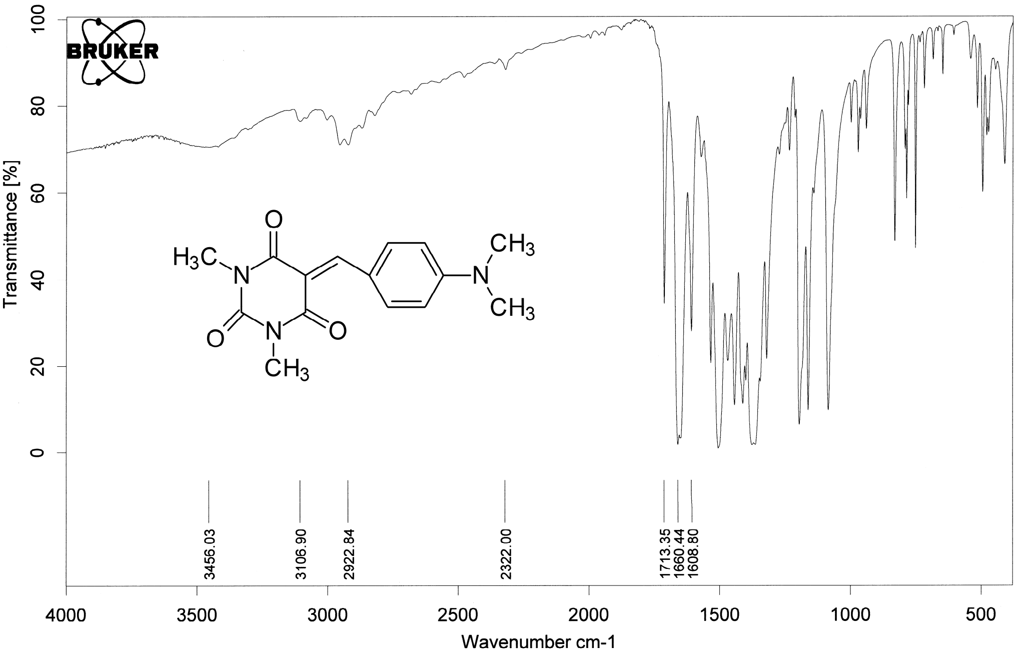

C:\Pomiary\bjed\bjed002.1 2L1 proszek w KBr	18/05/2012

Page 1/1

Enlarged spectrum in the range of 1600 - 400 cm^{-1}

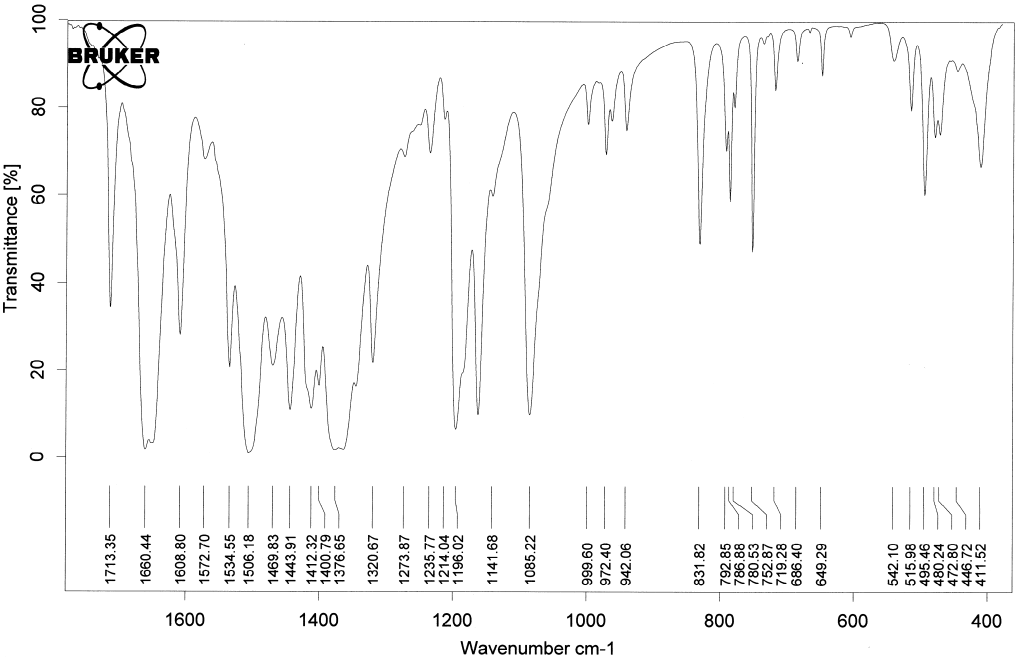

C:\Pomiary\bjed\bjed002.1 2L1 proszek w KBr	18/05/2012

Page 1/1

IR spectrum of **2**

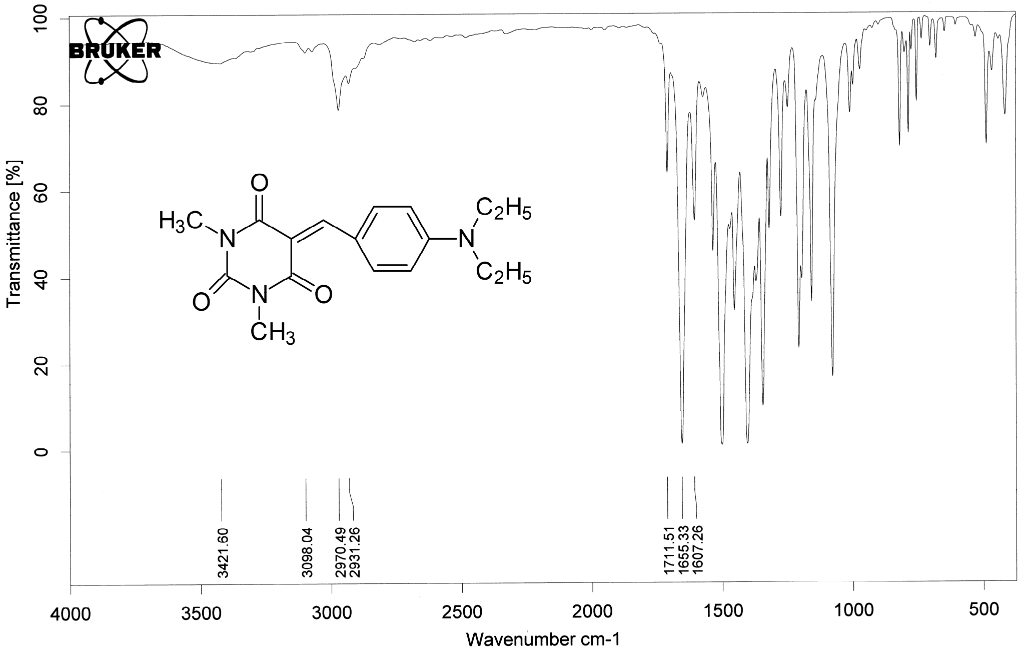

C:\Pomiary\bjed\bjed011.1 2L2 | Av. of 2 proszek w KBr	01/06/2012

Page 1/1

Enlarged spectrum in the range of 1600 - 400 cm^{-1}

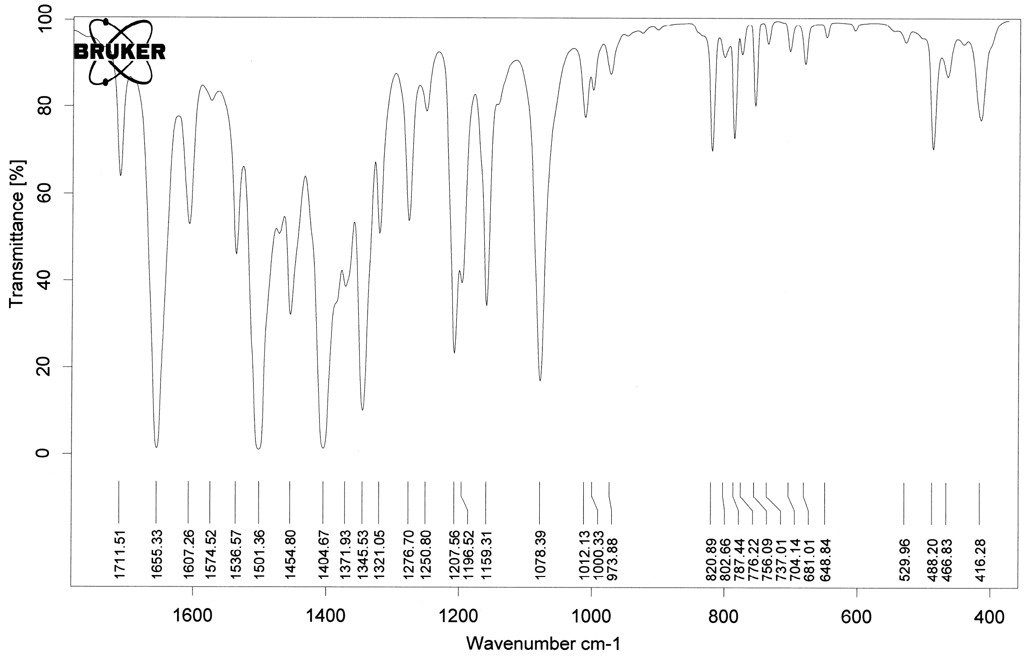

C:\Pomiary\bjed\bjed011.1 2L2 | Av. of 2 proszek w KBr	01/06/2012

Page 1/1

IR spectrum of **3**

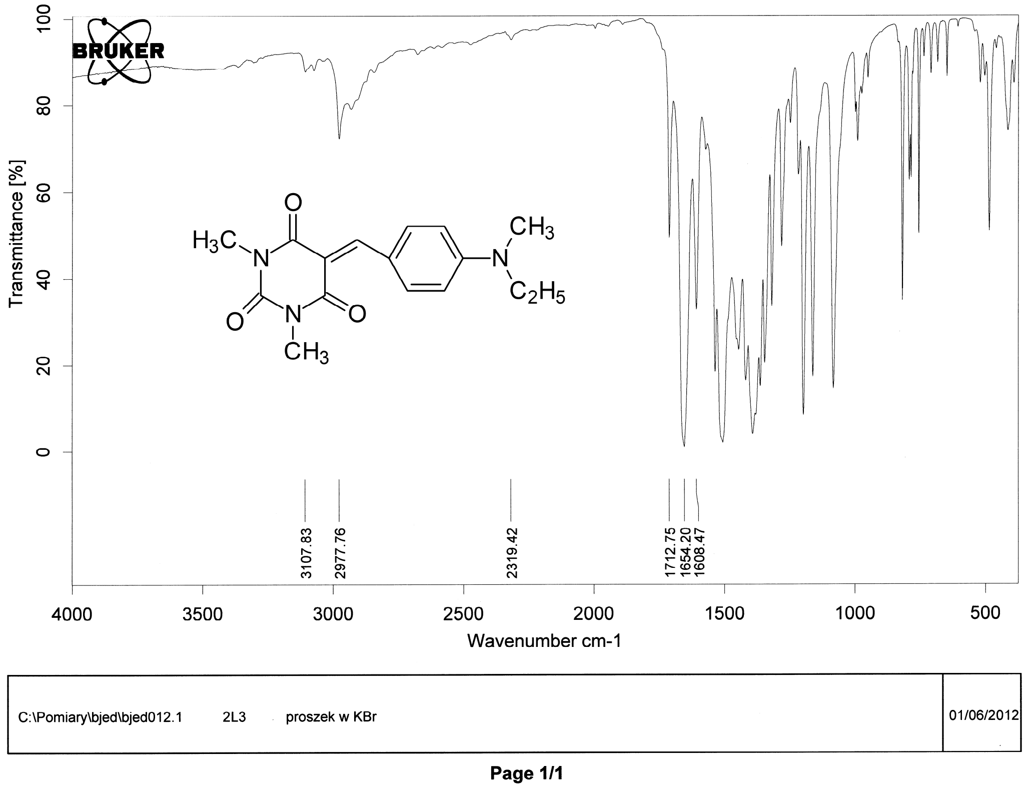

Enlarged spectrum in the range of 1600 - 400 cm^{-1}

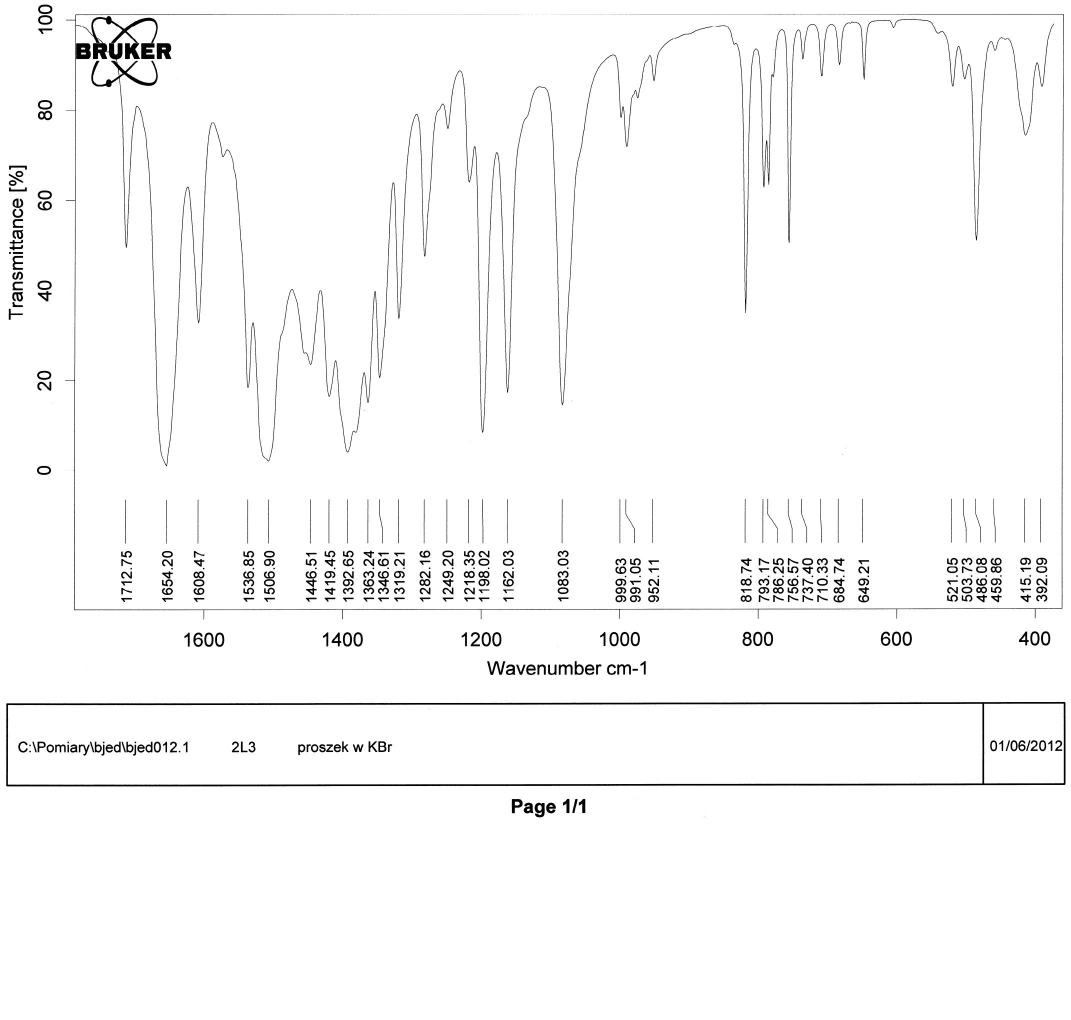

IR spectrum of **4**

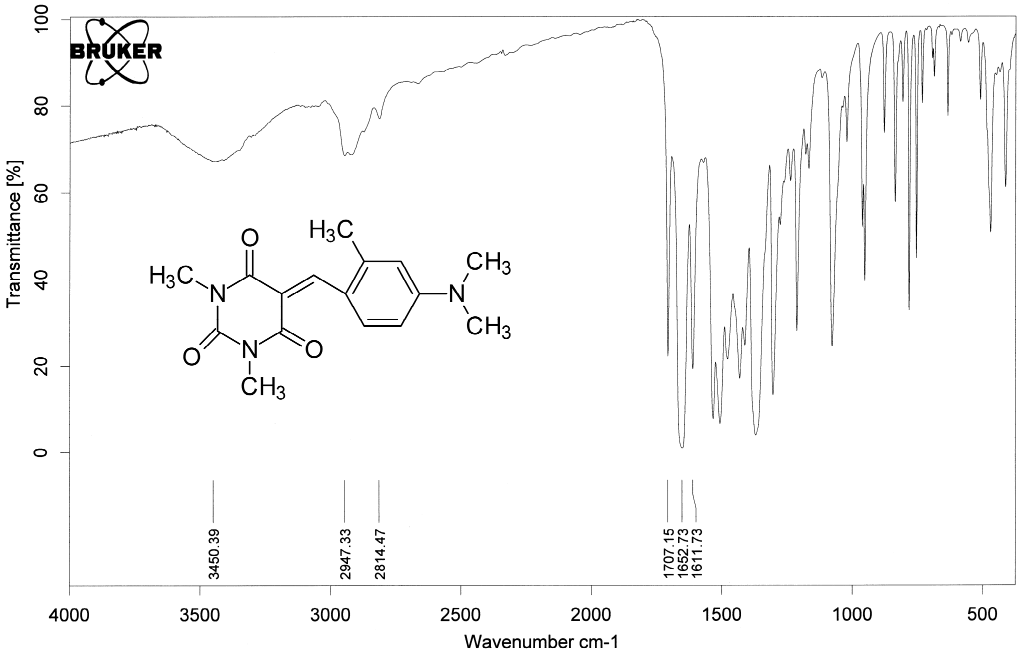

C:\Pomiary\bjed\bjed013.1 2L4 proszek w KBr 01/06/2012

Page 1/1

Enlarged spectrum in the range of 1600 - 400 cm^{-1}

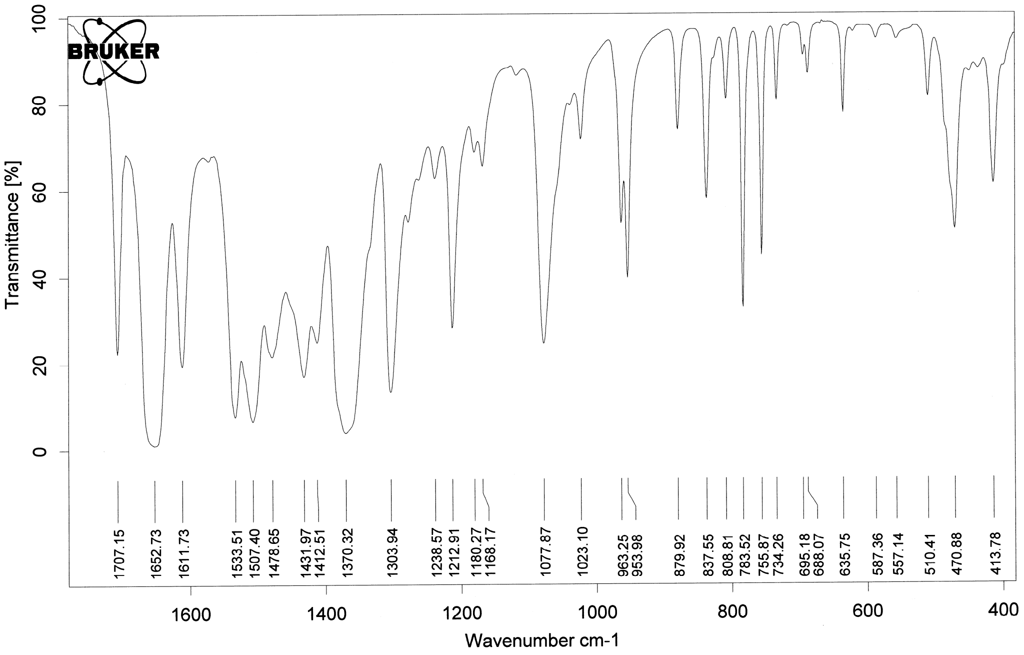

C:\Pomiary\bjed\bjed013.1 2L4 proszek w KBr 01/06/2012

Page 1/1

IR spectrum of **5**

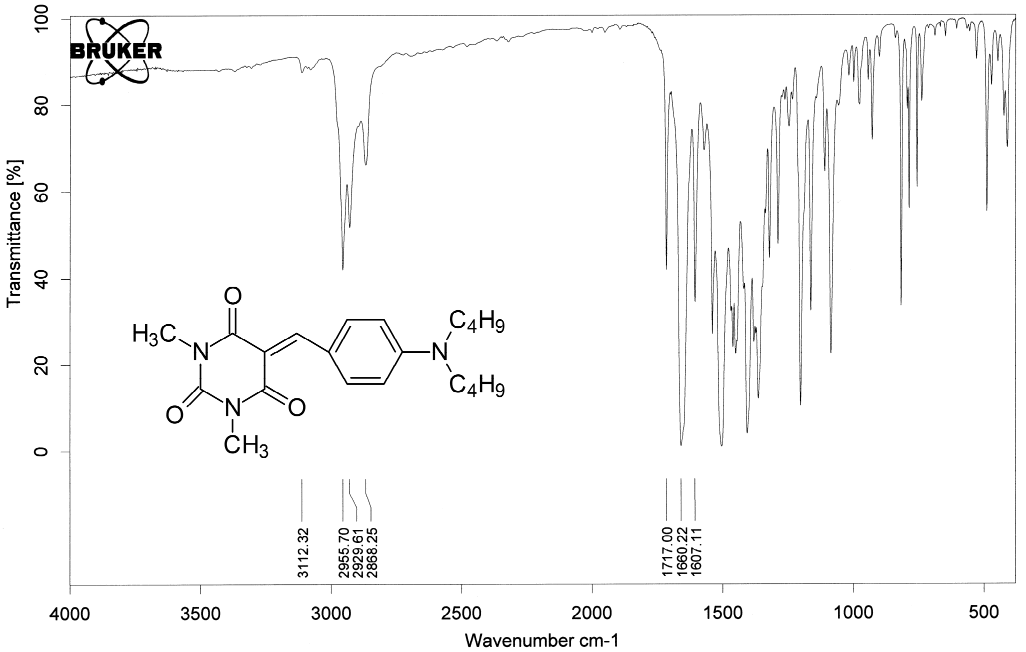

C:\Pomiary\bjed\bjed006.1 2l5 proszek w KBr	21/05/2012

Page 1/1

Enlarged spectrum in the range of 1600 - 400 cm^{-1}

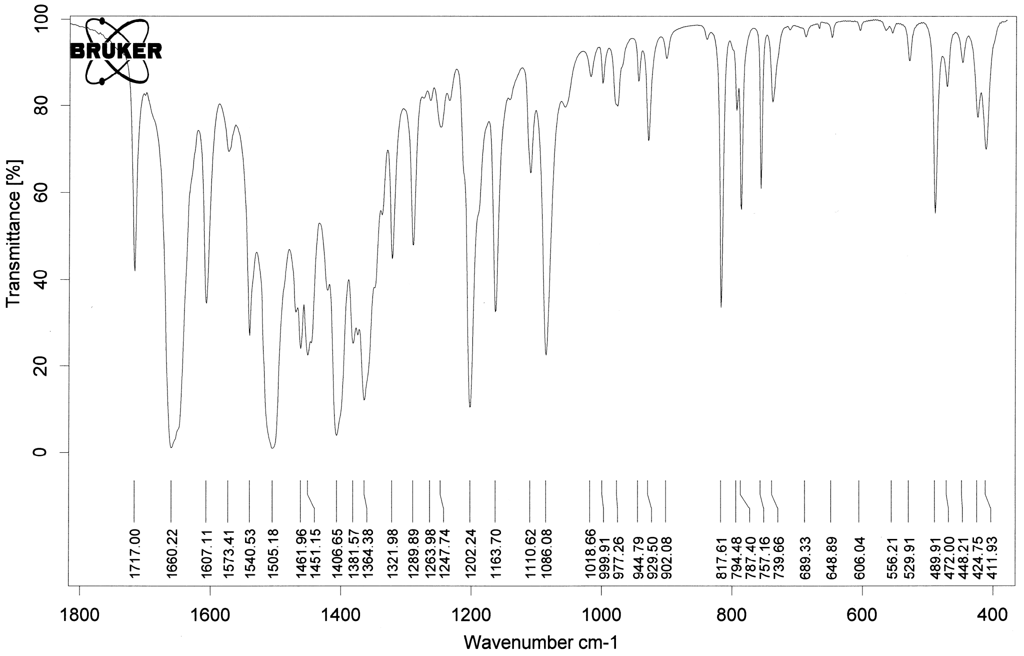

C:\Pomiary\bjed\bjed006.1 2l5 proszek w KBr	21/05/2012

Page 1/1

IR spectrum of **6**

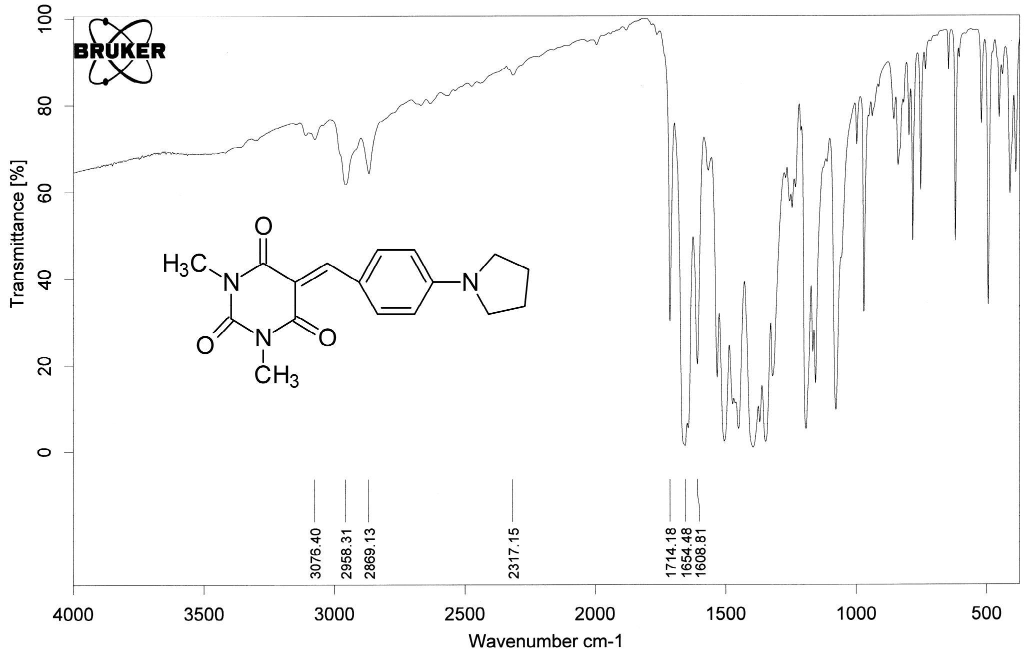

C:\Pomiary\bjed\bjed014.1	2L6	proszek w KBr	01/06/2012

Page 1/1

Enlarged spectrum in the range of 1600 - 400 cm^{-1}

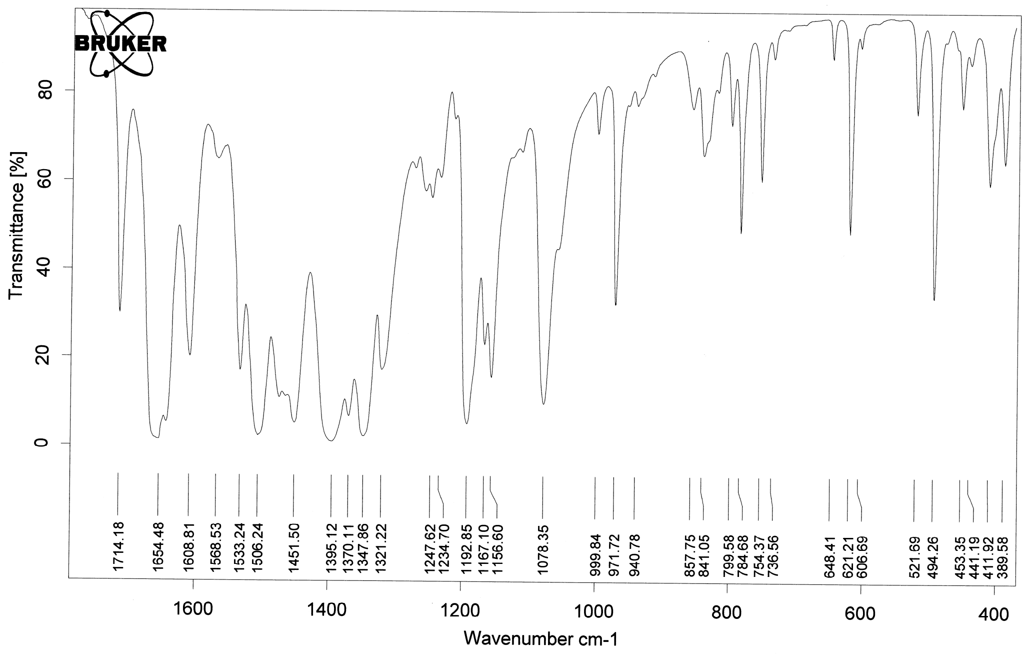

C:\Pomiary\bjed\bjed014.1	2L6	proszek w KBr	01/06/2012

Page 1/1

IR spectrum of **7**

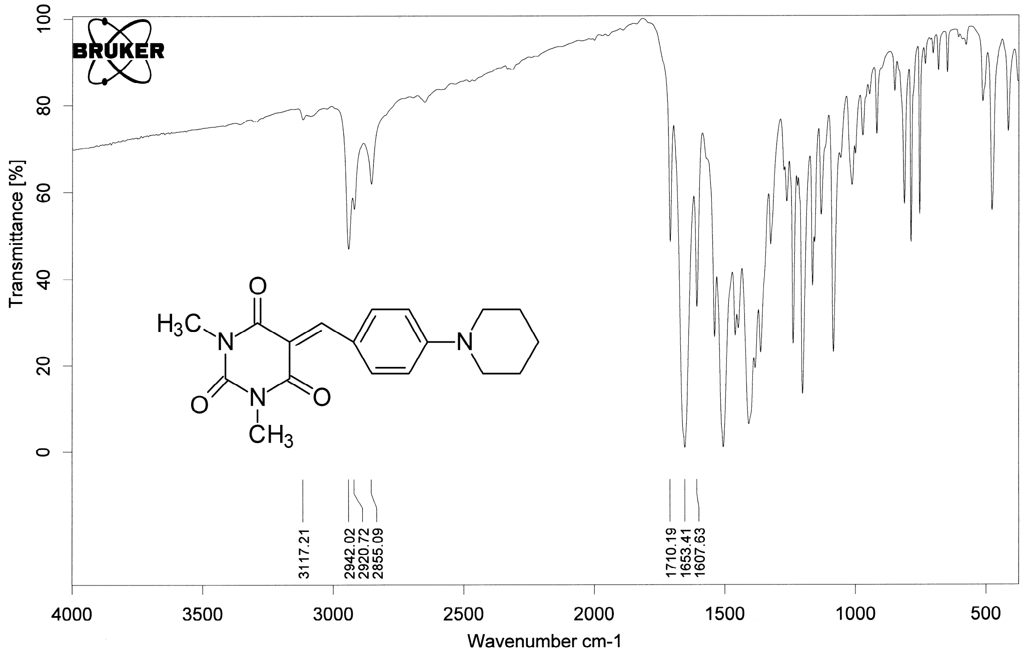

C:\Pomiary\bjed\bjed007.1	2l7	proszek w KBr	21/05/2012

Page 1/1

Enlarged spectrum in the range of 1600 - 400 cm^{-1}

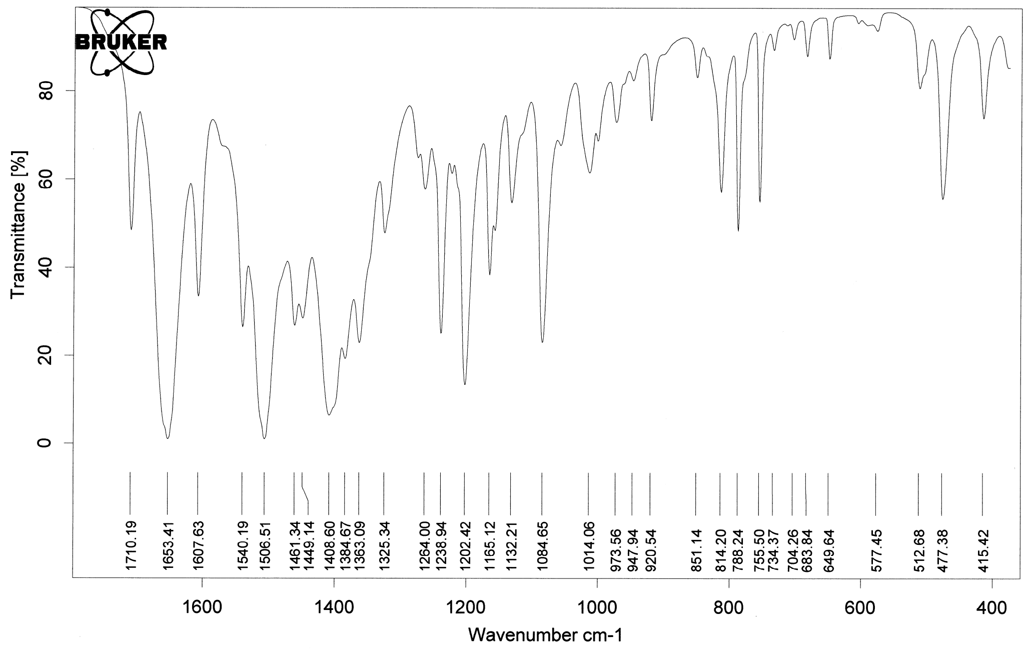

C:\Pomiary\bjed\bjed007.1	2l7	proszek w KBr	21/05/2012

Page 1/1

IR spectrum of **8**

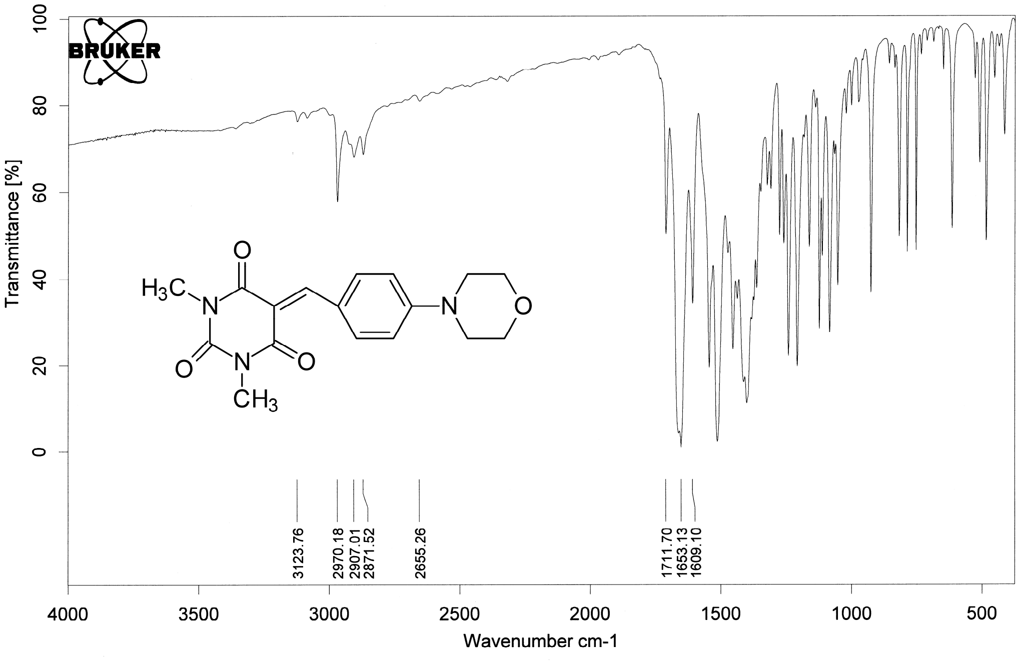

C:\Pomiary\bjed\bjed001.1	2L8	proszek w KBr	18/05/2012

Page 1/1

Enlarged spectrum in the range of 1600 - 400 cm^{-1}

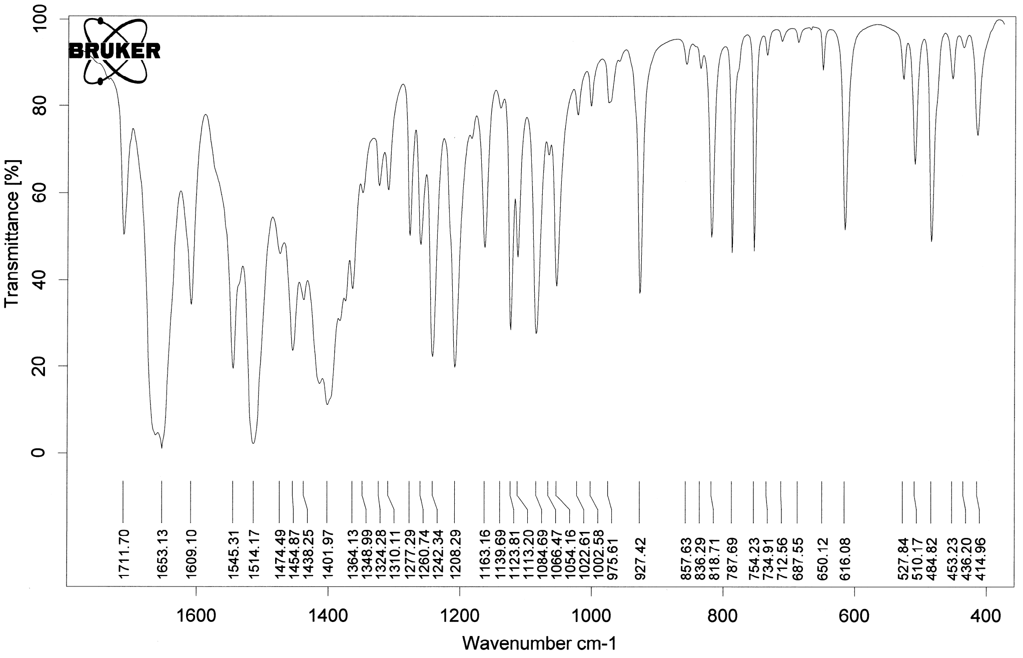

C:\Pomiary\bjed\bjed001.1	2L8	proszek w KBr	18/05/2012

Page 1/1

IR spectrum of **9**

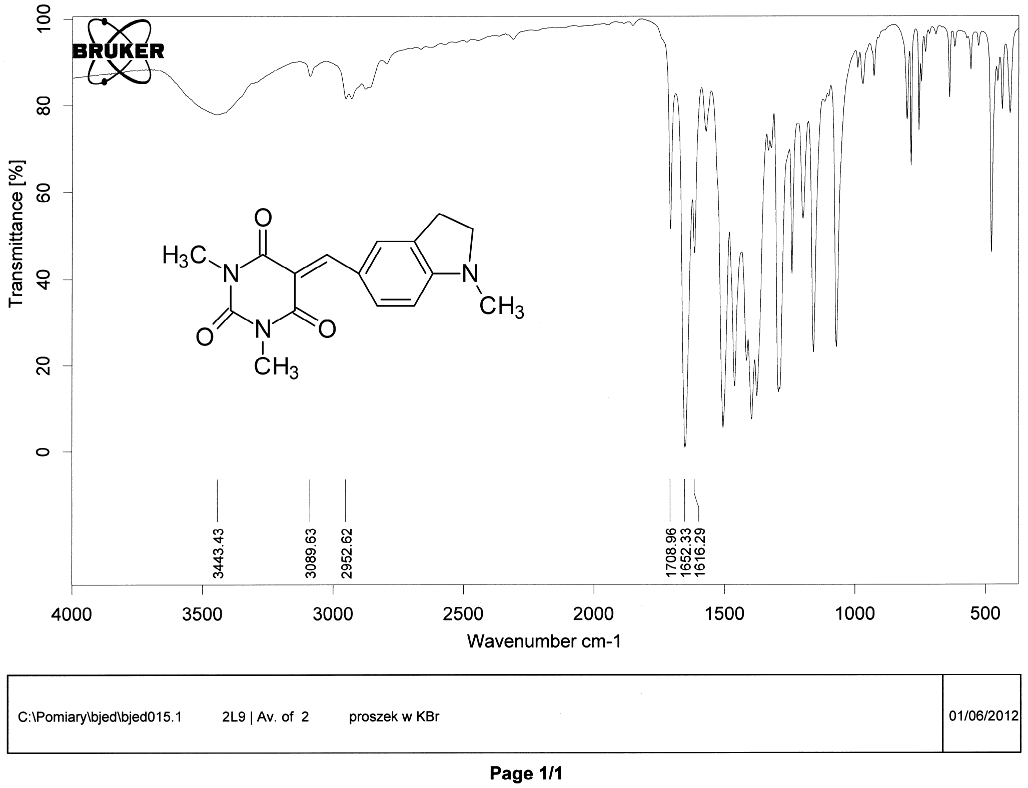

Enlarged spectrum in the range of 1600 - 400 cm^{-1}

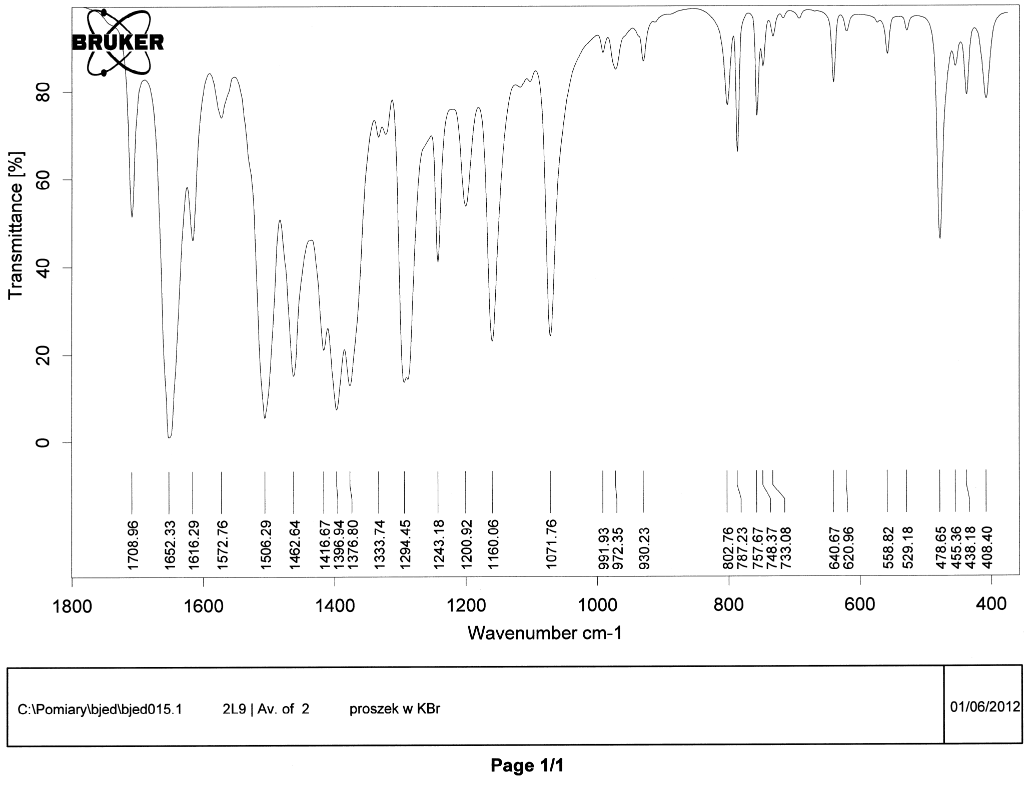

IR spectrum of **10**

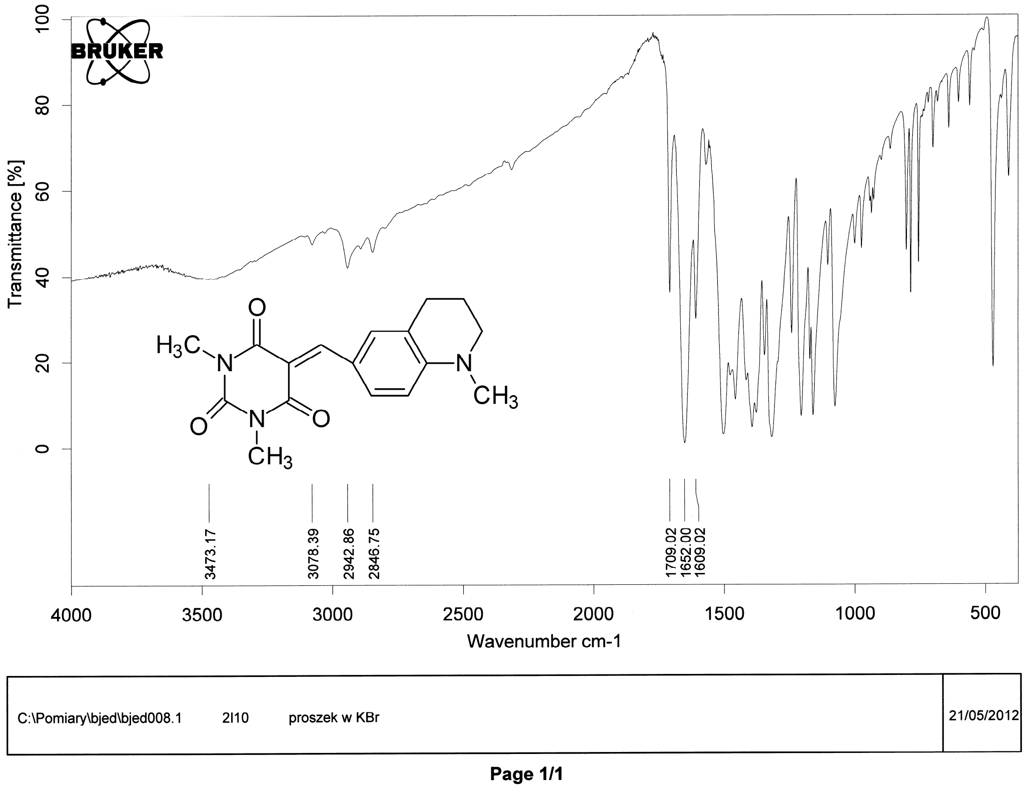

Enlarged spectrum in the range of 1600 - 400 cm^{-1}

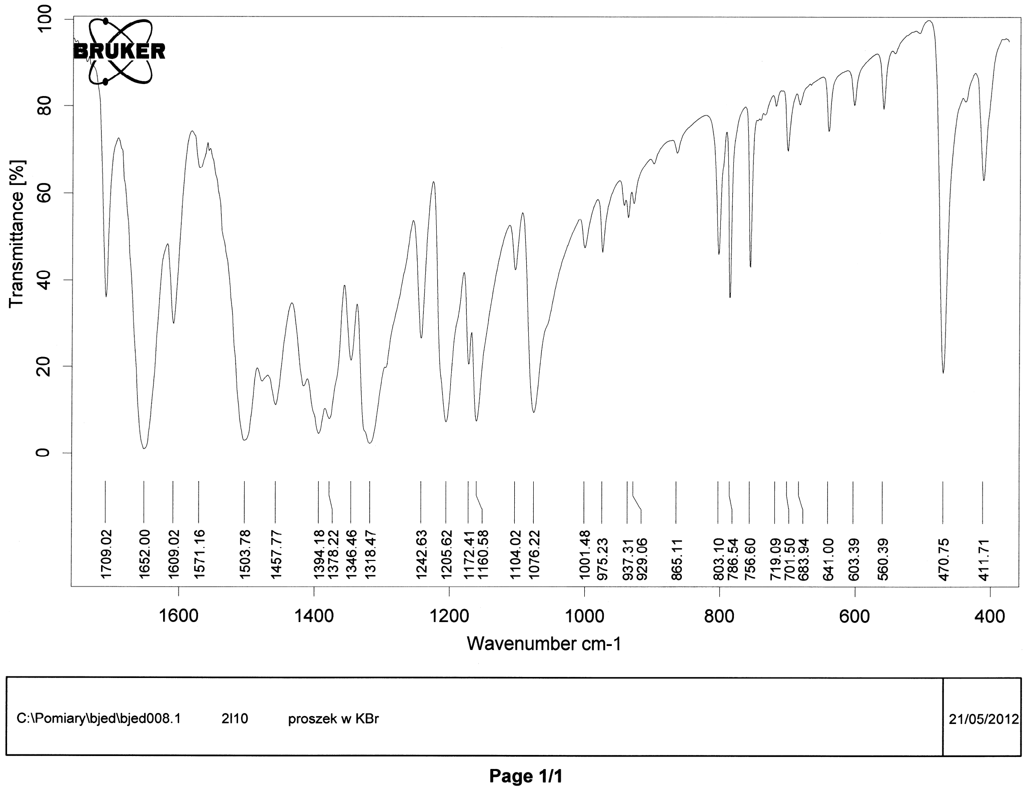

IR spectrum of **11**

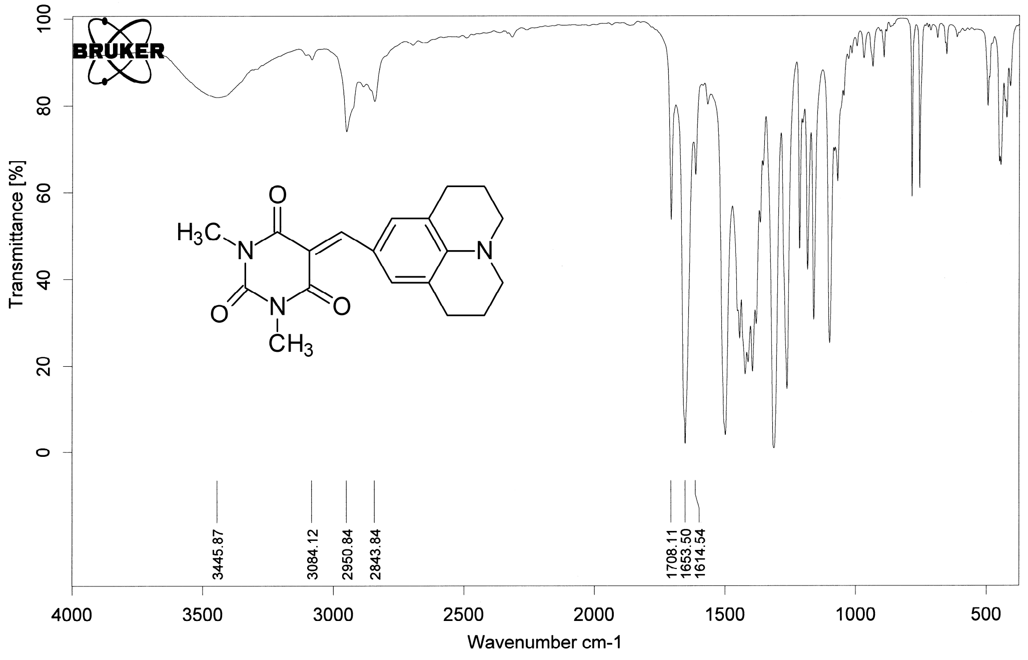

C:\Pomiary\bjed\bjed009.1 2I11 proszek w KBr	21/05/2012

Page 1/1

Enlarged spectrum in the range of 1600 - 400 cm^{-1}

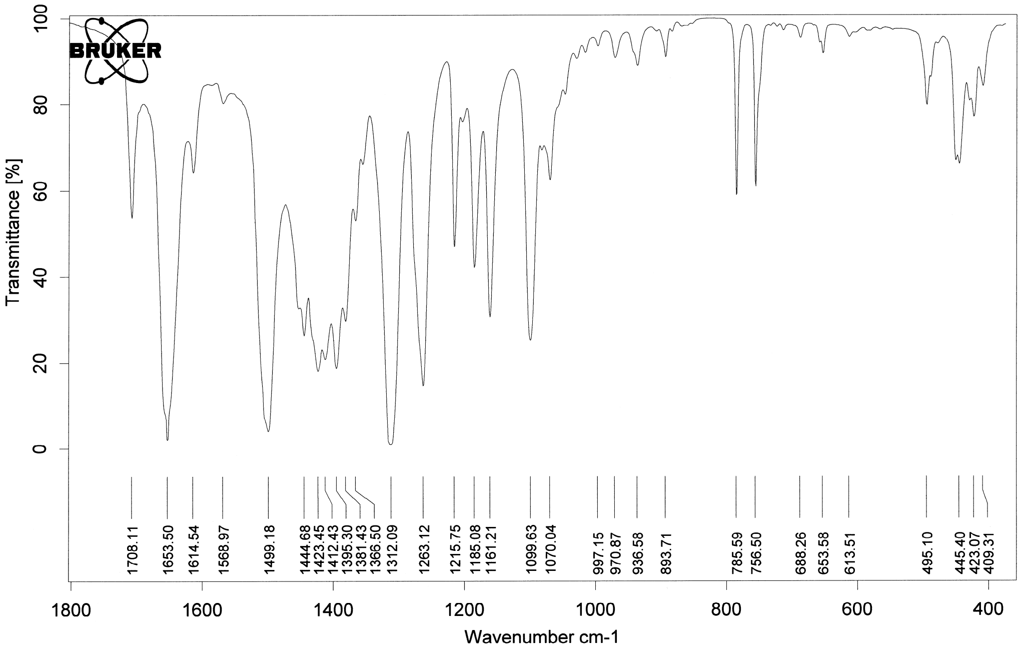

C:\Pomiary\bjed\bjed009.1 2I11 proszek w KBr	21/05/2012

Page 1/1

IR spectrum of **12**

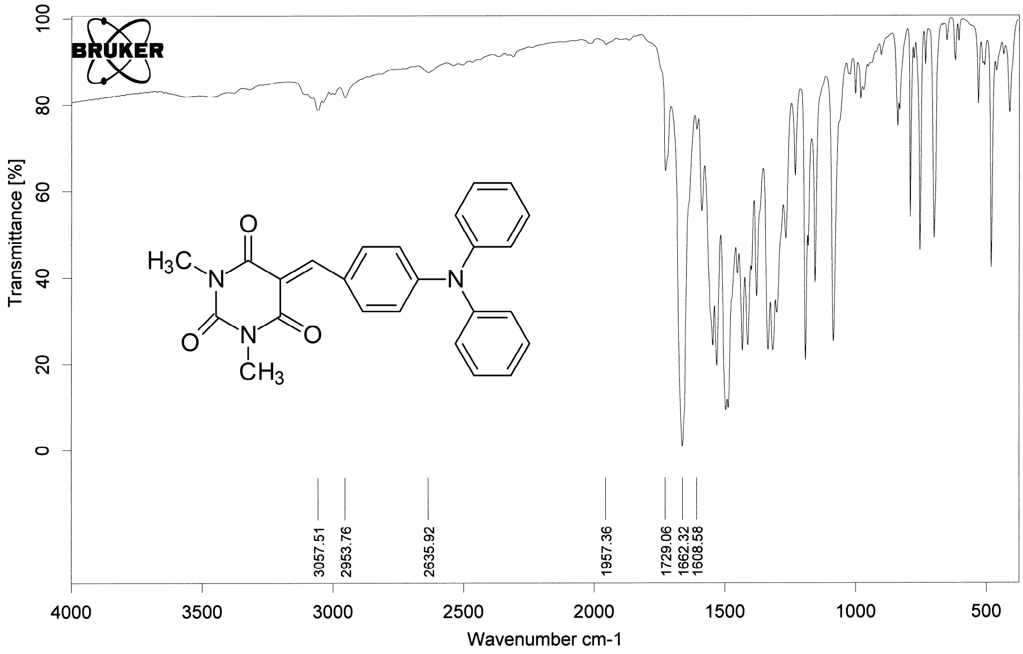

C:\Pomiary\bjed\bjed010.1	2l12	proszek w KBr		21/05/2012

Page 1/1

Enlarged spectrum in the range of 1600 - 400 cm^{-1}

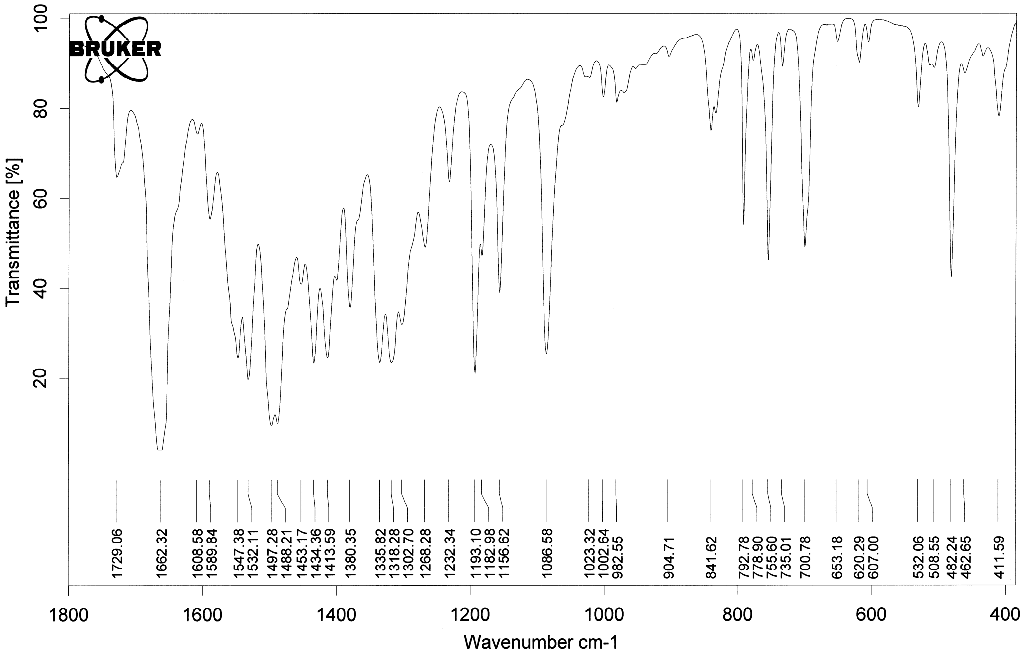

C:\Pomiary\bjed\bjed010.1	2l12	proszek w KBr		21/05/2012

Page 1/1

IR spectrum of **13**

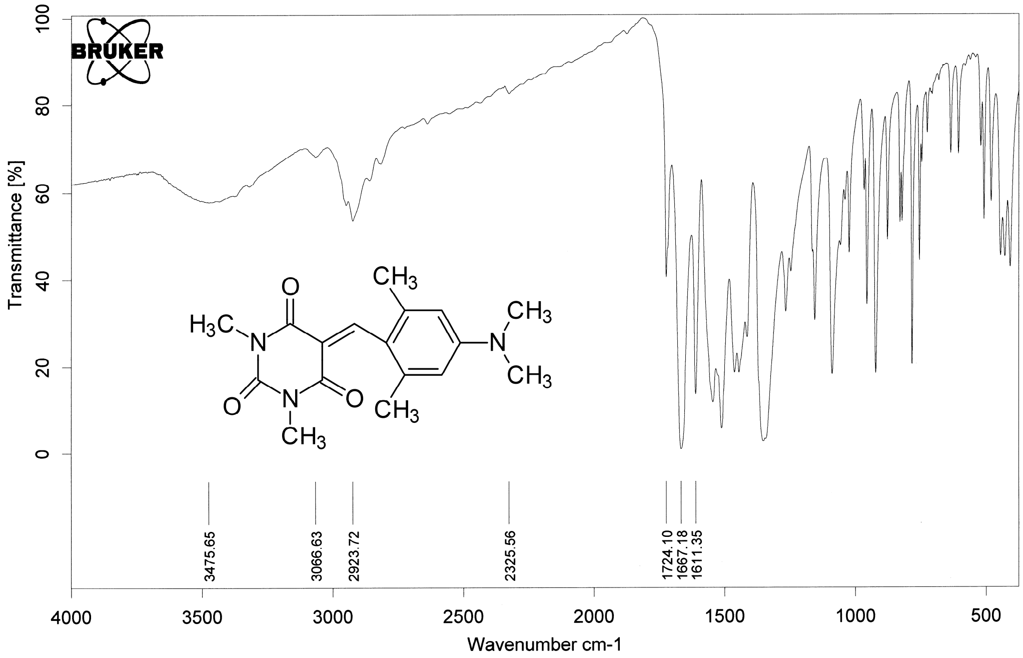

C:\Pomiary\bjed\bjed016.1 2L14 proszek w KBr	01/06/2012

Page 1/1

Enlarged spectrum in the range of 1600 - 400 cm^{-1}

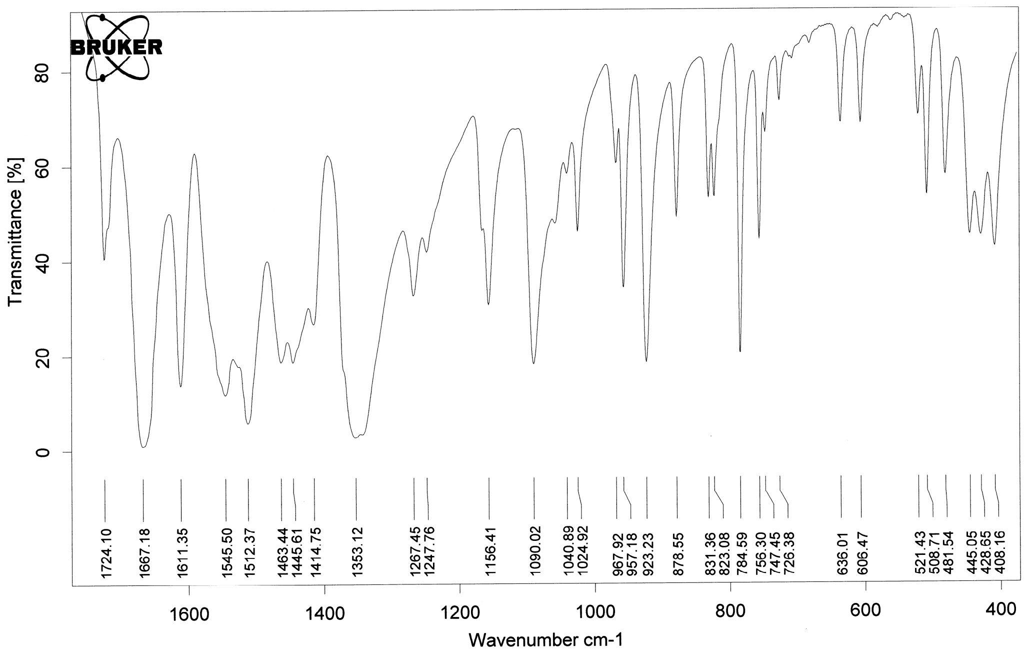

C:\Pomiary\bjed\bjed016.1 2L14 proszek w KBr	01/06/2012

Page 1/1

Table S1. Photophysical Data[a] for Compounds **1-13**

Solvent	λ^{ab}_{max}	ε_{max}	FWHM^{ab}	f_{os}	δ_{IPA}	λ^{fl}_{max}	Δν	λ^{ab}_{max}	ε_{max}	FWHM^{ab}	f_{os}	δ_{IPA}	λ^{fl}_{max}	Δν
	Compound **1**							Compound **2**						
1,4-Dx	450	7.03	2287	0.825	2.69	515	2805	455	7.20	2079	0.759	2.73	516	2598
Toluene	452	7.03	2091	0.738	2.69	516	2744	457	9.03	1916	0.951	3.45	517	2539
Et_2O	446	6.67	2200	0.742	2.55	511	2852	451.5	9.10	1991	0.910	3.48	515	2731
EtOAc	452.5	6.50	2260	0.757	2.48	521	2906	458	8.68	2016	0.884	3.31	525	2786
THF	453.5	7.15	2204	0.818	2.70	523	2930	459	8.95	2384	0.946	3.42	526	2775
MeAc	456.5	6.79	2299	0.803	2.59	531	3073	461.5	7.72	2123	0.842	2.95	532	2871
MeCN	458	6.66	2304	0.802	2.56	535	3142	464.5	8.43	2170	0.943	3.22	539	2976
DMF	463.5	7.17	2271	0.841	2.71	543	3159	469	7.93	2102	0.861	3.03	542	2872
DMSO	468.5	6.50	2687	0.776	2.48	547	3063	472.5	7.11	2107	0.782	2.72	548	2916
	Compound **3**							Compound **4**						
1,4-Dx	452.5	7.46	2179	0.819	2.82	520	2869	462.5	5.61	2388	0.688	2.14	537	3000
Toluene	454.5	7.89	2011	0.793	3.00	522	2845	464	5.99	2214	0.672	2.29	539	2999
Et_2O	448.5	8.14	2083	0.860	3.11	518	2992	458.5	5.52	2268	0.632	2.08	530	2942
EtOAc	455	7.75	2165	0.864	2.96	527	3003	465.5	5.85	2323	0.701	2.23	539	2929
THF	455	7.58	2142	0.839	2.89	529	3074	466.5	5.87	2295	0.697	2.24	541	2952
MeAc	459	7.93	2218	0.904	3.03	538	3199	469.5	5.96	2383	0.733	2.27	543	2883
MeCN	462	8.06	2212	0.927	3.08	541	3161	472.5	6.04	2408	0.757	2.31	547	2882
DMF	465	7.03	2187	0.796	2.69	545	3157	477.5	5.69	2326	0.680	2.16	554	2892
DMSO	470.5	6.87	2198	0.789	2.62	553	3171	482	5.72	2356	0.692	2.19	560	2890
	Compound **5**							Compound **6**						
1,4-Dx	458	8.55	2051	0.886	3.26	524	2750	455.5	8.02	2096	0.852	3.06	524	2870

Toluene	459	8.10	1909	0,773	3.09	524	2703	459	7.83	1936	0.749	2.99	528	2847
Et_2O	454	8.56	1960	0.841	3.27	523	2906	452	6.85	2037	0.688	2.61	520	2893
EtOAc	460.5	8.32	2044	0.870	3.18	532	2919	458.5	7.62	2125	0.819	2.91	529	2907
THF	461.5	8.43	2005	0.873	3.22	534	2942	460	7.68	2039	0.804	2.87	531	2907
MeAc	464.5	8.52	2095	0.921	3.25	539	2976	462.5	7.78	2142	0.858	2.96	535	2930
MeCN	467	8.11	2102	0.881	3.10	544	3031	465	7.63	2146	0.848	2.91	538	2918
DMF	471	7.44	2082	0.794	2.81	548	2983	468.5	7.36	2126	0.805	2.81	544	2962
DMSO	475	7.60	2101	0.824	2.90	554	3002	471	7.40	2143	0.817	2.83	547	2950
	Compound **7**							Compound **8**						
1,4-Dx	453.5	5.73	2480	0.736	2.19	530	3183	439	5.02	2904	0.786	1.92	515	3388
Toluene	456.5	5.87	2301	0.683	2.24	532	3109	441	5.15	2682	0.737	1.97	518	3345
Et_2O	450.5	6.32	2404	0.781	2.41	533	3436	436	-	2815	-	-	516	3582
EtOAc	457	6.18	2500	0.794	2.33	545	3533	442	5.06	2912	0.809	1.93	524	3540
THF	458	6.42	2896	0.824	2.45	545	3485	443	4.82	2822	0.734	1.84	527	3573
MeAc	460.5	5.91	2481	0.761	2.25	543	3299	447	5.42	2979	0.858	2.05	535	3705
MeCN	464	6.48	2484	0.838	2.47	543	3136	447	5.06	2987	0.802	1.93	537	3774
DMF	468	5.66	2434	0.714	2.16	548	3119	453	4.96	2928	0.782	1.90	543	3635
DMSO	472.5	5.53	2414	0.686	2.10	553	3081	458	4.51	2932	0.701	1.72	551	3685
	Compound **9**							Compound **10**						
1,4-Dx	464.5	6.72	2678	0.776	2.57	531	2696	462.5	7.59	2046	0.777	2.87	524	2538
Toluene	468	6.93	2123	0.752	2.65	535	2676	464.5	7.81	1900	0.736	2.99	525	2481
Et_2O	460	-	2204	-	-	524	2655	458	6.90	2002	0.700	2.64	521	2640
EtOAc	469	6.12	2226	0.711	2.34	534	2630	465	7.87	2073	0.835	3.01	532	2708
THF	469.5	6.42	2190	0.733	2.45	535	2608	466.5	7.72	2023	0.805	2.95	533	2675
MeAc	473.5	6.72	2237	0.810	2.66	538	2532	470	7.55	2088	0.811	2.87	542	2826

MeCN	477.5	6.92	2224	0.799	2.67	543	2526	473.5	7.79	2078	0.839	2.98	547	2838
DMF	482	6.82	2169	0.767	2.61	550	2565	477	7.65	2064	0.817	2.92	552	2848
DMSO	487	6.76	2151	0.752	2.58	555	2516	482	6.23	2054	0.649	2.38	558	2826
Compound **11**								Compound **12**						
1,4-Dx	474	8.31	1838	0.765	3.17	535	2405	459	3.18	2832	0.481	3.18	594	4951
Toluene	476	9.33	1730	0.793	3.57	537	2386	463.5	3.67	2644	0.517	1.40	583	4422
Et_2O	470.5	8.78	1770	0.776	3.35	532	2457	456.5	4.42	2701	0.637	1.69	597	5155
EtOAc	477	9.33	1852	0.873	3.56	541	2480	456	4.06	2853	0.645	1.55	642	6354
THF	479	8.88	1772	0.809	3.37	544	2494	458	2.59	2818	0.392	0.98	644	6306
MeAc	482.5	8.58	1895	0.833	3.28	549	2510	456	3.69	2959	0.580	1.41	685	7331
MeCN	486	9.01	1873	0.872	3.50	554	2526	458	3.66	2970	0.577	1.40	686	7257
DMF	489.5	8.60	1859	0.819	3.28	558	2508	460	3.05	2977	0.480	1.16	680	7033
DMSO	494	8.35	1856	0.799	3.19	563	2481	462.5	3.49	3021	0.558	1.32	684	7002
Compound **13**														
1,4-Dx	469	2.15	3225	0.371	0.82	526	2311							
Toluene	476	2.27	2877	0.345	0.87	531	2176							
Et_2O	469.5	2.21	2995	0.354	0.84	534	2573							
EtOAc	471	2.06	3230	0.357	0.79	542	2781							
THF	473	1.94	3744	0.326	0.73	544	2759							
MeAc	473	1.95	3371	0.351	1.94	546	2827							
MeCN	477	1.73	3427	0.318	0.66	557	3011							
DMF	481	1.73	3450	0.321	0.66	560	2933							
DMSO	484	1.86	3402	0.327	0.71	567	3024							

[a]Absorption ($\lambda^{ab}_{max}; nm$), fluorescence maxima ($\lambda^{fl}_{max}; nm$), shift ($\Delta\nu$; cm^{-1}), maximum extinction coefficient (ε_{max}; 10^4 $M^{-1}cm^{-1}$), full width at half maximum (FWHM; cm^{-1}), one-photon absorption cross-section (δ_{IPA}; $\AA^2$) and oscillator strength (f_{os}).

Table S2. Estimated from eq. (13), coefficients (y_0, a_{SP}, b_{SdP}, c_{SA}, and d_{SB}), their standard errors and correlation coefficients (R^2) for the multiple linear regression analysis of ν_{Ab}, ν_{Fl} and $\Delta\nu^{SS}$ of **1-13** in 9 solvents as a function of the Catalán four-parameter solvent scale

y	y_0	a_{SP}	b_{SdP}	c_{SA}	d_{SB}	R^2
			Compound **1**			
ν_{ab}	24068±155	**-(2181±200)**	-(798±69)	-(1491±779)	-(9±78)	0.989
ν_{fl}	21269±201	-(1943±258)	**-(1295±89)**	-(1497±1006)	-(2±100)	0.991
$\Delta\nu^{SS}$	2801±194	-(238±250)	497±86	6±974	-(7±97)	0.920
			Compound **2**			
ν_{ab}	23568±169	**-(1862±218)**	-(815±75)	-(1297±850)	56±85	0.986
ν_{fl}	20584±211	-(1150±271)	**-(1170±93)**	-(2693±1058)	-(39±105)	0.988
$\Delta\nu^{SS}$	2984±121	-(711±156)	354±54	1396±609	94±61	0.966
			Compound **3**			
ν_{ab}	23584±164	**-(1767±211)**	-(753±72)	-(2577±822)	121±82	0.988
ν_{fl}	20681±130	-(1505±167)	**-(1192±57)**	-(2142±650)	25±65	0.996
$\Delta\nu^{SS}$	2903±163	-(262±210)	439±72	-(435±818)	96±82	0.928
			Compound **4**			
ν_{ab}	23315±154	**-(1949±189)**	-(841±68)	-(1741±773)	6±77	0.990
ν_{fl}	20439±162	**-(2164±209)**	-(715±72)	-(1397±814)	27±81	0.987
$\Delta\nu^{SS}$	2877±53	215±68	-(125±23)	-(344±264)	-(21±26)	0.940
			Compound **5**			
ν_{ab}	23373±133	**-(1751±171)**	-(799±59)	-(1329±666)	31±66	0.991
ν_{fl}	20314±154	-(1159±198)	**-(1067±68)**	-(1989±771)	-(142±77)	0.993
$\Delta\nu^{SS}$	3059±150	-(592±193)	268±66	661±751	173±75	0.916
			Compound **6**			
ν_{ab}	23469±148	**-(1866±190)**	-(852±65)	106±741	229±74	0.987
ν_{fl}	20668±166	**-(1874±214)**	-(946±74)	111±834	147±83	0.987
$\Delta\nu^{SS}$	2801±40	7±52	95±18	-(4±201)	83±20	0.942
			Compound **7**			
ν_{ab}	23634±184	**-(1906±237)**	-(821±81)	-(1814±922)	91±92	0.985
ν_{fl}	19812±631	-(881±811)	**-(794±279)**	1167±3162	-(419±315)	0.738
$\Delta\nu^{SS}$	3822±503	-(1025±647)	-(26±222)	-(3482±2522)	511±251	0.617
			Compound **8**			
ν_{ab}	24892±233	**-(2480±300)**	-(921±130)	-(419±1169)	-(59±117)	0.979
ν_{fl}	20913±247	-(1533±317)	**-(1244±109)**	-(1936±1236)	-(127±123)	0.986
$\Delta\nu^{SS}$	3979±187	-(947±241)	323±83	1517±939	68±94	0.917

			Compound **9**			
ν_{ab}	23359±232	**-(2194±298)**	-(1014±103)	-(1569±1163)	181±116	0.982
ν_{fl}	20670±294	**-(2258±378)**	-(800±130)	-(1344±1472)	150±147	0.961
$\Delta\nu^{SS}$	2689±96	63±123	-(215±42)	-(225±481)	32±48	0.905
			Compound **10**			
ν_{ab}	23278±106	**-(1943±137)**	-(887±47)	-(1708±533)	136±53	0.995
ν_{fl}	20600±129	-(1521±166)	**-(1288±57)**	-(2124±648)	22±65	0.996
$\Delta\nu^{SS}$	2677±65	-(422±84)	401±29	417±328	114±33	0.989
			Compound **11**			
ν_{ab}	22593±72	**-(1720±92)**	-(888±32)	-(1453±359)	103±36	0.998
ν_{fl}	20081±111	-(1495±143)	**-(1049±49)**	-(1070±558)	-(66±56)	0.995
$\Delta\nu^{SS}$	2512±56	-(225±72)	161±25	-(384±279)	37±28	0.935
			Compound **12**			
ν_{ab}	22663±180	**-(1434±232)**	88±80	-(1333±903)	235±90	0.907
ν_{fl}	17969±779	318±1002	**-(3906±344)**	6256±3903	-(130±389)	0.974
$\Delta\nu^{SS}$	4695±929	-(1752±1196)	3994±411	-(7589±4659)	365±464	0.965
			Compound **13**			
ν_{ab}	22414±429	**-(1607±551)**	-(392±189)	-(2239±2149)	186±214	0.829
ν_{fl}	19738±661	-(617±850)	**-(1134±292)**	-(4083±3313)	-(249±330)	0.903
$\Delta\nu^{SS}$	2676±395	-(990±508)	742±174	1845±1978	435±197	0.917

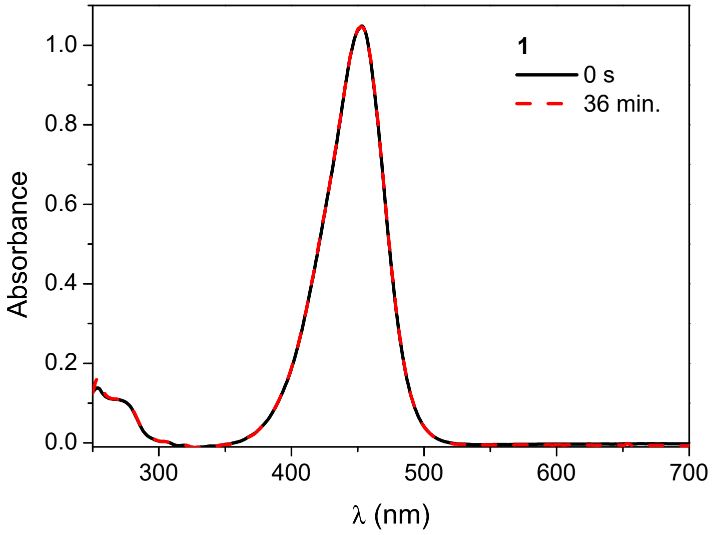

Figure S1. Changes of the electronic absorption spectra of **1** during an argon-ion laser irradiation (I_0 = 50 mW) in ethyl acetate. Time of irradiation marked in Figure.

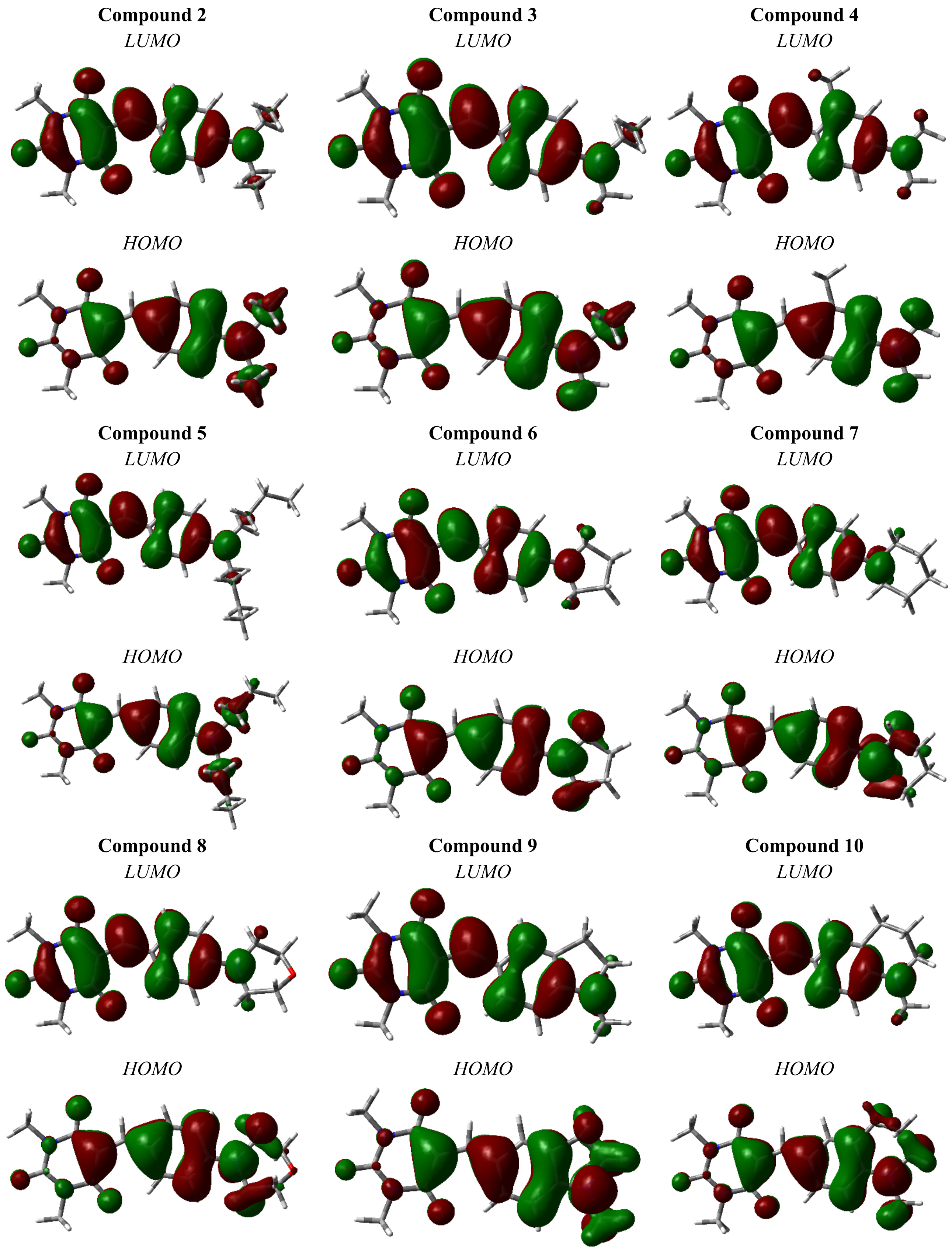

Figure S2. The HOMO – LUMO orbitals of selected merocyanine-type dyes.

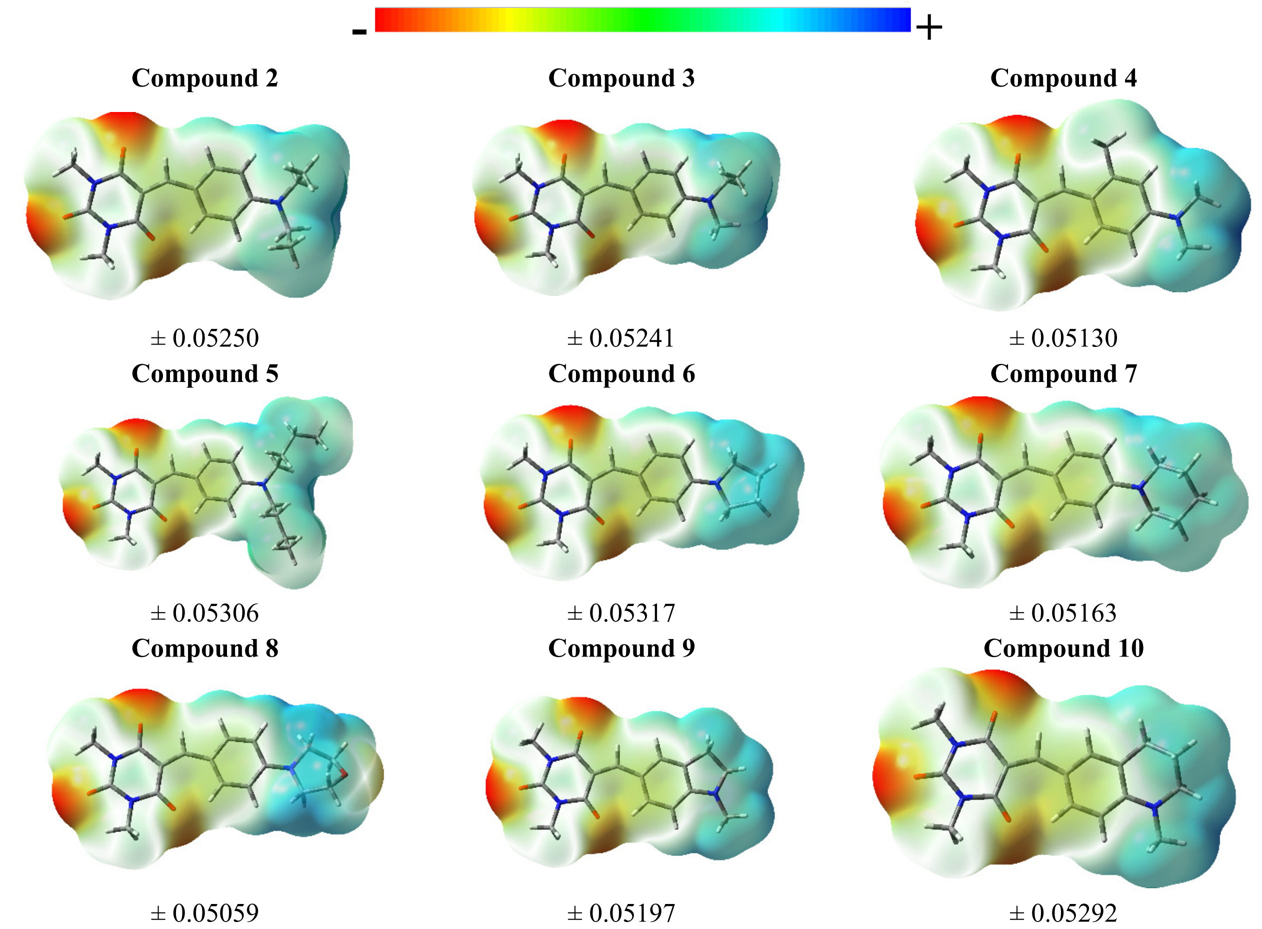

Figure S3. The MEP surfaces.

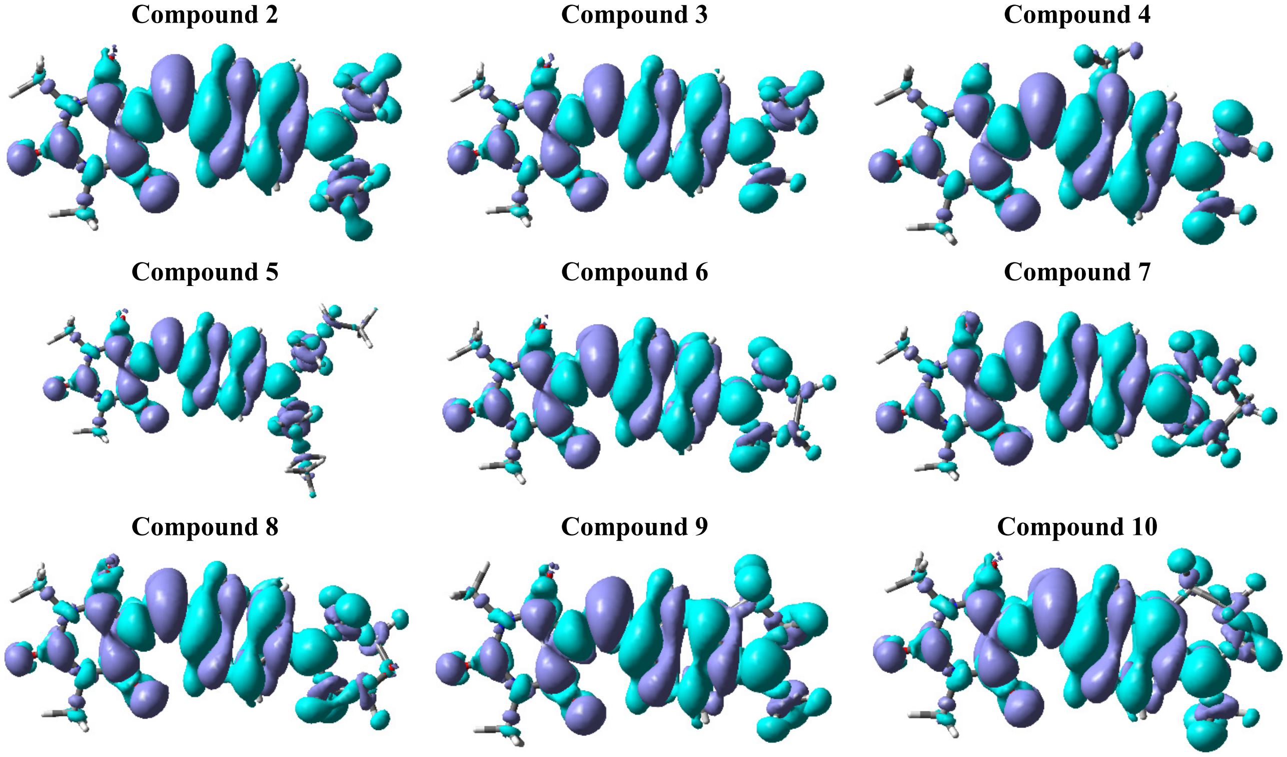

Figure S4. Density difference plot. The blue (purple) zones indicate density decrease (increase) upon electronic transition.

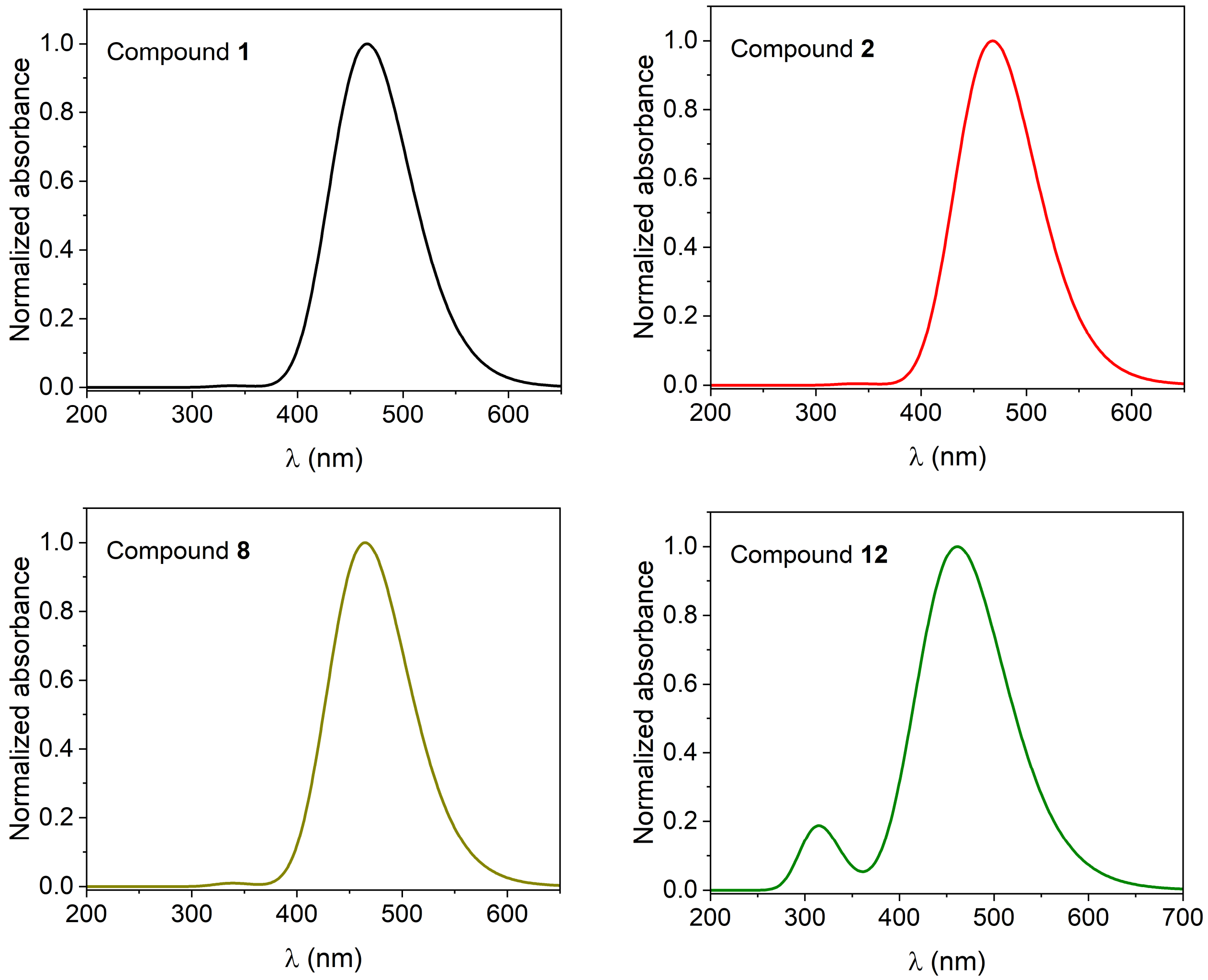

Figure S5. Graphical representation of the calculated absorption spectra for selected derivatives.

Table S3. Calculated geometries of the selected merocyanine dyes in THF

Compound **1**			Compound **12**		
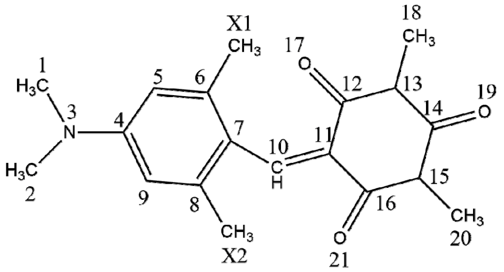					
**Bond**	**GS**	**ES**	**Bond**	**GS**	**ES**
C1-N3	1.45072	1.45477	C1-N3	1.42372	1.38923
C2-N3	1.45043	1.45454	C2-N3	1.42335	1.38995
N3-C4	1.35132	1.34409	N3-C4	1.38025	1.43871
C4-C5	1.42013	1.42613	C4-C5	1.41078	1.39516
C5-C6	1.37268	1.36687	C5-C6	1.37524	1.38078
C6-C7	1.41612	1.41637	C6-C7	1.41370	1.42194
C7-C8	1.41897	1.41634	C7-C8	1.41662	1.42483
C8-C9	1.37123	1.36684	C8-C9	1.37399	1.37800
C4-C9	1.41907	1.42620	C4-C9	1.40957	1.39685
C7-C10	1.42371	1.46555	C7-C10	1.43002	1.42223
C10=C11	1.38027	1.40161	C10=C11	1.37580	1.41007
C11-C12	1.45413	1.42870	C11-C12	1.45717	1.43645
C12-N13	1.39469	1.40271	C12-N13	1.39294	1.40727
N13-C14	1.38360	1.37817	N13-C14	1.38437	1.37647
C14-N15	1.38403	1.37848	C14-N15	1.38469	1.37870
N15-C16	1.38776	1.40315	N15-C16	1.38640	1.39889
C11-C16	1.47169	1.43672	C11-C16	1.47522	1.45006
C12=O17	1.22312	1.24076	C12=O17	1.22181	1.23348
N13-C18	1.45846	1.45434	N13-C18	1.45887	1.45537
C14=O19	1.21661	1.22788	C14=O19	1.21563	1.22482
N15-C20	1.45814	1.45462	N15-C20	1.45852	1.45523
C16=O21	1.22290	1.23500	C16=O21	1.22160	1.23203
**Dihedral angles**			**Dihedral angles**		
C1-N3-C4-C5	0.22361	7.77369	C1-N3-C4-C5	23.12534	78.98517
C1-N3-C4-C9	179.77579	172.21194	C1-N3-C4-C9	156.83584	101.03481
C2-N3-C4-C9	0.25148	8.01540	C2-N3-C4-C9	21.70378	78.91148
C2-N3-C4-C5	179.74792	172.04897	C2-N3-C4-C5	158.33504	101.66854
C7-C10=C11	138.95236	124.68649	C7-C10=C11	138.85884	135.69855

	Compound **5**			Compound **13**	
**Bond**	**GS**	**ES**	**Bond**	**GS**	**ES**
C1-N3	1.45772	1.46384	C1-N3	1.44821	1.45582
C2-N3	1.45788	1.46218	C2-N3	1.44732	1.45587
N3-C4	1.35483	1.34730	N3-C4	1.36205	1.34459
C4-C5	1.42204	1.42772	C4-C5	1.42618	1.42308
C5-C6	1.37210	1.36694	C5-C6	1.38563	1.37061
C6-C7	1.41608	1.41540	C6-C7	1.42064	1.42866
C7-C8	1.41880	1.41528	C7-C8	1.49899	1.42863
C8-C9	1.37065	1.36715	C8-C9	1.47743	1.37063
C4-C9	1.42094	1.42762	C4-C9	1.38883	1.42308
C6-X1			C6-X1	1.50419	1.49536
C8-X2			C8-X2	1.55092	1.49537
C7-C10	1.42258	1.46531	C7-C10	1.39882	1.46541
C10=C11	1.38105	1.40138	C10=C11	1.40122	1.40238
C11-C12	1.45349	1.42881	C11-C12	1.46073	1.43683
C12-N13	1.35502	1.40320	C12-N13	1.39616	1.40759
N13-C14	1.38345	1.37818	N13-C14	1.37953	1.37567
C14-N15	1.38395	1.37851	C14-N15	1.38053	1.37754
N15-C16	1.38827	1.40304	N15-C16	1.39800	1.40125
C11-C16	1.47104	1.43692	C11-C16	1.44632	1.42847
C12=O17	1.22322	1.24065	C12=O17	1.22624	1.23476
N13-C18	1.45837	1.45437	N13-C18	1.45705	1.45451
C14=O19	1.21674	1.22796	C14=O19	1.21940	1.22772
N15-C20	1.45813	1.45475	N15-C20	1.45675	1.45382
C16=O21	1.22307	1.23517	C16=O21	1.22826	1.24134
**Dihedral angles**			**Dihedral angles**		
C1-N3-C4-C5	0.81029	6.14506	C1-N3-C4-C5	2.13469	1.92009
C1-N3-C4-C9	179.89408	173.75373	C1-N3-C4-C9	177.34801	178.14492
C2-N3-C4-C9	2.59585	3.45524	C2-N3-C4-C9	1.80189	2.21464
C2-N3-C4-C5	178.10851	176.64597	C2-N3-C4-C5	177.68081	177.72034
C7-C10=C11	138.95586	125.01537	C7-C10=C11	135.53757	124.85573

Compound **8**

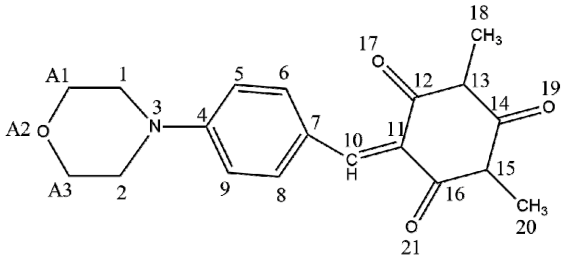

Compound **11**

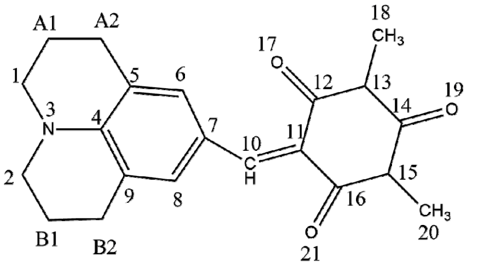

**Bond**	**GS**	**ES**	**Bond**	**GS**	**ES**
C1-N3	1.45735	1.46273	C1-N3	1.45599	1.46157
C2-N3	1.45685	1.46355	C2-N3	1.45583	1.46119
C1-CA1	1.51628	1.51670	C1-CA1	1.51481	1.51257
CA1-OA2	1.41823	1.41645	CA1-CA2	1.51870	1.51726
OA2-CA3	1.41870	1.41550	CA2-C5	1.50552	1.49839
CA3-C2	1.51625	1.51671	C2-CB1	1.51505	1.51236
N3-C4	1.35338	1.34390	CB1-CB2	1.51895	1.51761
C4-C5	1.41906	1.42653	CB2-C9	1.50526	1.49898
C5-C6	1.37302	1.36652	N3-C4	1.35663	1.34644
C6-C7	1.41563	1.41688	C4-C5	1.42754	1.43674
C7-C8	1.41838	1.41702	C5-C6	1.37355	1.37061
C8-C9	1.37148	1.36654	C6-C7	1.41505	1.41062
C4-C9	1.41797	1.42647	C7-C8	1.41897	1.41047
C7-C10	1.42459	1.46497	C8-C9	1.37181	1.37089
C10=C11	1.37945	1.40130	C4-C9	1.42649	1.43688
C11-C12	1.45471	1.43657	C7-C10	1.42012	1.46780
C12-N13	1.39431	1.40308	C10=C11	1.38485	1.40273
N13-C14	1.38369	1.37870	C11-C12	1.45250	1.43800
C14-N15	1.38407	1.37849	C12-N13	1.39527	1.440737
N15-C16	1.38769	1.40196	N13-C14	1.38151	1.37521
C11-C16	1.47235	1.42829	C14-N15	1.38086	1.37638
C12=O17	1.22295	1.23495	N15-C16	1.39245	1.40368
N13-C18	1.45848	1.45470	C11-C16	1.47097	1.43055
C14=O19	1.21635	1.22769	C12=O17	1.22397	1.23536
N15-C20	1.45830	1.45454	N13-C18	1.45756	1.45435
C16=O21	1.22262	1.24105	C14=O19	1.21758	1.22841
			N15-C20	1.45801	1.45362
			C16=O21	1.22346	1.23955
**Dihedral angles**			**Dihedral angles**		
C1-N3-C4-C5	9.34885	8.91062	C1-N3-C4-C5	5.20762	2.12992
C1-N3-C4-C9	170.69096	171.18084	C1-N3-C4-C9	175.32794	177.60016
C2-N3-C4-C9	9.49233	7.86765	C2-N3-C4-C9	5.56457	3.49860
C2-N3-C4-C5	170.46786	172.04088	C2-N3-C4-C5	174.97100	176.23148
C7-C10=C11	138.92993	124.62726	C7-C10=C11	139.13035	125.43461

Table S4. The frontier orbital energies in selected solvents. All values are given in eV

	GP	Toluene	THF	DMF		GP	Toluene	THF	DMF
Compound **1**					Compound **2**				
E_{HOMO}	-6.0412	-6.0145	-6.0140	-6.0181	E_{HOMO}	-5.9903	-5.9786	-5.9884	-5.9979
E_{LUMO}	-2.3254	-2.4054	-2.4792	-2.5167	E_{LUMO}	-2.2998	-2.3875	-2.4661	-2.5069
E_{GAP}	3.7158	3.6091	3.5348	3.5013	E_{GAP}	3.6905	3.5911	3.5223	3.4910
η	1.8579	1.8046	1.7674	1.7507	η	1.8452	1.7956	1.7611	1.7455
μ	-4.1833	-4.2100	-4.2466	-4.2674	μ	-4.1451	-4.1830	-4.2273	-4.2524
χ	4.1833	4.2100	4.2466	4.2674	χ	4.1451	4.1830	4.2273	4.2524
Compound **3**					Compound **4**				
E_{HOMO}	-6.0026	-5.9824	-5.9868	-5.9938	E_{HOMO}	-5.9696	-5.9604	-5.9718	-5.9819
E_{LUMO}	-2.3045	-2.3886	-2.4650	-2.5045	E_{LUMO}	-2.3292	-2.4117	-2.4882	-2.5282
E_{GAP}	3.6981	3.5939	3.5217	3.4894	E_{GAP}	3.6404	3.5487	3.4836	3.4537
η	1.8490	1.7969	1.7609	1.7447	η	1.8202	1.7743	1.7418	1.7269
μ	-4.1535	-4.1855	-4.2259	-4.2492	μ	-4.1494	-4.1860	-4.2300	-4.2550
χ	4.1535	4.1855	4.2259	4.2492	χ	4.1494	4.1860	4.2300	4.2550
Compound **5**					Compound **6**				
E_{HOMO}	-5.9370	-5.9457	-5.9623	-5.9729	E_{HOMO}	-5.9522	-5.9438	-5.9541	-5.9626
E_{LUMO}	-2.2710	-2.3758	-2.4599	-2.5012	E_{LUMO}	-2.2713	-2.3668	-2.4490	-2.4901
E_{GAP}	3.6660	3.5699	3.5024	3.4717	E_{GAP}	3.6809	3.5770	3.5051	3.4725
η	1.8330	1.7850	1.7512	1.7358	η	1.8405	1.7885	1.7526	1.7362
μ	-4.1040	-4.1607	-4.2111	-4.2371	μ	-4.1117	-4.1553	-4.2015	-4.2263
χ	4.1040	4.1607	4.2111	4.2371	χ	4.1117	4.1553	4.2015	4.2263
Compound **7**					Compound **8**				
E_{HOMO}	-6.0466	-6.0028	-5.9381	-5.9274	E_{HOMO}	-6.1190	-6.0801	-6.0630	-6.0567
E_{LUMO}	-2.3736	-2.4400	-2.4593	-2.4805	E_{LUMO}	-2.3932	-2.4544	-2.5094	-2.5369
E_{GAP}	3.6731	3.5628	3.4787	3.4469	E_{GAP}	3.7258	3.6257	3.5536	3.5198
η	1.8365	1.7814	1.7394	1.7235	η	1.8629	1.8129	1.7768	1.7599
μ	-4.2101	-4.2214	-4.1987	-4.2040	μ	-4.2561	-4.2673	-4.2862	-4.2968
χ	4.2101	4.2214	4.1987	4.2040	χ	4.2561	4.2673	4.2862	4.2968
Compound **9**					Compound **10**				
E_{HOMO}	-5.9536	-5.8923	-5.8605	-5.8491	E_{HOMO}	-5.8932	-5.8657	-5.8678	-5.8747
E_{LUMO}	-2.3249	-2.3853	-2.4419	-2.4716	E_{LUMO}	-2.2666	-2.3483	-2.4258	-2.4667
E_{GAP}	3.6287	3.5070	3.4186	3.3775	E_{GAP}	3.6265	3.5174	3.4420	3.4080
η	1.8143	1.7535	1.7093	1.6888	η	1.8133	1.7587	1.7210	1.7040
μ	-4.1392	-4.1388	-4.1512	-4.1603	μ	-4.0799	-4.1070	-4.1468	-4.1707
χ	4.1392	4.1388	4.1512	4.1603	χ	4.0799	4.1070	4.1468	4.1707

Compound **11**					Compound **12**				
E_{HOMO}	-5.7805	-5.7612	-5.7696	-5.7794	E_{HOMO}	-5.9136	-5.9182	-5.9375	-5.9522
E_{LUMO}	-2.2283	-2.3132	-2.3943	-2.4373	E_{LUMO}	-2.5467	-2.6065	-2.6653	-2.6931
E_{GAP}	3.5522	3.4480	3.3753	3.3421	E_{GAP}	3.3669	3.3117	3.2722	3.2591
η	1.7761	1.7240	1.6877	1.6711	η	1.6834	1.6558	1.6361	1.6296
μ	-4.0044	-4.0372	-4.0819	-4.1083	μ	-4.2301	-4.2624	-4.3014	-4.3226
χ	4.0044	4.0372	4.0819	4.1083	χ	4.2301	4.2624	4.3014	4.3226
Compound **13**									
E_{HOMO}	-5.7993	-5.7936	-5.8112	-5.8273					
E_{LUMO}	-2.2827	-2.3774	-2.4634	-2.5094					
E_{GAP}	3.5166	3.4162	3.3478	3.3179					
η	1.7583	1.7081	1.6739	1.6590					
μ	-4.0410	-4.0855	-4.1373	-4.1683					
χ	4.0410	4.0855	4.1373	4.1683					

Table S5. CT parameters for the bright low-lying excited state

**Compound**	**GP**		**Toluene**		**THF**		**DMF**	
	q_{CT}	D_{CT}	q_{CT}	D_{CT}	q_{CT}	D_{CT}	q_{CT}	D_{CT}
**1**	0.443	2.134	0.451	2.499	0.458	2.371	0.449	2.550
**2**	0.451	2.074	0.466	2.300	0.458	2.415	0.457	2.477
**3**	0.445	2.132	0.458	2.351	0.451	2.471	0.449	2.525
**4**	0.425	1.912	0.437	2.135	0.429	2.230	0.428	2.272
**5**	0.466	2.187	0.479	2.379	0.472	2.500	0.470	2.567
**6**	0.445	2.096	0.458	2.330	0.449	2.433	0.447	2.489
**7**	0.475	2.267	0.481	2.485	0.454	2.512	0.449	2.516
**8**	0.458	2.224	0.471	2.456	0.464	2.579	0.465	2.622
**9**	0.444	2.175	0.450	2.413	0.437	2.522	0.432	2.565
**10**	0.443	2.104	0.451	2.317	0.440	2.425	0.437	2.468
**11**	0.444	1.974	0.447	2.182	0.432	2.284	0.427	2.315
**12**	0.577	3.142	0.588	3.484	0.590	3.741	0.593	3.844
**13**	0.558	2.212	0.546	2.498	0.525	2.636	0.517	2.710

Table S6. The values of free energies ($\Delta Gsolv$) of solvation in kcal/mol obtained using SMD solvation model

Compound	ΔG_{solv}		
	**Toluene**	**THF**	**DMF**
1	-17.21	-19.19	-19.13
2	-18.68	-20.00	-20.92
3	-17.32	-20.24	-21.52
4	-16.34	-17.80	-18.88
5	-19.38	-22.20	-24.80
6	-17.74	-20.23	-21.24
7	-18.37	-20.67	-20.61
8	-19.78	-20.48	-22.75
9	-18.08	-21.26	-23.39
10	-18.45	-21.40	-22.43
11	-19.70	-22.46	-22.00
12	-22.33	-24.06	-24.14
13	-16.74	-19.48	-20.21

Table S7. The values of vertical excitation energies (in nm)

Compound	GAZ		Toluene		THF		DMF	
	λ_{max}^{Ab}	f	λ_{max}^{Ab}	f	λ_{max}^{Ab}	f	λ_{max}^{Ab}	f
**1**	424.22	1.0137	453.35	1.2007	457.13	1.1973	465.89	1.2122
**2**	427.74	1.0664	455.81	1.2524	459.33	1.2506	467.89	1.2657
**3**	426.21	1.0482	454.97	1.2371	458.66	1.2363	467.30	1.2518
**4**	433.29	0.9694	461.71	1.1576	464.59	1.1553	473.02	1.1710
**5**	430.87	1.1121	458.27	1.2879	461.92	1.2891	470.53	1.3051
**6**	428.06	1.1010	457.27	1.2885	460.83	1.2882	469.56	1.3035
**7**	430.32	1.0931	459.18	1.2938	463.07	1.3428	471.26	1.3618
**8**	424.40	1.0861	452.22	1.2699	456.06	1.2707	464.79	1.2856
**9**	434.40	0.9212	465.41	1.1183	470.73	1.1196	480.30	1.1389
**10**	433.49	0.9755	463.09	1.1765	467.03	1.1759	475.92	1.1929
**11**	440.84	0.9695	470.39	1.1804	473.89	1.1852	482.58	1.2056
**12**	436.54	0.9716	459.77	1.1809	458.83	1.1823	460.74	1.2011
**13**	446.92	0.9649	471.16	0.9497	470.90	0.9694	479.50	0.9928

Table S8. The values of cLR corrected excitation energies (in nm)

**Compound**	λ^{Ab}_{cLR}		
	**Toluene**	**THF**	**DMF**
**1**	443.19	444.55	455.24
**2**	446.06	447.12	457.65
**3**	444.93	446.13	456.70
**4**	450.44	451.02	461.30
**5**	449.22	450.43	461.08
**6**	446.56	447.68	458.28
**7**	449.73	450.11	460.02
**8**	433.19	434.56	445.29
**9**	455.23	457.85	469.08
**10**	452.47	453.86	464.55
**11**	458.98	459.91	470.27
**12**	450.12	447.94	450.02
**13**	465.00	462.30	470.69

Table S9. Calculated values of dipole moments (in D) for the ground and CT excited state

**Compound**	**GP**		**Toluene**		**THF**		**DMF**	
	μ_g	μ_{CT}	μ_g	μ_{CT}	μ_g	μ_{CT}	μ_g	μ_{CT}
**1**	7.93	12.46	9.63	14.80	10.94	16.31	11.57	17.03
**2**	8.16	12.66	9.89	15.04	11.22	16.56	11.87	17.30
**3**	8.14	12.66	9.90	15.04	11.26	16.56	11.90	17.29
**4**	7.95	11.81	9.69	14.10	11.06	15.60	11.73	16.34
**5**	8.47	13.34	10.13	15.60	11.42	17.08	12.05	17.82
**6**	8.74	13.21	10.54	15.61	11.92	17.14	12.59	17.87
**7**	7.72	12.87	9.53	15.25	11.66	17.08	12.62	18.03
**8**	6.16	11.03	7.61	13.15	8.73	14.49	9.27	15.14
**9**	7.73	12.36	9.70	14.89	11.40	16.67	12.29	17.57
**10**	8.42	12.87	10.42	15.41	11.98	17.08	12.75	17.90
**11**	8.51	12.67	10.60	15.28	12.31	17.02	13.19	17.90
**12**	6.36	15.05	8.25	17.57	8.71	18.77	9.14	20.05
**13**	6.41	12.38	7.95	14.50	9.12	15.77	9.68	16.40

Table S10. The values of vertical and cLR corrected de-excitation energies (in nm)

Compound	GP	Toluene		THF		DMF	
	λ_{max}^{Fl}	λ_{max}^{Fl}	$\lambda_{cl.R}^{Fl}$	λ_{max}^{Fl}	$\lambda_{cl.R}^{Fl}$	λ_{max}^{Fl}	$\lambda_{cl.R}^{Fl}$
**1**	500.68	517.30	530.00	525.57	535.90	537.13	545.34
**2**	507.31	525.37	537.29	531.55	541.13	538.08	553.27
**3**	500.06	521.20	534.38	533.71	541.14	536.09	552.86
**4**	528.45	538.48	527.12	539.81	526.73	556.53	566.03
**5**	506.52	522.99	532.21	535.90	549.72	542.12	555.70
**6**	504.58	526.96	541.89	539.68	547.34	540.37	549.17
**7**	503.84	532.11	546.79	555.03	568.69	544.87	551.76
**8**	496.02	507.15	518.37	514.19	524.08	523.66	536.60
**9**	516.86	526.77	539.38	538.19	551.92	526.04	535.08
**10**	514.15	524.76	538.80	540.98	552.96	550.27	564.57
**11**	507.57	528.75	541.23	545.60	555.72	553.53	564.12
**12**	563.34	576.70	587.45	631.42	648.79	651.90	664.04
**13**	506.56	514.73	525.59	549.45	563.13	558.83	572.02
